# Photochemistry of Cannabidiol (CBD) Revised. A Combined Preparative and Spectrometric Investigation

**DOI:** 10.1021/acs.jnatprod.1c00567

**Published:** 2021-10-20

**Authors:** Paolo Seccamani, Chiara Franco, Stefano Protti, Alessio Porta, Antonella Profumo, Diego Caprioglio, Stefano Salamone, Barbara Mannucci, Daniele Merli

**Affiliations:** † Department of Chemistry, University of Pavia, Viale Taramelli 10, 27100 Pavia, Italy; ‡ INFN Sezione di Milano-Bicocca, Piazza della Scienza 3, 20126 Milano, Italy; § Department of Pharmaceutical Sciences, University of Piemonte Orientale, 28100 Novara, Italy; ∥ Centro Grandi Strumenti, University of Pavia, Via Bassi 21, 27100 Pavia, Italy

## Abstract

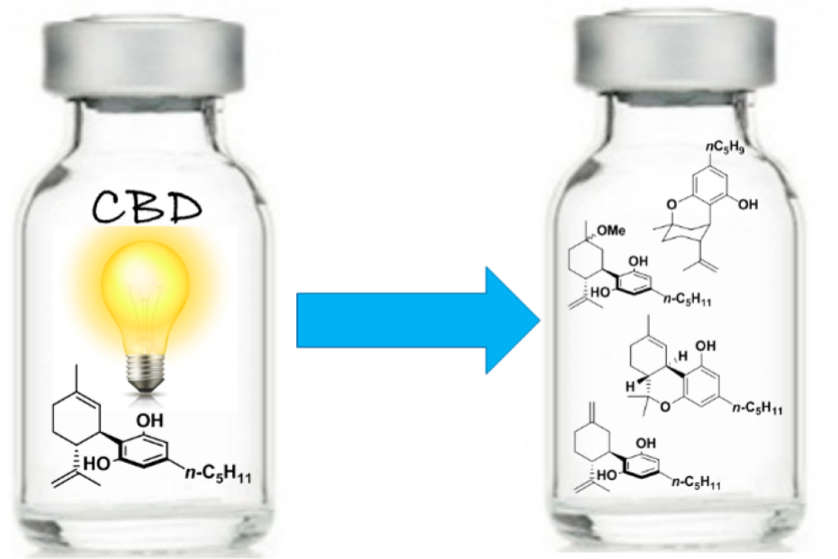

Cannabis is a plant with an astonishing ability to biosynthesize cannabinoids, and more than 100 molecules belonging to this class have been isolated. Among them in recent years cannabidiol (CBD) has received the interest of pharmacology as the major nonpsychotropic cannabinoid with many potential clinical applications. Although the reactivity of CBD has been widely investigated, only little attention has been given to the possible photodegradation of this cannabinoid, and the data available in the literature are outdated and, in some cases, conflicting. The aim of the present work is providing a characterization of the photochemical behavior of CBD in organic solvents, through a detailed GC-MS analyses, isolation, and NMR characterization of the photoproducts obtained.

The Cannabinaceae family can be classified into three main species, namely, *Cannabis ruderalis* Janish, *Cannabis sativa* L., and *Cannabis indica* Lam.^1^ The latter two are of psychotropic and clinical interest, thanks to their significant content of cannabinoids, especially cannabidiol (CBD) and Δ^9^-tetrahydrocannabinol (Δ^9^-THC, see below).^1^ Between these varieties, the main difference is the content of the psychoactive molecule Δ^9^-THC (**1**).^2^ Nevertheless, of the more than 100 cannabinoids that have been isolated, Δ^9^-THC (**1**), identified by Raphael Mechoulam in 1964, has been the focus of all the pharmacological attention as the main “active” principle excluding virtually all the other cannabinoids.^3^ In recent years, however, cannabidiol (**2**) dramatically emerges as the major nonpsychotropic cannabinoid with many potential therapeutic benefits,^4^ due to its regulatory role on the effect of Δ^9^-THC (**1**), its role as a strong antagonist of cannabinoid receptor type 1 (CB1),^5^ and its use in the treatment of neurological diseases and other applications of therapeutic relevance.^6,7^

Although there are studies carried out concerning the stability of **1** both as a pure compound and in different formulations, little attention has been given to **2** and its possible degradation products. Furthermore, the conversion of CBD into Δ^9^-THC (**1**) is still controversial.^8^ Studies carried out exposing the natural resinous matrix (hashish) for a prolonged time to light at atmospheric temperature showed only a decrease in the concentration of Δ^9^-THC (**1**) with the concentration of CBD (**2**)^9^ being unchanged for 4 years. On the other hand, Lydon and Teramura^10^ evaluated the photoreactivity of *C. sativa* extracts under UV and visible light irradiation, observing a decrease in the concentration of **2** and no alteration in the amount of **1** or cannabichromene (CBC, **3**) excluding any possible risk of photochemical conversion of **2** into **1** in a natural matrix. The first attempts at photochemical studies on CBD (**2**) were performed by Loewe in 1950.^11^ In 1971, Shani and Mechoulam identified some of the products resulting from the UV irradiation of CBD (**2**) in MeOH and cyclohexane, including Δ^9^-THC (**1**).^12^ Allward et al.^13,14^ tested the reactivity of different cannabinoids, including CBD (**2**) and Δ^8^-*trans*-THC (**4**), upon irradiation in the 235–285 nm region, but no photochemical interconversion was observed between the latter two. Thus, although the reactivity of **2** to light has been unanimously stated, more consistent data concerning this topic are needed. Moreover, a systematic investigation of the structure and the distribution of the photoproducts as well as the influence of different parameters important for the photoreaction is still lacking.^11−14^ We present herein a characterization of the photophysics and the photochemical behavior of CBD (**2**) in different organic solvents by means of GC-MS analyses, isolation, and NMR characterization of the photoproducts obtained. The work is also aimed at verifying the stability of CBD (**2**) in solvent formulations or during the extraction step from plant raw material before it is administered to animals or humans. In this way one could discriminate the compounds or degradation artifacts from the products coming from their *in vivo* metabolism.
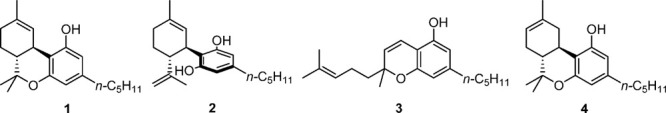


## Results and Discussion

### GC-MS Characteristic of the Analyzed Cannabinoids

The products investigated in the present work have been characterized by comparison with authentic samples or by isolation via column chromatography from the photolyzed mixture (see the Supporting Information for further details). GC-MS data obtained for the cannabinoids and the corresponding trimethylsilyl derivatives are summarized in Table 2.1 (see Supporting Information). As expected, we noticed that, in most cases, the fragmentation spectra of derivatized products are similar to those of nonderivatized ones, and often the entire spectrum is simply translated by 72 *m*/*z* or by 144 *m*/*z*, depending on the number of Me_3_Si– groups bound to each molecule.
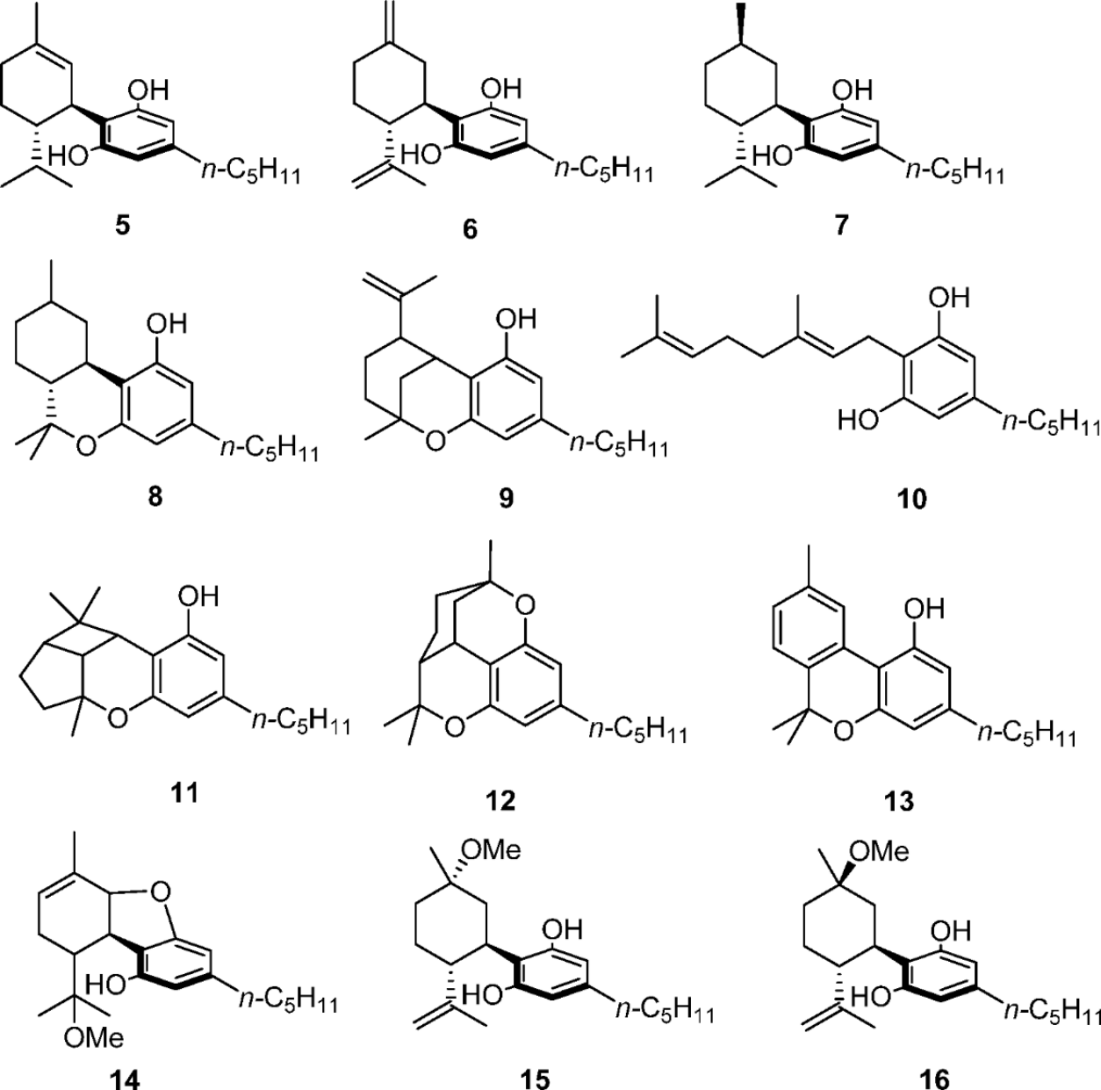


### Photophysics of CBD

CBD (**2**) exhibits two absorption maxima in the 210–220 and 270–280 nm regions and emission in the 290–300 nm region, with a fluorescence quantum yield (Φ_F_) between 0.006 and 0.012, depending on the solvent (see Table 1). The emission spectra of CBD (**2**) in MeOH and MeCN consist of a signal in the 290–300 nm region that, according to the literature,^15^ has been assigned to the emission from the lowest lying singlet state of the phenolic moiety of CBD (**2**). The excitation spectra corresponding to these fluorescence features are very similar to the absorption spectrum (see the Supporting Information, Section 10). In *n*-hexane, along with the 300 nm emission, a shoulder in the 310–340 nm region has been observed, with the excitation spectra recorded at the two emission maxima being slightly different.

**Table 1:** Photophysics of CBD (**2**): Absorption Maxima (λ_abs_) and Fluorescence Quantum Yield (λ_em_)

solvent	λ_abs_ (nm), ε (M^–1^ cm^–1^)	λ_em_ (nm), Φ_F_^a^
MeCN	208, 42 000	297, 0.008
	275, 814	
MeOH	210, 38 170	299, 0.012
	275, 1014	
*n*-hexane	207, 21 040	296, 0.006
	276, 746	

a 4-Chloroanisole (Φ_F_ = 0.019 in MeOH, λ_em_ = 273 nm) has been used as a reference.^16^ The quantum yield values have been corrected for the refractive index of the solvent.

The results we obtained are referred to the irradiation at 254 nm. We have checked the effect of the wavelength of irradiation on the photoproduct nature and distribution by irradiations at 310 nm (10 lamps × 15 W each). In all cases, the nature and distribution of products are similar, although the conversion of CBD is lower (in all cases, <45% for 50 min of irradiation; see the Supporting Information, Section 7).

### Photoreactivity of CBD in the Examined Solvents

The photoreactivity of CBD (**2**) was examined in three different solvents, namely, MeCN, MeOH, and *n*-hexane.

The sensitivity of CBD (**2**) to UV light in MeCN was evidenced by the measured consumption quantum yield (Φ_–1_ = 0.058) and a rate constant value of 4.3 × 10^–4^ s^–1^. A solution of CBD (**2**, 1.27 × 10^–3^ M) was irradiated at 254 nm at increasing times, and the reaction course analyzed by GC-MS. The structure of the generated compounds was elucidated based on their fragmentation spectra and by comparison with authentic samples.

**Figure 1:**
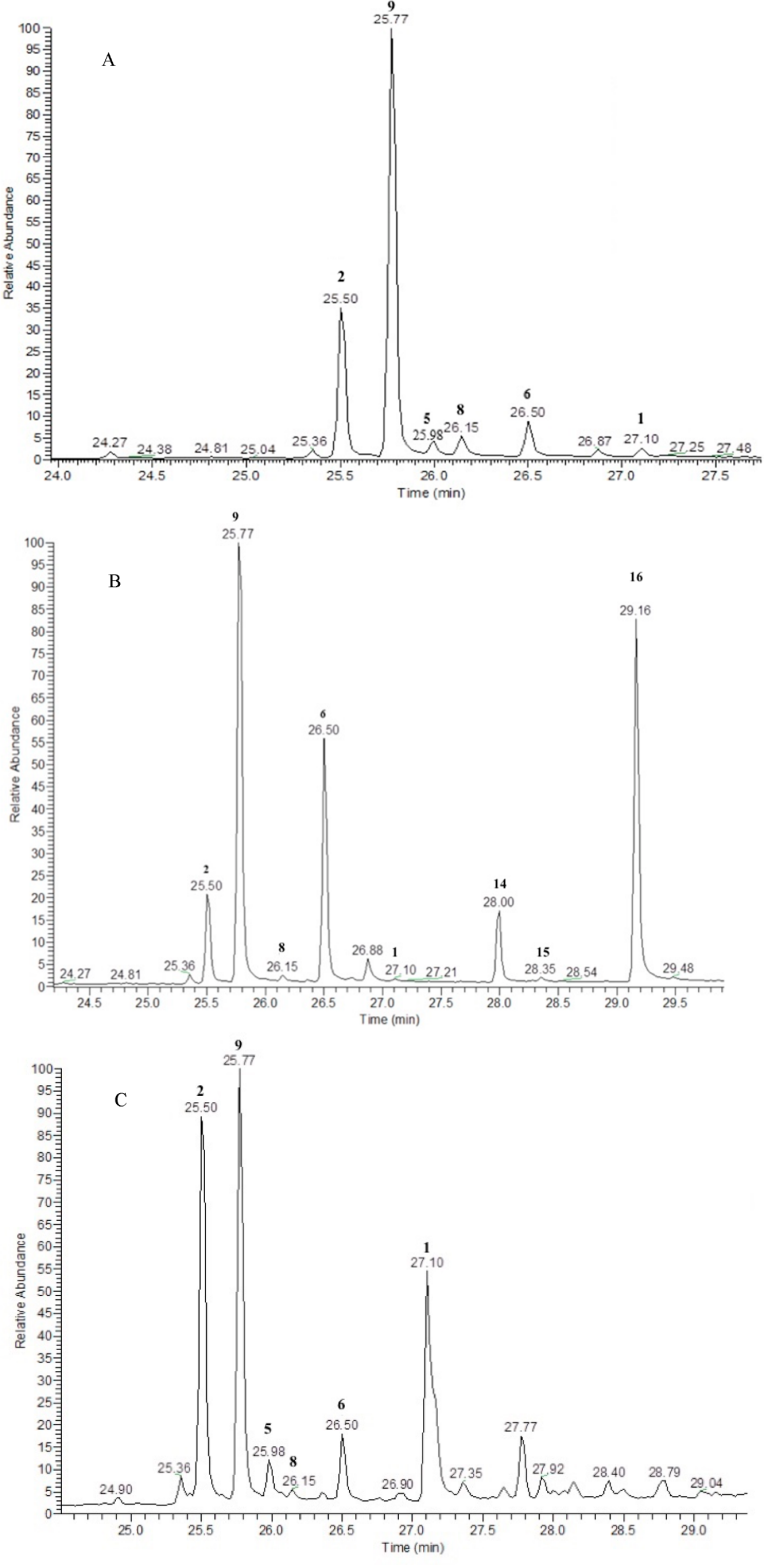
(A) GC-MS chromatogram of 1.27 × 10^–3^ M CBD (**2**) in MeCN irradiated for 50 min (λ = 254 nm). The main photoproducts detected are Δ^8^-*iso*-THC (**9**, 39.7% yield, *t*_R_ 25.77 min), DHD (**5**, 2.7% yield, *t*_R_ 25.98 min), HHC (**8**, 4.3% yield, *t*_R_ 26.15 min), Δ^7^-CBD (**6**, 7.2% yield, *t*_R_ 26.5 min), Δ^9^-THC (**1**, 2.4% yield, *t*_R_ 27.1 min). (B) GC-MS chromatogram of 1.27 × 10^–3^ M CBD (**2**) in MeOH irradiated for 50 min (λ = 254 nm). The main photoproducts detected are Δ^8^-iso-THC (**9**, 35.9% yield, *t*_R_ 25.77 min), HHC (**8**, 2.0% yield, *t*_R_ 26.15 min), Δ^7^-CBD (**6**, 18.3% yield, *t*_R_ 26.5 min), Δ^9^-THC (**1**, 1.3%, yield, *t*_R_ 27.1 min), MeO-CBE (**14**, 7.0% yield, *t*_R_ 28.0 min), α-MeO (**15**, 0.6% yield, *t*_R_ 25.35 min), β-MeO (**16**, 25.0% yield, *t*_R_ 29.16 min). (C) GC-MS chromatogram of 1.27 × 10^–3^ M CBD (**2**) in hexane irradiated for 50 min (λ = 254 nm). The main photoproducts detected are Δ^8^-*iso*-THC (**9**, 24.4% yield, *t*_R_ 25.77 min), DHD (**5**, 5.9% yield, *t*_R_ 25.98 min), HHC (**8**, 1.6% yield, *t*_R_ 26.15 min), Δ^7^-CBD (**6**, 6.2% yield, *t*_R_ 26.5 min), Δ^9^-THC (**1**, 11.3% yield, *t*_R_ 27.1 min). It should be noticed that the peaks at *t*_R_ 26.88 min (in chromatogram A) and 27.22 (in chromatogram C) were not identified either with the available libraries or by comparison of the synthesized standards.

**Figure 2:**
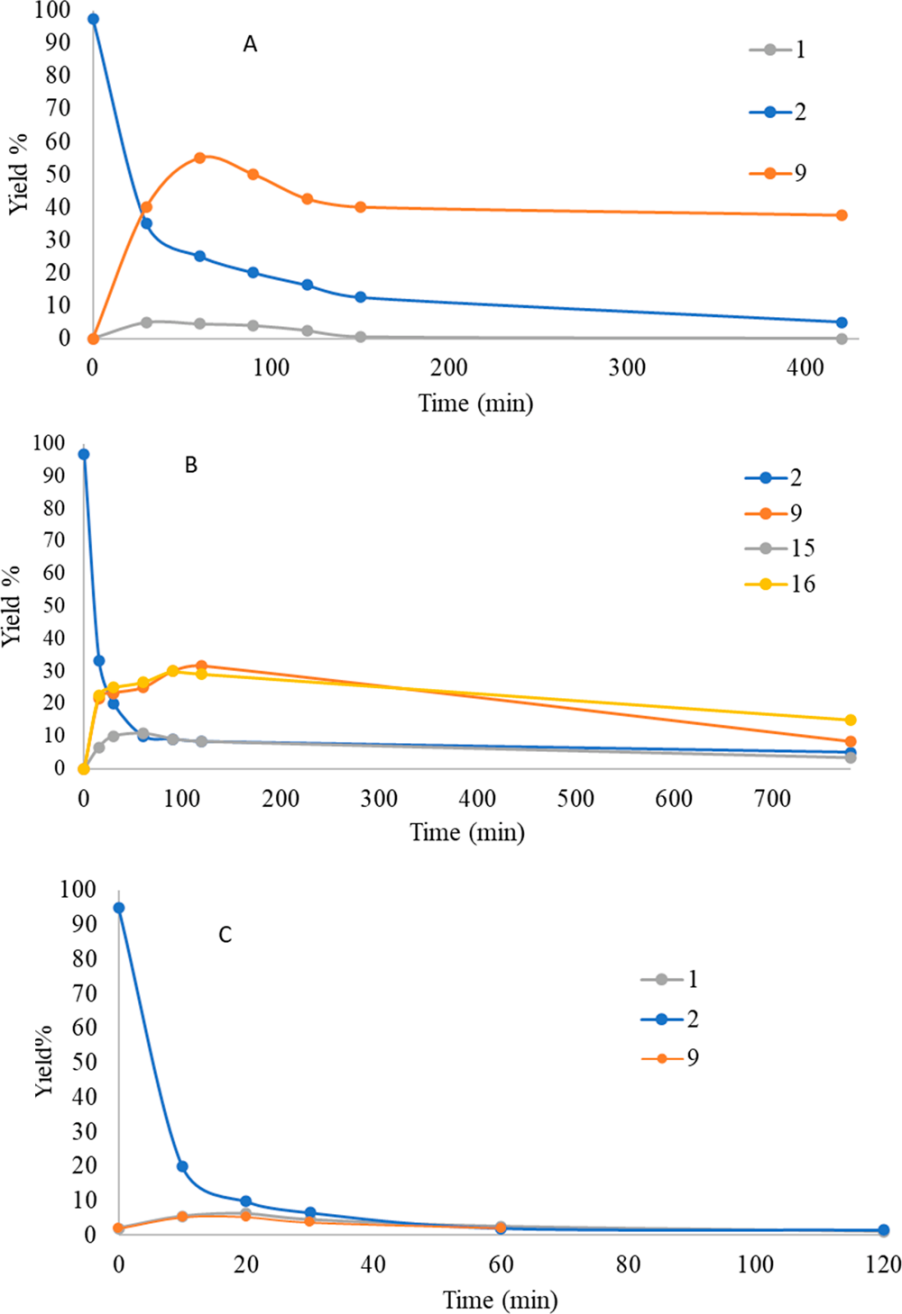
Photodegradation of CBD (**2**) in (A) MeCN, (B) MeOH, and (C) *n*-hexane.

As shown in Figure 1a, the following photoproducts have been observed: Δ^8^-*iso*-THC (**9**, 39.7% yield, *t*_R_ 25.77 min), DHD (**5**, 2.7% yield, rt 25.98 min), HHC (**8**, 4.3% yield, *t*_R_ 26.15 min), Δ^7^-CBD (**6**, 7.2% yield, *t*_R_ 26.5 min), Δ^9^-THC (**1**, 2.4% yield, *t*_R_ 27.1 min). Kinetic analyses (Figure 2a) pointed out that, simultaneously to the disappearance of CBD (**2**), Δ^8^-*iso*-THC (**9**) and Δ^9^-THC (**1**) arose as the main photoproducts. However, after an initial increase with a maximum corresponding to 53% yield, the concentration of Δ^8^-*iso*-THC (**9**) decreased, probably due to its own photodegradation. Similarly, Δ^9^-THC (**1**) reached a maximum of 4.7% yield compared to the initial CBD (**2**) value, but its concentration dropped below 0.1% beyond 150 min of irradiation.

The measured values for the quantum yield (Φ_–1_ = 0.055) and the rate constant (6.2 × 10^–4^ s^–1^) of CBD (**2**) in MeOH were comparable to those found in MeCN. Apart from the photoproducts observed in aprotic medium (see a representative chromatogram in Figure 1b), GC-MS analyses highlighted the presence of two other compounds (*t*_R_ 28.35 and 29.12 min, respectively) that have been isolated by column chromatography, characterized by analysis of spectroscopic data, and identified as the α-methoxy-CBD (α-MeO, **15**) and β-methoxy-CBD (β-MeO, **16**) diastereoisomers.

The kinetics of CBD (**2**) photodegradation in MeOH (Figure 2b) follows the same trend found with MeCN. Consumption of the substrate is generally fast (*t*_1/2_ = 15 min under the irradiation conditions). The maximum concentration of analytes is reached after only a few minutes of irradiation, with the only exception of Δ^8^-*iso*-THC (**9**), which exhibits a slower increase with a maximum corresponding to a yield of 32% after 2 h of irradiation. On the other hand, the two isomers α-MeO (**15**) and β-MeO (**16**) have a parallel trend, with maximum concentration observable at 60 min (0.6% and 25.0% yield, respectively) and then gradually decreasing.

High irradiation times also led to an almost complete photochemical degradation of the products, except for Δ^8^-*iso*-THC (**9**), which is still present after 15 h of irradiation in 13% yield. No formation of cannabicyclol (**11**) was observed.^17^

The measured disappearance quantum yield (Φ_–1_) value of CBD (**2**) in hexane is 0.087, with a rate constant value of 1.4 × 10^–3^ s^–1^. The chromatogram shown in Figure 1c corresponds to the fraction irradiated in *n-*hexane for 50 min. The choice to study this irradiation time is due to the approximately equal signal of all the main peaks found. The extremely rapid breakdown of CBD (**2**) into *n-*hexane yielded only a few easily recognizable products, which were identified as follows: Δ^8^-*iso*-THC (**9**, 24.4% yield, *t*_R_ 25.77 min), DHD (**5**, 6.2% yield, *t*_R_ 25.98 min), HHC (**8**, 1.6% yield, *t*_R_ 26.15 min), Δ^7^-CBD (**6**, 5.9% yield, *t*_R_ 26.5 min), and Δ^9^-THC (**1**, 11.3% yield, *t*_R_ 27.1 min).

As hinted above, the kinetics of CBD (**2**) in *n-*hexane (Figure 2c) under irradiation turned out to be very different from those observed in the other two media, with an extremely rapid degradation of CBD (**2**) (*t*_1/2_ = 5 min in our experimental conditions). Indeed, within the first 60 min of irradiation, the CBD (**2**) and all the formed photodegradation products disappear completely. The two products found were Δ^8^-*iso*-THC (**9**) and Δ^9^-THC (**1**), each obtained in up to 5% yield. No incorporation of the solvent was observed, contrary to what was previously reported by Shani and Mechoulam.^12^

In all cases, we found <0.1% conversion to cannabichromene (**3**), cannabicyclol (**11**), cannabigerol (**10**), cannabinol (**13**), cannabicitran (**12**), and tetrahydrocannabidiol (**7**).

**Scheme 1:**
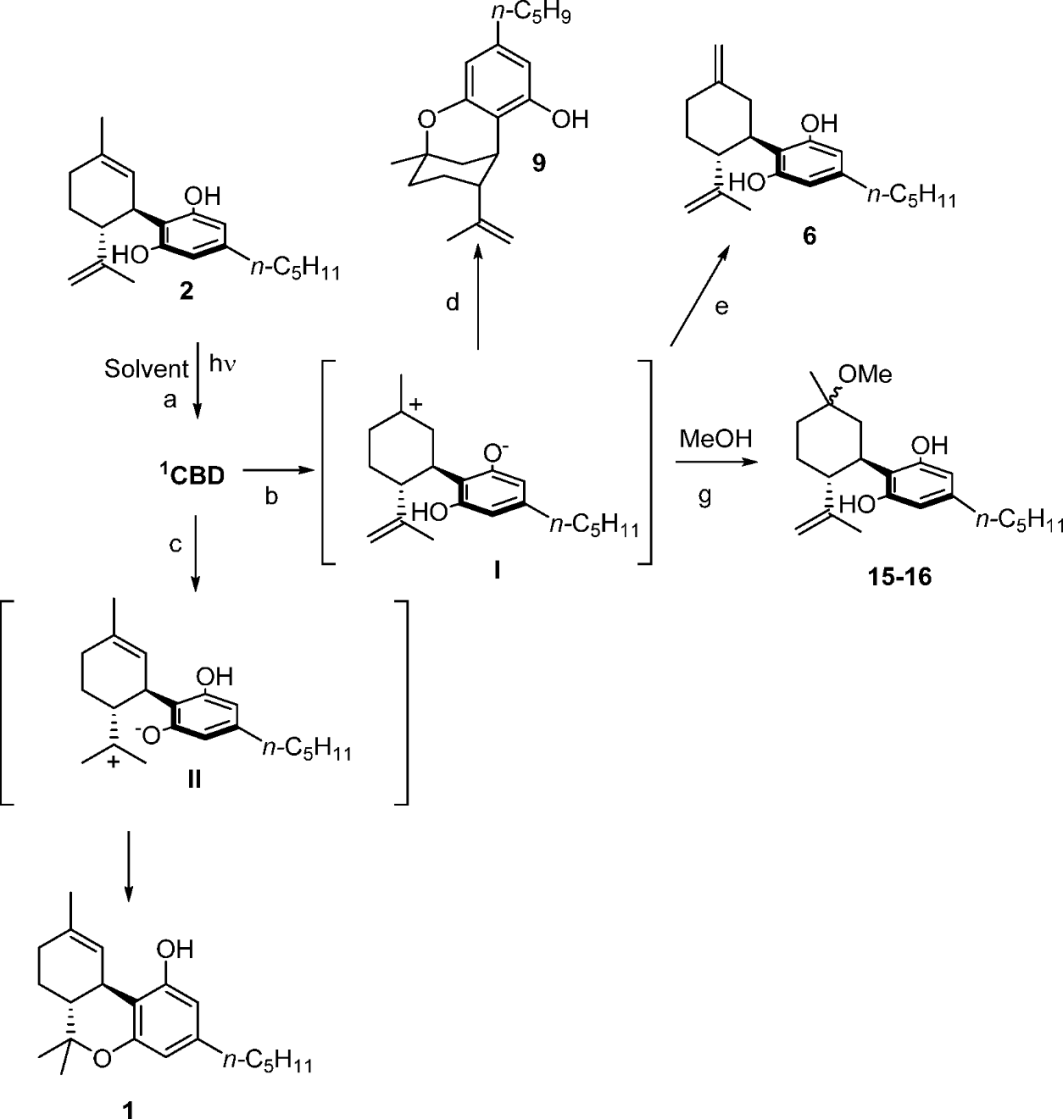
Photoreactivity of CBD (**2**) Observed in the Present Investigation

The mechanism of degradation of CBD (**2**) is described in Scheme 1. The photoacidity of phenols and the subsequent excited state proton transfer processes have been investigated in detail in the past.^17−20^ The formation of most of the photodegradation products of CBD (**2**) can be justified on the basis of an excited proton transfer process. Indeed, the resorcin moiety in cannabidiol is responsible for the absorption of light during irradiation, leading to the singlet excited state ^1^CBD (**2**) (Scheme 1, path a), which undergoes competitive excited state proton transfer (ESPT) processes to afford zwitterionic intermediates **I** and **II** (paths b and c).

The cyclization of **I** results in the formation of Δ^8^-*iso-*THC (**9**) (path d), whereas **II** acts as the precursor of Δ^9^-THC (**1**) (path e). When the irradiation is carried out in protic medium (MeOH), back proton transfer occurs in the intermediate **I** to form Δ^7^-CBD (**6**; path f; formation of this product in small amounts was observed also in MeCN and hexane), as well as trapping of the solvent, affording α,β-methoxycannabidiol (**15** and **16**, respectively; path g). Concerning the formation of low amounts of DHD (**5**) and THD (**7**), and in accordance with previous results,^12^ we suggest the occurrence of a radical mechanism in competition with the ionic paths illustrated in Scheme 1.

The irradiation of two MeCN solutions of CBD (**2**) in the absence of O_2_ and another saturated with O_2_ showed a similar consumption of the substrate and a similar distribution of products. The consumption of CBD (**2**) in the absence of O_2_ was 65%, with the formation of Δ^8^-*iso*-THC (**9**) in 30% yield and of Δ^9^- THC (**1**) in 7% yield. In the oxygenated solution we observed a CBD (**2**) consumption of 70% and formation of Δ^8^-*iso*-THC (**9**) in 35% yield and of Δ^9^- THC (**1**) in 5% yield. Irradiation at 310 nm of an argon-saturated solution of CBD (**2**) in MeCN and in 4:1 MeCN/acetone gave comparable results (see the Supporting Information, Figure 7.2). These results indicate that the role of a triplet state in the ESPT can be excluded. Furthermore, no evidence of the formation of an exciplex has been observed during the analysis of the emission spectra of CBD (**2**) in MeOH and in MeOH. In the case of *n*-hexane, due to the presence of a complex emission spectra (see the Supporting Information, Section 10) and the significant amount of dimerization products, the formation of an intermolecular exciplex cannot be excluded.

The formation of “dimerized products”, as seen by UHPLC-MS (see the Supporting Information, paragraph 8), can account for the poor mass balance observed in all cases, suggesting that oligomerization of CBD (**2**) happens as a primary reaction. The mass balance at low consumption of substrates (3 min irradiation; 10% consumption; yields estimated by GC-MS) is more satisfactory than that observed at higher irradiation times (50 min), accounting for 70–80% of the CBD (**2**) added in solution, further evidencing the photolability of primary photoproducts such as Δ^8^-*iso*-THC (**9**) and Δ^9^-THC (**1**) (see for instance Figure 2). These data illustrate that a photoinduced dimerization of CBD can occur. UHPLC-MS analysis of irradiated samples indicated the formation of two different dimeric related structures; MS/MS spectra of these compounds showed closed similarities (see the Supporting Information). A dimerization process of CBD (**2**) was also described to occur slowly under thermal conditions.^21^

### Thermal Degradation of CBD

Solutions of CBD (**2**) (10 g/L) in MeCN, MeOH, and *n*-hexane were stored at 20 °C and away from light and were analyzed by GC-MS after 4, 8, and 16 months. Results are reported in Table 2. In contrast to what was reported by Grafton,^9^ a partial thermal degradation was observed for all four solvents analyzed during the first 8 months; the most significant decrease in CBD (**2**) concentration was observed in polar/protic solvents. In all the cases, the consumption of CBD (**2**) was lower than 20%. After 16 months, extensive degradation was observed, with a reduction in the concentration of CBD (**2**) between −53% (*n*-hexane) and −77% (MeCN). None of the analyzed samples indicated the formation of degradation compounds but only the decrease of the initial CBD (**2**) concentration. Polymerization of the substrate can be the preferential fate of CBD (**2**) in these conditions, since oligomeric products were found (see the Supporting Information). UHPLC-MS analysis indicated in all solutions the presence of dimerized products related to CBD (**2**) (Supporting Information) with different *t*_R_ and fragmentation patterns when compared to dimers obtained by irradiation.

**Table 2:** Thermal Degradation (20°C) of CBD, at 10 g/L in the Described Solvents: The Residual Amount of CBD (**2**) (%) and (in Parentheses) Its Concentration Decreases

solvent	4 months	8 months	16 months
MeCN	94.3% (−5.7%)	80.2% (−19.8%)	22.2% (−77.8%)
MeOH	93.1% (−6.9%)	82.1% (−17.9%)	38.8% (−61.1%)
*n*-hexane	96.5% (−3.5%)	94.2% (−5.8%)	46.5% (−53.5%)
EtOH	92.6% (−7.4%)	86.2% (−13.8%)	37.7% (−62.3%)

In conclusion, we report the photochemistry of CBD (**2**) in hexane, MeOH, and MeCN, which we investigated in detail. Photodegradation products were fully characterized by GC-MS analyses. Among the different compounds generated upon irradiation, Δ^9^-THC (**1**) was observed in significant amounts in all of the examined conditions. The data obtained would be useful in view of evaluating the shelf stability of CBD (**2**) containing pharmaceutical and nutraceutical preparations, whose market is continuously growing.

## Experimental Section

### General Experimental Procedures

Reagents and solvents of the purest grade available were purchased from Sigma-Aldrich and used as received. Cannabidiol (>99%, pharma grade) was obtained from Fagron Italia, S.p.a. For the purification of the photoproducts, silica gel 60 Å (Sigma-Aldrich) was used as a stationary phase for column chromatography.

Cannabichromene (CBC, **3**), Δ^8^-THC (**4**), hexahydrocannabinol (HHC, **8**), Δ^8^*-iso*-THC (**9**), cannabigerol (CBG, **10**), cannabicyclol (CBL, **11**) cannabicitran (CBT, **12**), and cannabinol (CBN, **13**) were synthesized according to the literature.^22^ See the Supporting Information for details and for the optimized synthesis of 8,9-dihydrocannabidiol (DHD, **5**) and tetrahydrocannabidiol (THD, **7**).

### GC-MS Conditions and Identification of Photodegradation Products

GC-MS analyses have been performed with a Thermo Scientific DSQII single quadrupole GC/MS system (TraceDSQII mass spectrometer, Trace GC Ultra gas chromatograph, TriPlus autosampler, ThermoFisher Scientific, Waltham, MA, USA).

Chromatography was performed on a Rxi-5Sil MS capillary column (30 m length × 0.25 mm i.d. × 0.25 μm film thickness, Restek, Milan, Italy) with helium (>99.99%) as carrier gas at a constant flow rate of 1.0 mL/min. An injection volume of 1 μL was employed. The injector temperature was set at 290 °C, and it was operated in split mode (split ratio 1:10), with a split flow of 10 mL/min. The oven temperature was programmed from 130 °C (isothermal for 2 min) to 300 °C (isothermal for 5 min) at the rate of 5 °C/min. Data acquisition started 5 min after injection. The mass transfer line temperature was set at 310 °C. All mass spectra were acquired with an electron ionization system (EI) with an ionization energy of 70 eV and source temperature of 250 °C, with spectral acquisition in full scan mode, positive polarity, over a mass range of 50–950 Da with a scan rate of 735 amu/s.

For compound quantifications in the kinetic analysis, olivetol (200 mg/L) was added to the solutions as an internal standard (retention time in our conditions: 13.44 min). Quantitative analyses were done on the nonderivatized samples.

Assignment of chemical structures to chromatographic peaks was based on the comparison of their mass spectra fragmentation patterns with the pure compounds; when possible, further confirmation was done based on the databases for GC/MS NIST Mass Spectral Library (NIST 08), Wiley Registry of Mass Spectral Data (8th edition), SWGDRUG Mass Spectral Library v3.7 (2020), Cayman Spectral Library (2019) using Xcalibur MS (version 2.1), and AMDIS software. For most of the identified peaks the MS match was >80%. An orthogonal identification was performed comparing the retention index with those published (NIST). A series of *n*-alkanes (C8–C40, Aldrich, 1000 mg/L standard for GC) was used to determine the retention indices (see Supporting Information Section 2.1).

Peaks accounting for <2% TIC were not taken into account, unless proper standards were available for their identification.

### Derivatization of Standards and Samples for GC-MS Analysis

With the aim of double checking the identification of the compounds and reporting fragmentation data and retention indices for the compounds whose data were absent in literature, samples and standards have been analyzed both underivatized and derivatized as trimethylsilyl ethers. The derivatization of samples has been carried out by standard procedures.^23−26^ An appropriate volume of standard or unknown solution, depending on the desired final concentration, is placed in 1.5 mL Pyrex vials sealed with porous septa plugs. The sample is then brought to dryness by evaporation of the solvent with nitrogen flow. 50 μL of EtOAC and 50 μL of derivatizing agent (*N*,*O*-bis(trimethylsilyl)trifluoroacetamide with 1% trimethylchlorosilane, BSTFA-TMCS, 99:1 derivatizing agent for GC, TCI Chemical Industries, Tokyo) are added to the sample. The closed vial is then placed in a preheated oven at 70 °C for 30 min; subsequently the solution is brought to a volume of 1 mL with EtOAc and the solution analyzed.

### Photophysics and Photochemistry of CBD

UV–vis absorption spectra have been measured by means of a Jasco V-550 dual beam instrument. Emission spectra were measured by using a PerkinElmer LS55 spectrofluorometer. A scan from 200 to 800 nm was performed with solutions at a concentration of 1 × 10^–4^ M in hexane, MeCN, and MeOH to calculate the ε at the maximum of absorption.

The instrument was set with an excitation wavelength at 273 nm. The samples were analyzed at a concentration of 10^–4^ M in the three solvents. Quantum yield consumption (Φ_–1_) values for CBD (**2**) in the examined solvents have been calculated at 254 nm (2 × 15 W low-pressure Hg lamps) by using 4-chloroanisole as the reference (Φ_–2_ = 0.10 in MeOH).^16^ The photodegradation kinetics of CBD (**2**) was investigated on a argon-saturated 1.27 × 10^–3^ M CBD (**2**) solution in the chosen solvent. Experiments were performed in quartz tubes, and irradiations were carried out in a Rayonet photochemical reactor equipped with 10 × 15 W low-pressure Hg lamps (λ_em_ = 254 nm). The temperature inside the photoreactor was 20 °C.

The thermal stability of CBD (**2**) was tested by keeping 10 g/L solutions of the starting substrate in *n*-hexane, MeCN, and MeOH. The solutions were analyzed by GC-MS after 4, 8, and 16 months.

### Irradiation of CBD (**2**) in MeOH on a Preparative Scale: Separation and Identification of Δ^8^-*iso*-THC (**9**), Δ^7^-CBD (**6**), α,β-MeO (**15**, **16**), and MeO-CBE (**14**)

A 200 mg (0.64 mmol) amount of CBD (**2**) in 100 mL of MeOH was irradiated in a quartz tube in the Rayonet for 2 h. The consumption of the starting substrate was followed with GC-MS. The irradiated solution was then evaporated *in vacuo*, and the residue purified by column chromatography (eluent: cyclohexane/EtOAc, 99:1) allowed for the isolation of MeO-CBE (**14**, 8.5 mg, 0.024 mmol) and α,β-MeO (**15**, **16**, 15.5 mg, 0.045 mmol); 120 mg of unreacted CBD (**2**) was recovered.

To increase the consumption of CBD (**2**), the same amount of CBD (**2**) was irradiated in a quartz tube in the Rayonet for 4 h. The irradiated solution was then evaporated *in vacuo*, and the residue purified by column chromatography (eluant: cyclohexane/EtOAc, 99:1) to afford six fractions, from which Δ^8^-*iso*-THC (**9**, 21.9 mg, 0.07 mmol) and Δ^7^-CBD (**6**, 13.2 mg, 0.042 mmol) were isolated and 68.1 mg of unreacted CBD (**2**) was recovered.

**Δ**^**7**^**-CBD (6):**^1^H NMR (300 MHz, acetone-*d*_6_) δ 6.10 (m, 2H), 4.70 (m, 4H), 4.50 (s, 1H), 3.2 (m, 2H), 2.8 (m, 1H), 2.45 (m, 3H), 1.60 (m, 6H), 1.40 (m, 6H), 0.9 (t, J = 6.5 Hz, 3H); ^13^C NMR (75 MHz, acetone-*d*_6_) δ 149.4, 149.3, 148.0, 120.6, 114.6, 110.5, 110.1, 109.5, 109.0, 108.1, 107.9, 107.0, 77.3, 77.1, 76.9, 76.5, 47.29, 44.5, 39.8, 38.7, 35.2, 35.1, 34.9, 34.5, 33.9, 33.1, 32.2, 31.5, 31.3, 30.4, 29.6, 28.0, 27.0, 22.4, 22.2, 19.4, 13.9, 13.8.

**α,β-MeO-CBD (15, 16):**^1^H NMR (400 MHz, CDCl_3_) δ 6.10 (dd, *J* = 19.8, 1.5 Hz, 2H), 4.69 (d, *J* = 2.5 Hz, 1H), 4.53 (dd, *J* = 2.6, 1.4 Hz, 1H), 3.31–3.24 (m, 3H), 3.03 (td, *J* = 11.8, 3.4 Hz, 1H), 2.47–2.28 (m, 3H), 1.91–1.65 (m, 4H), 1.54 (ddd, *J* = 12.2, 10.5, 3.7 Hz, 4H), 1.42–1.19 (m, 12H), 0.90 (t, *J* = 7.0 Hz, 3H); ^13^C NMR (101 MHz, CDCl_3_) δ 156.0, 153.7, 149.4, 142.0, 114.7, 109.7, 109.1, 107.7, 75.9, 48.4, 47.3, 39.8, 36.7, 35.3, 35.2, 31.6, 30.5, 29.9, 22.5, 20.8, 19.5, 14.0.

**MeO-CBE (14):**^1^H NMR (400 MHz, CDCl_3_) δ 7.54 (s, 1H), 7.02 (s, 1H), 6.20 (s, 1H), 6.08 (s, 1H), 4.70 (s, 1H), 4.54 (m, 2H), 3.40 (dd, J = 11.7, 8.7 Hz, 2H), 3.26 (s, 3H), 2.92 (m, 2H), 2.46–2.39 (m, 3H), 2.07–1.70 (m, 6H), 1.45–1.17 (m, 8H), 0.90 (t, J = 6.8 Hz, 3H).

**Δ**^**8**^**-*****iso*****-THC (9):**^1^H NMR (300 MHz, CDCl_3_) δ 6.31 (d, *J* = 1.5 Hz, 1H), 6.14 (d, *J* = 1.5 Hz, 1H), 5.08–4.86 (m, 2H), 3.49 (q, *J* = 3.0 Hz, 1H), 2.47 (dd, *J* = 8.9, 6.7 Hz, 2H), 2.36 (d, *J* = 4.5 Hz, 1H), 1.89 (d, *J* = 9.1 Hz, 4H), 1.82–1.45 (m, 7H), 1.34 (d, *J* = 6.0 Hz, 8H), 0.93 (t, *J* = 6.9, 3H); ^13^C NMR (75 MHz, CDCl_3_) δ 157.3, 152.1, 146.0, 142.5, 110.9, 110.7, 107.8, 105.9, 74.6, 42.9, 35.6, 35.3, 31.5, 30.7, 30.4, 29.3, 27.8, 22.6, 22.4, 21.0, 13.9.

## Supporting Information Available

General procedures, copy of NMR (^1^H and ^13^C both), and MS plots (PDF)

## References

[ref1] De Meijer E. P. M.; Hammond K. M. Euphytica 2005, 145, 189–198. 10.1007/s10681-005-1164-8.

[ref2] Pollastro F.; Minassi A.; Fresu L. G. Curr. Med. Chem. 2018, 25, 1160–1185. 10.2174/0929867324666170810164636. 28799497

[ref3] Russo E. B. Br. J. Pharmacol. 2011, 163, 1344–1364. 10.1111/j.1476-5381.2011.01238.x. 21749363 PMC3165946

[ref4] Burstein S. Bioorg. Med. Chem. 2015, 23, 1377–1385. 10.1016/j.bmc.2015.01.059. 25703248

[ref5] Pertwee R. G. Br. J. Pharmacol. 2008, 153, 199–215. 10.1038/sj.bjp.0707442. 17828291 PMC2219532

[ref6] Devinky O.; Cilio M. R.; Cross H.; Fernandez-Ruiz J.; French J.; Hill C.; Katz R.; Di Marzo V.; Jutras-Asward D.; Notcutt W. J.; Martinez-Orgado J.; Robson P.; Rohrback B.; Thiele E.; Whalley B.; Friedman D. Epilepsia 2014, 55, 791–802. 10.1111/epi.12631. 24854329 PMC4707667

[ref7] Iuvone T.; Esposito G.; De Filippis D.; Scuderi C.; Steardo L. CNS Neurosci. Ther. 2009, 15, 65–75. 10.1111/j.1755-5949.2008.00065.x. 19228180 PMC6494021

[ref8] Golombek P.; Müller M.; Barthlott I.; Sproll C.; Lachenmeier D. W. Toxics 2020, 8, 41 10.3390/toxics8020041. PMC7357058 32503116

[ref9] Graftrom K.; Andresson K.; Pettersson N.; Dalggad J.; Dunne S. J. Forensic Sci. Int. 2019, 301, 331–340. 10.1016/j.forsciint.2019.05.035. 31202146

[ref10] Lydon A. H.; Teramura J. Phytochemistry 1987, 26, 1216–1217. 10.1016/S0031-9422(00)82388-2.

[ref11] Loewe S. Naunyn-Schmiedeberg's Arch. Pharmacol. 1950, 211, 175–193. 10.1007/BF00249872. 14777528

[ref12] Shani R.; Mechoulam A. Tetrahedron 1971, 27, 601–606. 10.1016/S0040-4020(01)90728-8.

[ref13] Allward W. H.; Babcock P. A.; Segelman A. B.; Cross J. M. J. Pharm. Sci. 1994, 64, 1994–1996. 10.1002/jps.2600611225.

[ref14] Lydon A. H.; Teramura J. Phytochemistry 1987, 26, 1216–1217. 10.1016/S0031-9422(00)82388-2.

[ref15] Jiménez M. C.; Miranda M. A.; Tormos R. Chem. Soc. Rev. 2005, 34, 783–796. 10.1039/b416410p. 16100618

[ref16] Dichiarante V.; Dondi D.; Protti S.; Fagnoni M.; Albini A. J. Am. Chem. Soc. 2007, 129, 5605–5611. 10.1021/ja068647s. 17407290

[ref17] Crombie L.; Ponsford R.; Shani A.; Yanitinky B.; Mechulam R. Tetrahedron Lett. 1968, 9, 5771–5772. 10.1016/S0040-4039(00)76346-5. 5697175

[ref18] Domcke W.; Sobolewski A. L. Science 2003, 302, 1693–1694. 10.1126/science.1093081. 14657482

[ref19] Wang W.-F.; Cheng Y.-C. Phys. Chem. Chem. Phys. 2018, 20, 4351–4359. 10.1039/C7CP01948C. 29367985

[ref20] da Silva L. P.; Green O.; Gajst O.; Simkovitch R.; Shabat D.; Esteves da Silva J. C. G.; Huppert D. ACS Omega 2018, 3, 2058–2073. 10.1021/acsomega.7b01888. 31458515 PMC6641337

[ref21] Mechoulam R.; Hanus L. Chem. Phys. Lipids 2002, 121, 35–43. 10.1016/S0009-3084(02)00144-5. 12505688

[ref22] Yeom H.-S.; Li H.; Tang Y.; Hsung R. P. Org. Lett. 2013, 15, 3130–3133. 10.1021/ol401335u. 23730958

[ref23] Villamor J. L.; Bermejo A. M.; Tabernero M. J.; Fernandez P. Anal. Lett. 2004, 37, 517–528. 10.1081/AL-120028624.

[ref24] De Brabanter N.; Van Gansbeke W.; Hooge F.; Van Eenoo P. Forensic Sci. Int. 2013, 224, 90–95. 10.1016/j.forsciint.2012.11.004. 23206547

[ref25] Foltz R. L. Mass Spectrometric Methods for Determination of Cannabinoids in Physiological Specimens. In Forensic Science and Medicine: Marijuana and the Cannabinoids; El Sohly M. A., Ed.; Humana Press Inc.: Totowa, NJ, 2007.

[ref26] Nadulski T.; Sporkert F.; Schnelle M.; Stadelman M. A.; Roser P.; Schefter T.; Pragst F. J. Anal. Toxicol. 2005, 29, 782–789. 10.1093/jat/29.8.782. 16356335

