# Supplementary material for: Photochemistry of Cannabidiol (CBD) Revised. A Combined Preparative and Spectrometric Investigation
Source: J Nat Prod. 2021 Oct 20;84(11):2858–65. doi: 10.1021/acs.jnatprod.1c00567 (PMC8765678; doi:10.1021/acs.jnatprod.1c00567)
Supplement: Supplementary file 1 — np1c00567_si_001.pdf [file np1c00567_si_001.pdf]

Supplementary Information

Photochemistry of Cannabidiol (CBD) revised. A combined preparative and spectrometric investigation

Paolo Seccamani,[a,§] Chiara Franco,[a,§] Stefano Protti,[a] Alessio Porta,[a] Antonella Profumo,[a] Diego Caprioglio,[c] Stefano Salamone,[c] Barbara Mannucci,[d] Daniele Merli [a,b*]

[a] *Dipartimento di Chimica, Università degli Studi di Pavia, 27100 Pavia, Italy.* [b] *INFN Sezione di Milano-Bicocca, Piazza della Scienza 3, 20126 Milano, Italy.* [c] *Department of Pharmaceutical Sciences, University of Piemonte Orientale, Novara, Italy.* [d] *Centro Grandi Strumenti, Università degli Studi di Pavia, 27100 Pavia, Italy.* [§] These authors contributed equally to the work

*E-mail address: daniele.merli@unipv.it.

Table of contents.

1. List of abbreviations S2

2. Experimental details S3

3. Synthesis of the cannabinoids used as standards S7

4. Mass spectra of reported compounds – underivatized compounds S16

5. Mass spectra of reported compounds - derivatized compounds S31

6. ^{1}H and ^{13}C spectra of representative compounds S41

7. Irradiation at 310 nm S56

8. Formation of dimeric products: UHPLC-HES-MS analysis S59

9. Kinetic data for the degradation of CBD in the examined solvents S66

10. Emission spectra of CBD in the examined solvents **S67**

11. References **S71**

1. List of abbreviations

CBD: cannabidiol (**2**)

Δ^9-THC: delta-9-tetrahydrocannabinol (**1**)

Δ^8-THC: delta-8-tetrahydrocannabinol (**4**)

Δ^8-*iso*-THC: delta-8-iso-tetrahydrocannabinol (**9**)

CBC: cannabichromene (**3**)

CBL: cannabicyclol (**11**)

CBG: cannabigerol (**10**)

CBN: cannabinol (**13**)

CBT: cannabicitran (**12**)

HHC: hexahydrocannabinol (**8**)

DHD: 8,9-dihydrocannabidiol (**5**)

THD: tetrahydrocannabidiol (**7**)

Δ^7-CBD: delta-7-cannabidiol (**6**)

MeO-CBE: methoxy-dehydrocannabielsoin (**14**)

α-MeO: α-methoxy-dihydrocannabidiol (**15**)

β-MeO: β-methoxy-dihydrocannabidiol (**16**)

2. Experimental section

2.1. General details

Reagents and solvents of the purest grade available were purchased from Sigma-Aldrich and used as received. Cannabidiol (CBD, >99%, pharma grade) was obtained from Fagron Italia, S. p. a. UV-VIS absorption spectra have been measured by means of a Jasco V-550 dual beam instrument. Emission spectra were measured by using a Perkin Elmer LS55 spectrofluorometer.

GC-MS analyses have been performed with a Thermo Scientific DSQII single quadrupole GC/MS system (TraceDSQII mass spectrometer, Trace GC Ultra gas chromatograph, TriPlus autosampler - ThermoFisher Scientific, Waltham, MA, USA).

Chromatography was performed on a Rxi-5Sil MS capillary column (30 m length x 0.25 mm ID x 0.25 µm film thickness, Restek, Milan, Italy) with helium (>99.99 %) as carrier gas at a constant flow rate of 1.0 mL/min. An injection volume of 1 µL was employed. The injector temperature was set at 290°C and it was operated in split mode (split ratio 1:10), with a split flow of 10 mL/min. The oven temperature was programmed from 130°C (isothermal for 2 min) to 300°C (isothermal for 5 min) at the rate of 5 °C/min. Data acquisition started 5 minutes after injection. Mass transfer line temperature was set at 310 °C. All mass spectra were acquired with an electron ionization system (EI, Electron Impact mode) with ionization energy of 70 eV and source temperature of 250°C, with spectral acquisition in Full Scan mode, positive polarity, over a mass range of 50–950 Da with a scan rate of 735 amu/s.

For compounds quantifications in the kinetic analysis, olivetol (200 mg/L) was added to the solutions as internal standard (retention time in our conditions: 13.44 min). Quantitative analyses were done on the non-derivatized samples.

Assignment of chemical structures to chromatographic peaks was based on the comparison of their mass spectra fragmentation patterns with the pure compounds; when possible, further confirmation was done basing on the databases for GC/MS NIST Mass Spectral Library (NIST 08), Wiley Registry of Mass Spectral Data (8th Edition), SWGDRUG Mass Spectral Library v3.7 (2020), Cayman

Spectral Library (2019) using Xcalibur MS (Version 2.1) and AMDIS software. For most of the identified peaks the MS match was >80%. An orthogonal identification was performed comparing the retention index with those published (NIST). A series of n-alkanes (C8-C40, Aldrich, 1000 mg/L standard for GC) was used to determine the retention indices.

Retention indices were calculated according to Eq. (1).

$$I = \left[\frac{tr\ (unknown) - tr\ (n)}{tr\ (N) - tr\ (n)}\right]\cdot(100\cdot z) + (100\cdot a) \qquad (1)$$

Where t_r is the retention time, *n* and *N* respectively the alkanes retained before and after the considered compound, *z* is the difference in number of carbons between *N* and *n*, and *a* is the number of carbons of the lighter alkane.

The ionization efficiency and the MS response were periodically checked (i.e. weekly) by analysing a reference standard solution of PHA at known concentration (Polycyclic Aromatic Hydrocarbon Standard, certified reference material, 1000 µg/mL in methylene chloride, Aldrich, code UST122 diluted at 1 µg/mL) Calibration curve for the quantification of CBD and related compounds was performed using solutions with a known increasing concentration of analyte and measuring the areas of the peaks obtained (Total Ion Current, TIC).

For compounds quantifications in the kinetic analysis, olivetol (200 mg/L) was added to the solutions as internal standard.

Standards of the compounds used for the GC-MS analysis were prepared weekly by dissolving the proper amount of compound in ethyl acetate to a 1000 µg/mL final concentration and diluting opportunely for the analysis. The concentrations of each standard in ethyl acetate used for the calibration were 1 mg/L, 2.5 mg/L, 6 mg/L, 15 mg/L, 40 mg/L. The preparative HPLC purification and analyses have been performed with an Agilent 1260 system instrument equipped with DiodeArray as a detector. The column used for purification was C-18 150 x 30 mm Waters CSH,

particle size 5 µm. Mobile phases that were used are eluent A: water containing 0.2% TFA, eluent B: acetonitrile containing 0.2% of TFA. Gradient used for separation: from 90:10 A/B to 100% B in 20 min, flow rate 30 mL/min.

Derivatization of standards and samples.

With the aim of double checking the identification of the compounds and reporting fragmentation data and retention index for the compounds whose data were absent in literature, samples and standards have been analysed both underivatized and derivatized as trimethylsilyl ethers. The derivatization of samples has been carried out by following a known procedure.[S1-S4] An opportune volume of standard or unknown solution, depending on the desired final concentration, is placed in 1.5 mL Pyrex vials sealed with porous septa plugs. The sample is then brought to dryness by evaporation of the solvent with nitrogen flow. 50 µL of ethyl acetate and 50 µL of derivatizing agent (N,O-Bis(trimethylsilyl)trifluoroacetamide with 1% trimethylchlorosilane, BSTFA-TMCS, 99:1 derivatizing agent for GC, TCI Chemicals) are added to the sample. The closed vial is then placed in a preheated oven at 70 °C for 30 minutes, subsequently the solution is brought to a volume of 1 mL with ethyl acetate.

Table 2.1. Retention index of the studied compounds and of their trimethylsilyl (TMS) derivatives. The meaning of the abbreviations is reported after the Conclusion chapter. N.A: not available in literature. [a]data from NIST library.

**Compound**	**Retention time (min)**	**Retention index (found)**	**Retention index (literature)[a]**
CBD (**2**)	25.50	2416	2393, 2383, 2385, 2366
Δ^9-THC (**1**)	26.89	2467	2466
CBC (**3**)	25.71	2429	2417
CBL (**11**)	24.75	2368	N.A.
CBG (**10**)	27.84	2528	2496
CBN (**13**)	28.69	2584	2538, 2495, 2525, 2612
cis-HHC (*cis*-**8**)	26.06	2449	2464
trans-HHC (*trans*-**8**)	26.20	2458	2464
DHD (**5**)	25.98	2444	N.A.
CBT (**12**)	23.28	2276	N.A.
Δ^8-THC (**4**)	26.73	2491	2475
Δ^8-iso-THC (**9**)	25.77	2431	N.A.
Δ^7-CBD (**6**)	26.50	2477	N.A.
MeO-CBE (**14**)	28.00	2571	N.A.
α-MeO (**15**)	28.35	2593	N.A.
β-MeO (**16**)	29.15	2643	N.A.
*cis-THD (cis-***7***)*	26.35	2468	2563
trans-THD (*trans*-**7**)	26.48	2476	2563
CBD-2TMS	22.95	2229	2760
Δ^9-THC-TMS	24.80	2338	2552
CBC-2TMS	24.18	2306	N.A.
CBL-TMS	23.53	2263	N.A.
CBG-2TMS	25.72	2394	N.A.
CBN-TMS	26.13	2419	2659
cis-HHC- TMS	23.92	2316	N.A.
trans-HHC- TMS	24.92	2378	N.A.
DHD- 2TMS	22.84	2248	N.A.
Δ^8-THC- 2TMS	24.50	2352	2552
iso-THC-2TMS	22.96	2256	N.A.
cis-THD-TMS	22.23	2210	2722
trans-THD-TMS	23.42	2284	2722
Δ^7-CBD-2TMS	22.57	2231	N.A.
MeO-CBE-2TMS	24.40	2346	N.A.
α-MeO-2TMS	24.38	2344	N.A.
β-MeO-TMS	25.26	2399	N.A.

3. Synthesis of the cannabinoids used as standards

3.1. Oxidation of geraniol to geranial

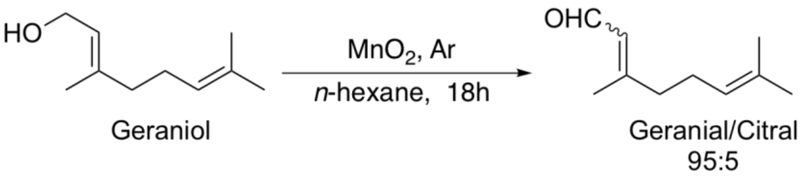

In a round bottom flask with an anhydrous neck and under an Ar static atmosphere, geraniol (3.4 g, 21.9 mmol) was solubilized in 220 mL dry hexane. Subsequently, MnO_2 (34 g, 10 equiv.) was added, in a single portion, to the geraniol solution forming a dark slurry. The reaction mixture was left under magnetic stirring at room temperature for about 12 hours, at the end of which an incomplete conversion was observed (the progress of the reaction was monitored by TLC). To complete the reaction, the mixture was heated at 40 °C for about 3h, previously adding a smooth-bore coolant. After 3h, the almost complete conversion of the reagent into the products was observed. The reaction was allowed to cool to room temperature, and the reaction mixture, previously diluted with dichloromethane (DCM), was filtered on a Celite pad to remove the MnO_2 present, washing everything with abundant DCM. The transparent liquid thus obtained was dried on Na_2SO_4 for 15 min, filtered on a folded filter and concentrated under reduced pressure, until a slightly yellow oil was obtained. The crude product was then purified by direct phase silica gel chromatographic column using as eluent Hex: EtOAc 8:2 (the ratio between product and silica was 1:40). After purification, 2.53 g (with a yield of 75%) of the product were obtained, as an *E*/*Z* mixture (95:5 *E*/*Z*, GC-MS) of the desired aldehyde as a mixture of geranial and citral. Spectroscopic data are in accordance with the literature.[S5]

Geranial. ^{1}H NMR (300 MHz, $CDCl_3$) δ: 10.0 (d, *J* = 10 Hz, 1H), 5.95 (d, *J* = 10 Hz, 1H), 5.23 (t, *J* = 7 Hz, 1H), 2.37 (t, *J* = 7 Hz, 2H) 2.27 (m, 2H), 1.96 (s, 3H), 1.57 (s, 6H). GC-MS (EI, 70 cV), m/z (%): 152 (4%) [M^+], 123 (10%), 84 (20%), 69 (100%).

3.2. Cyclization of geranial to cannabichromene (CBC, 3) and cannabicyclol (CBL, 11)

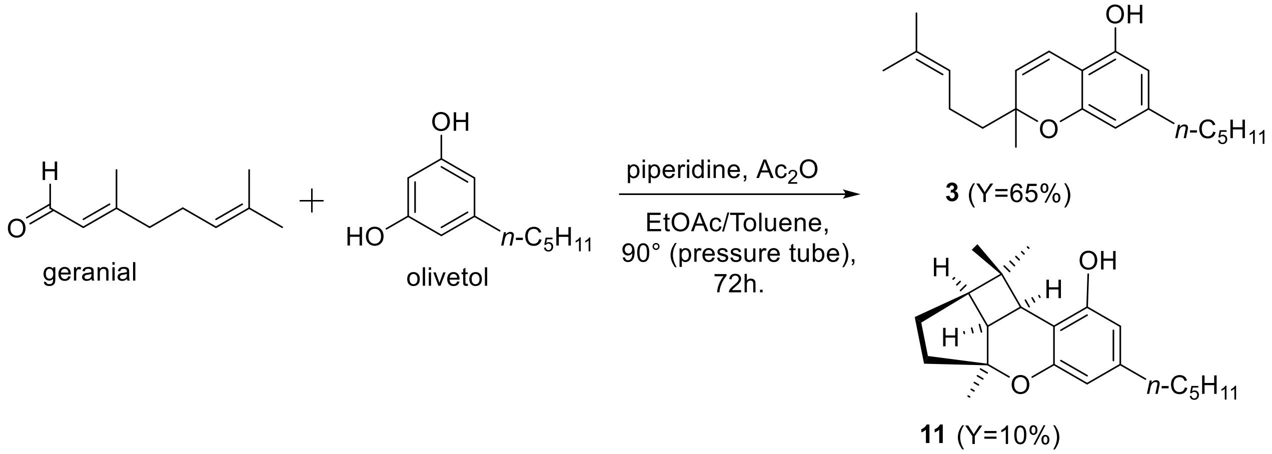

The procedure described in literature [S6, S7] was used. To solution of geranial (224 µL, 1.29 mmol) and piperidine (0.28 mL, 2.85 mmol) in EtOAc (2.9 mL), Ac_2O (0.28 mL, 2.98 mmol) was added dropwise at 0 °C. After addition, the flask was sealed and heated in a 90 °C oil bath for 1 h. The resulting iminium salt solution was then added to a solution of olivetol (0.35 g, 1.95 mmol) in toluene (5.5 mL) dropwise through cannula at room temperature under Ar atmosphere. The mixture was thus stirred at 130 °C for 40 h. Then, the reaction mixture was cooled to room temperature and quenched with satured aq. solution of $NaHCO_3$, and extracted with EtOAc. The combined organic layers were washed with saturated aq. NaCl, dried over anhyd Na_2SO_4, and concentrated *in vacuo*. The crude product was then purified by direct phase chromatographic column, using as eluent Hex: EtOAc 9:1 (stationary phase silica, the ratio between product and silica was 1:40), performed twice to resolve the mixture of products. 263.2 mg of cannabichromene (CBC, **3**, yield: 65%) and 40.5 mg of cannabicyclol (CBL, **11**, yield: 10%) were isolated. The obtained products were characterized in ^{1}H NMR and ^{13}C NMR and GC-MS (see below). Spectroscopic data are in accordance with the literature.[S6,S7]

Cannabichromene (CBC, 3). ^{1}H NMR (300 MHz, $CDCl_3$) δ: 6.68 (d, J = 10 Hz, 1H), 6.28 (m, 1H), 6.12 (s, 1H), 5.5 (d, J = 10 Hz, 1H), 5.12 (t, J= 7 Hz, 1H), 4.68 (s, 1H), 2,45 (t, J = 7.5 Hz, 2H), 2.15-2 (m, 3H), 1.75-1.5 (m, 9H), 1.38 (s, 2H), 0.90 (t, J = 7 Hz, 3H). **^{13}C NMR** (75 MHz, $CDCl_3$) δ:

153.9, 150.8, 144.6, 131.5, 127.1, 124.1, 116.6, 109.0, 107.5, 106.8, 40.9, 37.8, 31.5, 30.5, 26.1, 25.6, 22.6, 22.4, 17.5, 14.5, 13.9.

Cannabicyclol (CBL, 11). ^{1}**H NMR** (300 MHz, $CDCl_3$) δ: 6.33 (d, J= 13 Hz, 2H), 2.92 (s, 1H), 2.57 (t, *J* = 7.8 Hz, 2H), 2.34 (dt, *J* = 3 Hz, 2H), 2.12 (m, 2H), 1.81 (dd, J= 14 Hz, 3 Hz, 2H) 1.60 (t, *J* = 6.5 Hz, 5H) 1.58 (s, 3H), 1.34 (s, 3H), 1.33-1.25 (m, 8H), 1.05 (s, 3H), 0.94 (t, *J* = 7 Hz, 4H), 0.6-0.75 (m, 1H). ^{13}**C NMR** (75 MHz, $CDCl_3$) δ: 157.8, 156.4, 142.4, 113.9, 109.6, 108,8, 83.4, 74,3, 46.7, 37.2, 36.0, 35.3, 31.3, 30.8, 29.6, 29.6, 29.0, 23.6, 22.4, 22.0, 13.9.

3.3. Synthesis of cannabinol (CBN, 13).

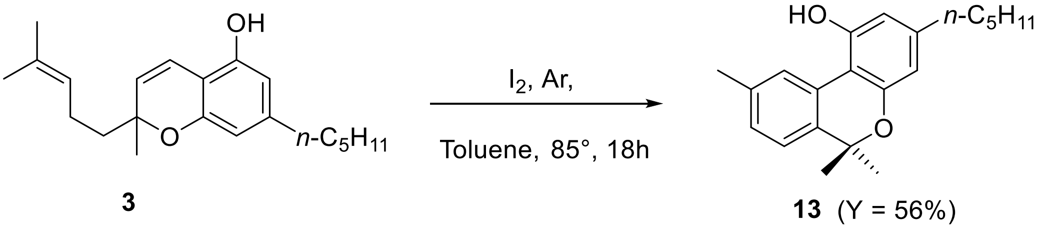

The procedure described in literature[S8] was followed. 240.9 mg of CBC (**3**, 0.77 mmol) was dissolved in dry toluene (15.5 mL) under an Ar atmosphere, molecular iodine (390.9 mg, 1.54 mmol, 2 equiv.) was then added and the reaction mixture was heated at 85 °C for 18h. The reaction was quenched with 5% aqueous solution of $Na_2S_2O_3$, organic layers were dried over $MgSO_4$, filtered and concentrated under reduced pressure. The crude product was then purified by silica gel column (product ratio: silica about 1:40) with an eluent mixture Hex: EtOAct = 8:2. 133.2 mg of cannabinol (CBN, **13**) were obtained; yield 56%. The product obtained was characterized by GC-MS (see below) and with ^{1}H NMR and ^{13}C NMR. Spectroscopic data are in accordance with the literature.[S8]

Cannabinol (CBN, 13). ^{1}**H NMR** (300 MHz, $CDCl_3$) δ: 8.12 (d, *J* = 1.4 Hz, 1H), 7.23 (d, *J* = 8 Hz, 1H) 7.17 (d, *J* = 8 Hz, 1H), 6.94 (d, 1H), 6.69 (d, 1H), 2.44 (t, *J* = 7.4 Hz, 2H), 2.41 (s, 3H), 2.31 (s,

3H), 1.61 (m, 2H), 1.32-1.25 (s, 8H), 1.25 (m, 2H), 0.87 (t, J = 6.5 Hz, 3H). **^{13}C NMR** (75 MHz, $CDCl_3$) δ: 154.8, 154.7, 142.7, 138.0, 132.0, 123.8, 121.8, 110.7, 108.4, 108.3, 39.7, 35.5, 31.5, 30.8, 26.4, 25.6, 22.5, 22.2, 17.6, 16.1, 14.0.

3.4. Synthesis of cannabigerol (CBG, 10)

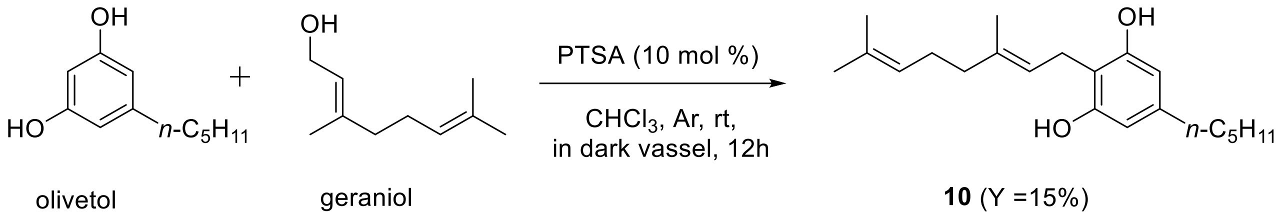

For the synthesis of cannabigerol (CBG, **10**) a literature procedure was followed.[S9] In a dark glass round bottom flask, olivetol (990 mg, 5.5 mmol) was dissolved in dry DCM (183 mL). Geraniol, (1.67 mL, 9.63 mmol, 1.75 equiv.) was then added followed by PTSA (94.6 mg, 10 mol %). The reaction mixture was stirred at RT for 12h and then quenched with 5% aq. $NaHCO_3$, organic layer was collected and aqueous phase was extracted with DCM (3x70 mL). Combined organic phases were dried over $MgSO_4$, filtered and volatiles were removed under reduced pressure. The oil thus obtained was purified by silica gel chromatographic column (ratio of silica produced approximately 1:35), using the eluent mixture Hex: MTBE 9:1. 27 mg of cannabigerol (**10**) were isolated with a 15% yield. The product obtained was characterized by GC-MS (see below) and with ^{1}H NMR and ^{13}C NMR. Spectroscopic data are in accordance with the literature.[S9]

Cannabigerol (CBG, 10). ^{1}H NMR (300 MHz, $CDCl_3$) δ: 6.78 (s, 2H), 5.13 (t, J = 6.3 Hz, 1H), 5.04 (t, J = 8 Hz, 2H), 3.28 (d, J= 7 Hz, 2H) 2.56 (t, J = 7.5 Hz, 2H) 2.21 (m, 1H), 2.26-2.17 (s, 8H), 2.11 (t, J = 7 Hz, 2H), 1.56-1.50 (m, 11H) 1.2 (m, 4H), 0.87 (t, J = 7 Hz, 3H). **^{13}C NMR** (75 MHz, $CDCl_3$) δ: 154.8, 142.6, 138.9, 131.9, 123.6, 121.6, 110.4, 108.2, 39.6, 35.4, 31.4, 30.9, 30.7, 26.3, 25.6, 22.4, 22.1, 17.6, 13.9.

3.5. Synthesis of Δ^{8}-tetrahydrocannabinol (Δ^{8}-THC, 4)

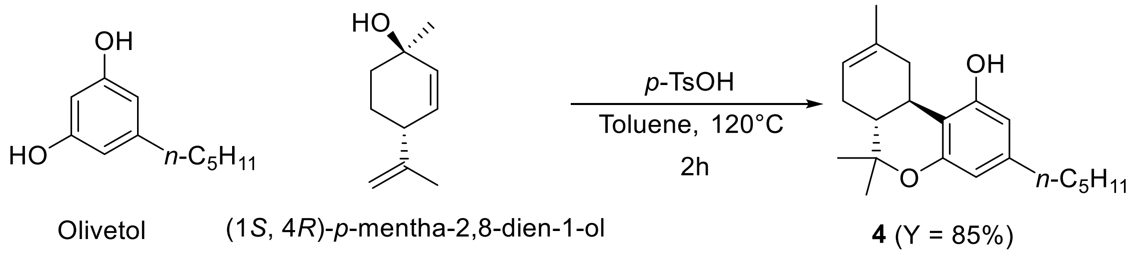

The procedure described in literature was used.[S4] To a stirred solution of olivetol (100 mg, 0.56 mmol, 1 eq) and (1*S*, 4*R*)-*p*-mentha-2,8-dien-1-ol (92 mg, 0.61 mmol, 1.1 eq) in toluene (2 mL), monohydrate *p*-toluenesulfonic acid (PTSA, 20 mg, 0.11 mmol, 0.2 eq) was added. The solution was heated at 120 °C for 2 hours, then cooled to room temperature and quenched with saturated NaCl. The organic phase was extracted with EtOAc, dried over Na_2SO_4 and concentrated under reduced pressure. Purification by flash column chromatography on silica gel (petroleum ether, PE, 100% to PE:DCM 8:2 as eluent) gave Δ^8-THC (**4**, 149 mg, 85%) as a brown oil. The afforded compound showed spectral properties identical to those reported in the literature. Spectroscopic data are in accordance with the literature.[S8]

Δ^8-tetrahydrocannabinol (Δ^8-THC, 4). ^{1}H NMR (400 MHz, $CDCl_3$): δ = 6.29 (d, *J* = 1.2 Hz, 1 H), 6.12 (d, *J* = 1.2 Hz, 1 H), 5.44 (d, *J* = 4.4 Hz, 1 H), 4.68 (s, 1 H), 3.20 (dd, *J* = 16.0, 4.0 Hz, 1 H), 2.71 (td, *J* = 10.8, 4.8 Hz, 1 H), 2.43–2.47 (m, 2 H), 2.14–2.19 (m, 1 H), 1.80–1.84 (m, 3 H), 1.67 (s, 3 H), 1.55–1.58 (m, 2 H), 1.39 (s, 3 H), 1.27–1.33 (m, 4 H), 1.12 (s, 3 H), 0.87–0.91 (m, 3 H). **^{13}C NMR** (100 MHz, $CDCl_3$): δ = 154.8, 154.7, 142.7, 134.7, 119.3, 110.8, 110.1, 107.6, 76.6, 44.9, 36.0, 35.4, 31.6, 30.6, 27.9, 27.6, 23.5, 22.5, 18.5, 14.0.

3.6. Optimization of the synthesis of 8,9-dihydrocannabidiol (DHD, 5) and tetrahydrocannabidiol (THD, 7)

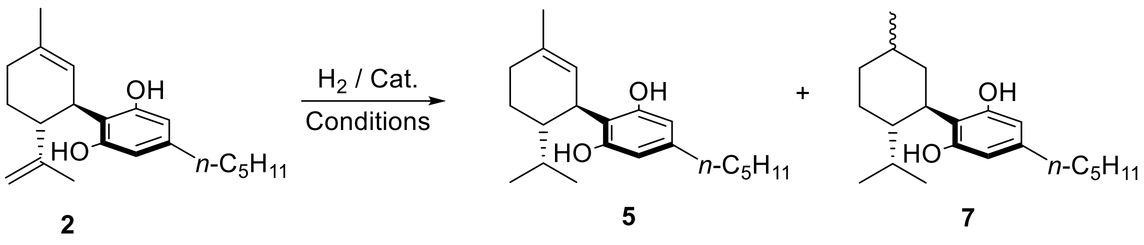

CBD (**2**, 30 mg, 0.096 mmol) was dissolved in a mixture of hexanes:EtOAc (from 4:1 to 1:4) under Ar atmosphere, desired catalyst was then added and the inert atmosphere was replaced with hydrogen atmosphere (1 atm). The reaction was followed using GC-MS: 20 µL of reaction mixture was diluted with 180 µL of DCM, filtered with an on-line Luer lock filter (Nylon, 0.44 micron, Whatman), and the resulting solution was analysed. When the reaction was complete, the hydrogen atmosphere was replaced with an argon atmosphere, then the suspension was filtered on a short pad of silica and concentrated under vacuum. The residue was purified on a silica gel column with 95:5 hexanes: EtOAc as eluent and analysed. The obtained results are depicted in Table S1, the reaction conditions described in entry 6 have been adopted for the preparation of 8,9-dihydrocannabidiol (DHD, **5**) on 50 mg scale, whereas conditions described in entry 1 were adopted for the preparation of tetrahydrocannabidiol (THD, **7**).

Table S1. Optimization of regioselective hydrogenation conditions.

Entry	*Catalyst/amount*	*Solvent[a]*	*Reaction time*	*Ratio* DHD (**5**) THD (**7**)[b]	*Isolated Yield*
1	**Pd/C 10%, 20% w/w**	**4:1**	**1h**	**0:100**	**96%**
2	Pd-$CaCO_3$ poisoned (Lindlar cat.)[e], 40% w/w	1:1	8h	80:20	93%
3	Pd-$CaCO_3$ poisoned (Lindlar cat.)[e], 20%w/w	4:1	54h	>99:1	53%[c]
4	“ “	1:1	28h	90:10	65%[c]
5	“ “	1:1[d]	72h	>99:1	51%[c]
6	**Pd-$CaCO_3$ poisoned (Lindlar cat.)[e], 15%w/w**	**2:1**	**23h**	**>99:1**	**97%**

[a] = *n*-hexane/EtOAc mixture; [b] = Inseparable mixture of compounds, ratio was determined using GC-MS; [c] = long reaction time (more than 30-36 h) increases considerably the amount of polar sideproducts not well defined; [d] = 10%v/v of cyclopentene was employed as an overreduction inhibitor. [e] Lindlar Catalyst, 5% Pd on $CaCO_3$ poisoned with lead was purchased from Fluka Chemika, cat. Number: 62145.

3.6.1. Synthesis of 8,9-dihydrocannabidiol (DHD, 5)

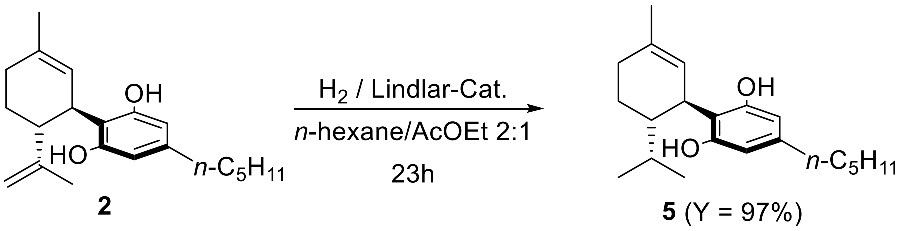

Cannabidiol (CBD, **2**, 50 mg, 0.16 mmol) was dissolved in a dry 2:1 mixture of *n*-hexane:EtOAc(3 mL) under an Ar atmosphere. Palladium 5% on calcium carbonate poisoned with lead (Lindlar Cat., 7.5 mg, 15% w/w) was added under magnetic stirring and Ar atmosphere was replaced with a hydrogen static atmosphere (1 atm). The reaction mixture was stirred for 23 h. Catalyst was then removed by filtration, volatiles were removed under vacuum and the residue was filtered on silica gel short pad to give 8,9-dihydrocannabidiol (DHD, **5**) (48.5 mg, yield = 97%).

8,9-dihydrocannabidiol (DHD, 5). ^{1}H NMR (300 MHz, $CDCl_3$) δ 8.48 – 6.52 (m, 2H), 6.22 (s, 2H), 5.35 (dq, *J* = 3.0, 1.4 Hz, 1H), 3.91 (ddq, *J* = 10.9, 4.5, 2.4 Hz, 1H), 2.58 – 2.29 (m, 2H), 2.25 – 2.01 (m, 2H), 1.95 (ddt, *J* = 12.9, 10.5, 2.5 Hz, 1H), 1.79 (ddt, *J* = 12.8, 5.1, 2.5 Hz, 1H), 1.73 – 1.68 (m, 3H), 1.67 – 1.47 (m, 2H), 1.46 – 1.19 (m, 5H), 1.00 – 0.72 (m, 9H). **^{13}C NMR** (75 MHz, $CDCl_3$) δ 157.7, 143.0, 136.6, 127.2, 115.6, 108.8, 43.9, 36.8, 36.6, 32.7, 32.0, 31.8, 24.0, 23.8, 23.6, 22.3, 17.1, 14.7. HRMS (ESI) *m/z* calcd. For $C_{21}H_{31}O_2$ [M+H$^+$]: 315.2319, found 315.2317.

3.6.2. Synthesis of tetrahydrocannabidiol (THD, 7)

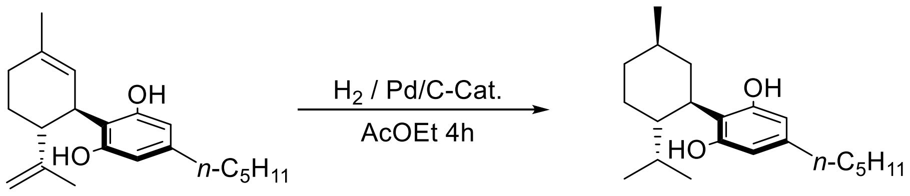

2 **7** (Y = 98%)

Cannabidiol (CBD, **2**, 43 mg, 0.14 mmol) was dissolved in EtOAc (4 mL) under an Ar atmosphere. Palladium on charcoal (10 % of Pd, 8 mg, 20 % w/w) was added under magnetic stirring and the Ar atmosphere was replaced with a hydrogen static atmosphere (1 atm). The reaction mixture was stirred for 4 h and then a portion of solution (20 µL of reaction mixture was then diluted with 180 µL of DCM and then filtered with an on-line Luer lock filter) was analysed with GC-MS. Palladium catalyst was then removed by filtration, volatiles were removed under vacuum and the residue was filtered on silica gel short pad to give tetrahydrocannabidiol (**7**) as a 9:1 mixture of diastereoisomer (43.6 mg, yield = 98%).

Tetrahydrocannabidiol (THD, 7). ^{1}**H NMR** (300 MHz, CDCl3) δ 6.33 – 5.97 (m, 2H), 4.65 (s, 2H), 3.01 (td, J = 11.2, 4.1 Hz, 1H), 2.45 (dd, *J* = 9.0, 6.6 Hz, 2H), 2.04 (tt, *J* = 11.3, 3.0 Hz, 1H), 1.92 – 1.43 (m, 6H), 1.33 (h, *J* = 4.2 Hz, 5H), 1.18 – 0.80 (m, 12H), 0.73 (d, *J* = 6.9 Hz, 3H). ^{13}**C NMR** (75 MHz, $CDCl_3$)) δ 155.3, 154.0, 141.8, 115.0, 109.0, 108.0, 44.6, 40.1, 38.1, 35.3, 35.2, 33.4, 31.5, 30.5, 28.5, 25.3, 22.4, 22.3, 21.6, 15.7, 13.9.

3.7. Synthesis of hexahydrocannabinol (HHC)

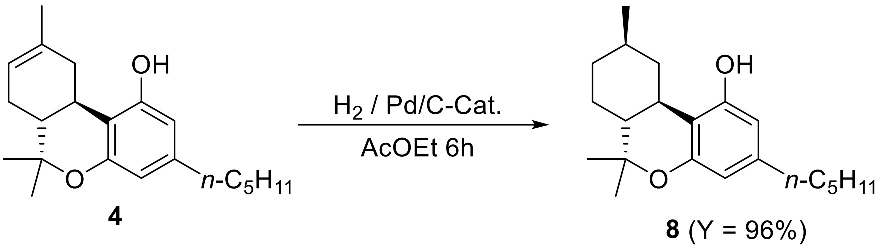

Δ^{8}-THC (**4**, 48 mg, 0.15 mmol) was dissolved in EtOAc (4 mL) under an Ar atmosphere. Palladium on charcoal (10 % of Pd, 9.6 mg, 20 % w/w) was added under magnetic stirring and Ar atmosphere was replaced with a hydrogen static atmosphere (1 atm). The reaction mixture was stirred for 6 h and then a portion of solution of reaction mixture (20 μL) was diluted with 180 μL of DCM, filtered with an on-line Luer lock filter and 15nalysed by GC-MS. Palladium catalyst was then removed by filtration, volatiles were removed under vacuum and the residue was filtered on silica gel short pad to give hexahydrocannabinol (**8**, 46.1 mg, yield = 96%).

Hexahydrocannabinol (HHC, 8). ^{1}**H NMR** (300 MHz, Acetone-*d*-6) δ 8.02 (d, *J* = 5.3 Hz, 1H), 6.21 (dd, *J* = 3.8, 1.7 Hz, 1H), 6.10 (t, *J* = 1.7 Hz, 1H), 3.34 – 3.05 (m, 1H), 2.69 (td, *J* = 11.1, 3.0 Hz, 1H), 2.55 – 2.30 (m, 3H), 1.87 (dtt, *J* = 7.9, 5.8, 2.6 Hz, 1H), 1.76 – 1.47 (m, 4H), 1.46 – 0.99 (m, 12H), 0.91 (dt, *J* = 8.5, 6.7 Hz, 6H), 0.67 (dt, *J* = 12.7, 11.3 Hz, 1H). ^{13}**C NMR** (75 MHz, Acetone-*d*-6) δ 157.6, 157.5, 156.5, 156.2, 143.0, 142.9, 111.5, 111.4, 110.0, 108.6, 108.5, 108.4, 77.3, 77.2, 51.5, 50.7, 40.2, 37.2, 36.8, 36.8, 36.5, 33.9, 33.5, 32.6, 32.0, 29.2, 29.1, 28.5, 28.3, 24.1, 23.6, 23.4, 19.7, 19.7, 19.4, 14.7. HRMS (ESI) *m/z* calcd. For $C_{21}H_{33}O_2$ [M+H^{+}]: 317.2475, found 317.2477.

4. Mass spectra of reported compounds – underivatized compounds

4.1. Δ9-THC (1)

The ten major peaks in the mass fragmentation spectra of this compound are:

m/z	*299*	*314*	*231*	*271*	*243*	*41*	*258*	*43*	*300*	*91*
Relative abundance	999	795	789	479	329	276	263	254	220	191

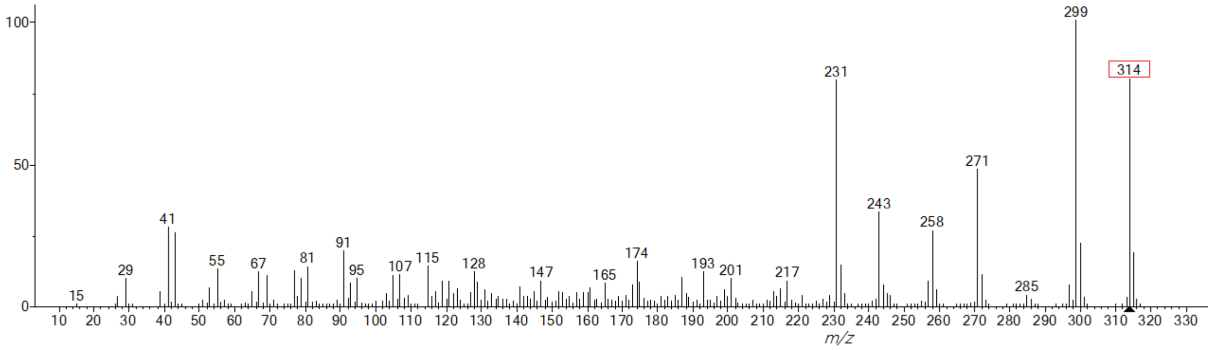

Fig. 4.1. Mass fragmentation of Δ^{9}-THC (**1**)

The main fragments of Δ^{9}-THC (**1**) are shown in Figure 4.2:

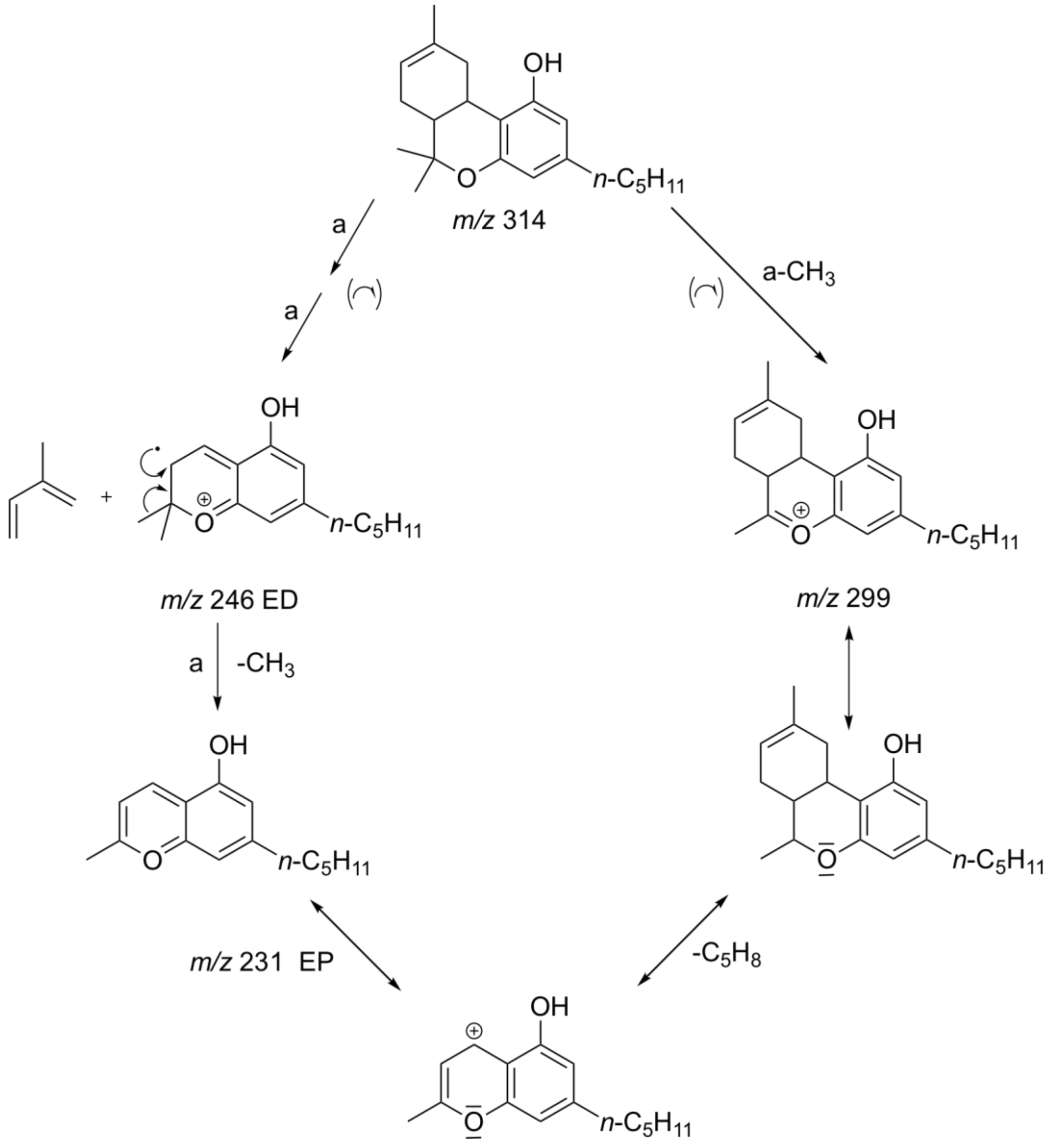

Fig. 4.2. Mass fragmentation pattern of Δ**9**-THC (**1**).

There are several α breaks and rearrangements that explain the main peaks found in the mass spectra of his and the most common cannabinoids. The Δ^9-THC (**1**) is the cannabinoid with the greatest intensity of the molecular M^+ peak at 314 m/z, also the breakdown in alpha, with the loss of a methyl, to give the peak at 299 m/z is characteristic of Δ^9-THC (**1**) and it is rarely present in the fragmentations of other cannabinoids. The two intermediates can be seen at 231 m/z, a structure of fundamental importance which appears to be the main peak of the fragmentation of most cannabinoids.

4.2. CBD (2)

The ten major peaks in the mass fragmentation spectra of this compound are:

m/z	*231*	*232*	*246*	*174*	*121*	*193*	*43*	*91*	*314*	*41*
relative abundance	999	165	125	116	113	81	77	67	66	64

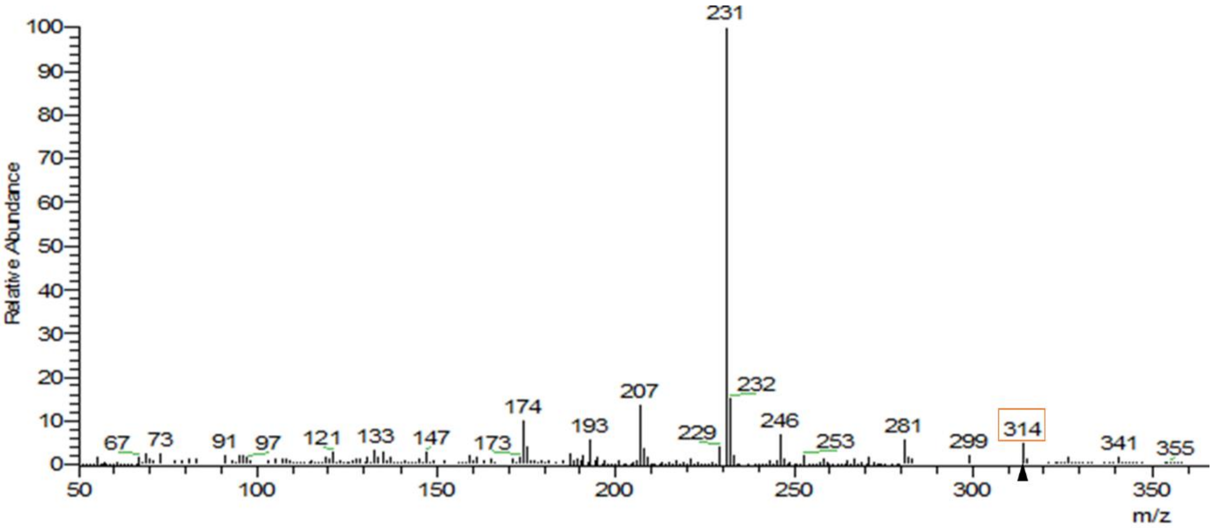

Fig. 4.3. Mass fragmentation of CBD (**2**).

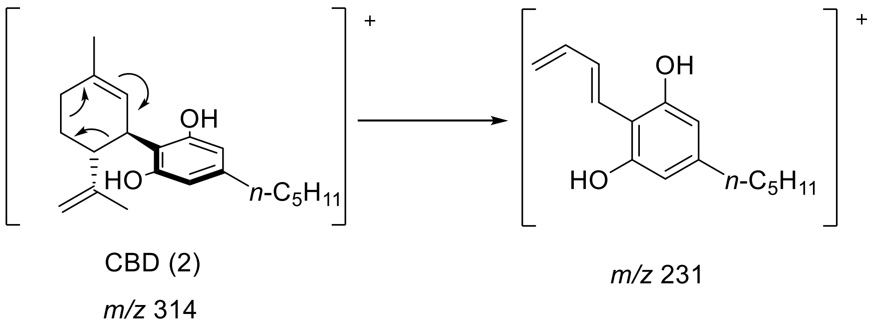

Fig. 4.4. Mass fragment at 231 m/z found in non-cyclized cannabinoids, such as CBD (**2**) .[S10]

It is one of the few cannabinoids to exhibit such a high peak intensity at 246 m/z that it corresponds to a 68 m/z loss from the molecular peak, due to the loss of an isoprene due to a double α break, similar to what shown in Figure 4.2. The peaks at 207 and 281 m/z, not present in the tabulated fragmentation are due to silanes bleeding from the GC column.

4.3. CBC (3)

The ten major peaks in the mass fragmentation spectra of this compound are:

m/z	*231*	*174*	*232*	*41*	*187*	*175*	*173*	*69*	*55*	*91*
relative abundance	999	190	161	56	46	34	33	30	25	21

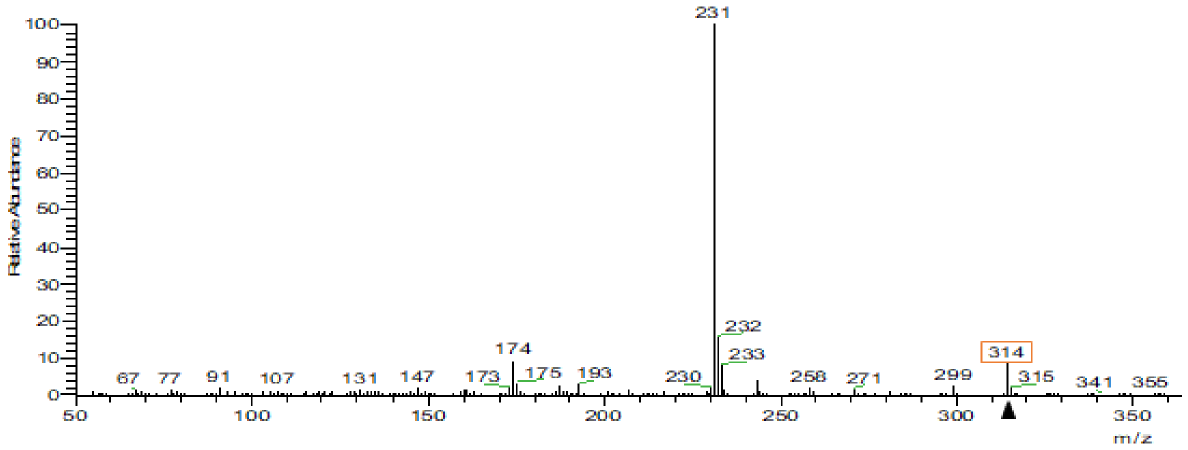

Fig. 4.5. Mass fragmentation of CBC (**3**).

Cannabichromene (**3**) possesses an extremely simple fragmentation, which has made it very difficult to identify and distinguish, as can be seen in Figure 4.5, it has a mass spectrum almost superimposable to its photodegradation derivative, cannabicyclol (Fig. 4.7). It was possible to distinguish it from CBL only after the synthesis of the CBC standard, by evaluating the different retention times in GC-MS. The characteristic fragmentation of these cannabinoids at 231 and 232 m/z can be explained, commonly to THC, by the two intermediates shown in Figure 4.6.

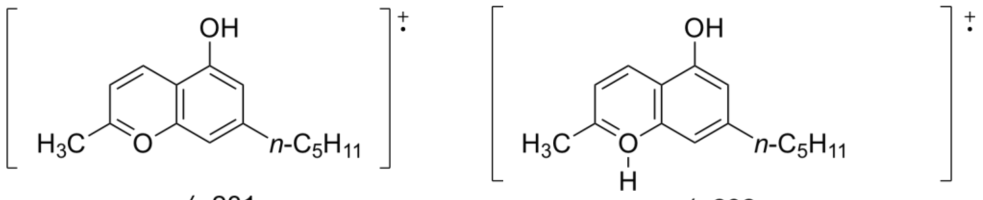

m/z 231 *m/z* 232

Fig. 4.6 Mass fragments at 231 m/z and 232 m/z, commonly found in cyclized cannabinoids.

4.4. CBL (11)

The ten major peaks in the mass fragmentation spectra of this compound are:

m/z	*231*	*232*	*174*	*41*	*314*	*69*	*187*	*55*	*81*	*299*
relative abundance	999	169	126	52	38	36	34	27	27	26

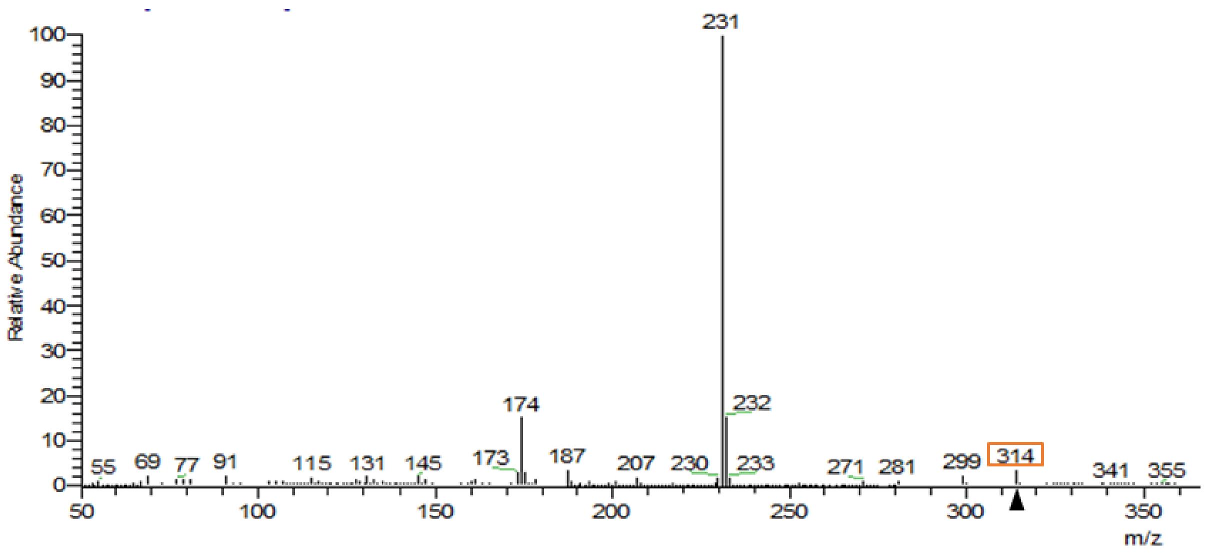

Fig. 4.7. Mass fragmentation of CBL (**11**).

The main advantage of mass coupled gas chromatography lies in the possibility of having a double comparison on the data, both of the fragmentation and of the retention times. CBL (**11**), despite the almost identical fragmentation to that of CBC (**3**), has a significantly different retention time; this allowed, with the injection of appropriate standards, to recognize them uniquely. CBL (**11**) has never been recognized in the irradiations carried out although it is reported in the literature as the cyclized product of a photochemically catalyzed 2 + 2 cycloaddition of CBC (**3**).[S11]

4.5. CBT (12)

The ten major peaks in the mass fragmentation spectra of this compound are:

m/z	*231*	*232*	*314*	*233*	*174*	*41*	*271*	*299*	*258*	*43*
relative abundance	999	199	167	99	98	95	77	65	60	54

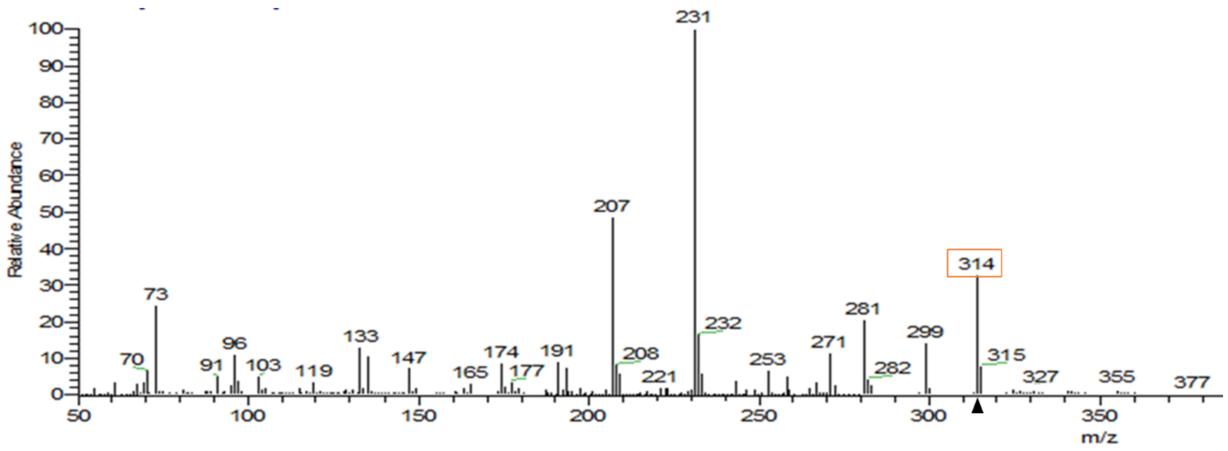

Fig. 4.8. Mass fragmentation of CBT (**12**).

CBT (**12**) is extremely easy to identify in derivatized samples as it does not undergo the derivatization process, as there are no hydroxyl groups, thus presenting an unaltered mass of 314 uma. From its fragmentation we can deduce the peaks at 299, due to the breakdown in α of a methyl, the peaks at 271 m/z, due to the loss of a chain with 3 atoms of C and the characteristic peak of cannabinoids at 231 m/z. The peaks at 207 and 281 m/z, not present in the tabulated fragmentation are due to silanes bleeding from the GC column.

4.6. CBG (10)

The ten major peaks in the mass fragmentation spectra of this compound are:

m/z	*193*	*231*	*123*	*43*	*69*	*194*	*136*	*122*	*316*	*121*
relative abundance	999	291	251	235	202	201	188	187	166	103

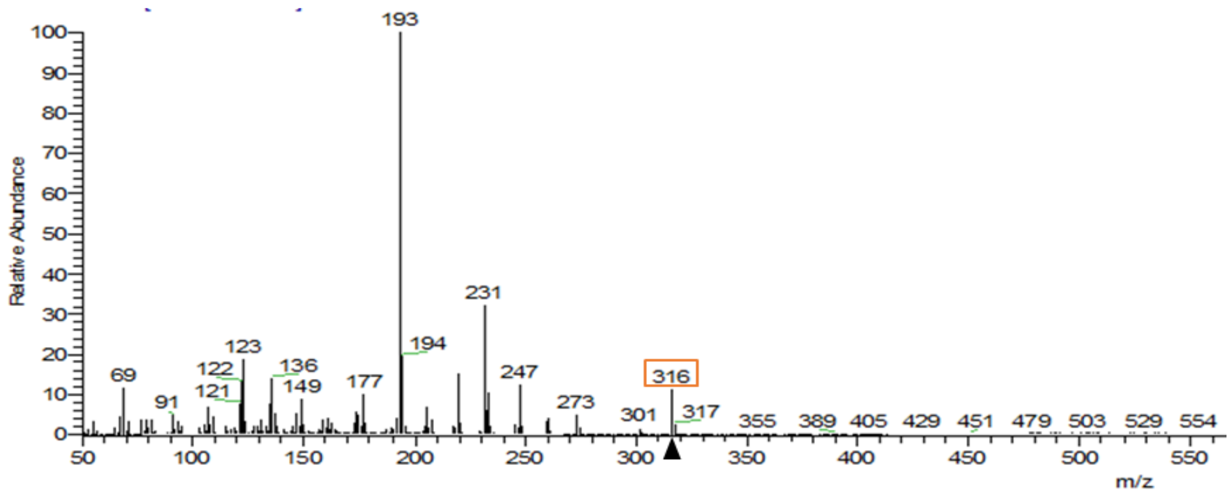

Fig. 4.9. Mass fragmentation of CBG (**10**).

CBG is one of the few cannabinoids not to have 231 m/z as a base peak, although it is still the second most abundant. It can be noted the characteristic molecular peak M^+ at 316 m/z due to the fact that, having only one ring, it has less unsaturation than the other cannabinoids considered. With the short ramp it was not possible to identify it clearly, as it is in an area where there is a high background signal, which is why it was decided to change the analysis method using a slower temperature ramp. The peak at 193 m/z can be attributed to the breakdown of the isoprene side chain as reported for the derivatized counterpart in Figure 4.16, i.e. a structure similar to a methyl-olivetol.

4.7. CBN (13)

The ten major peaks in the mass fragmentation spectra of this compound are:

m/z	*295*	*296*	*238*	*310*	*165*	*223*	*251*	*239*	*119*	*152*
Relative abundance	999	227	141	111	47	43	41	34	33	30

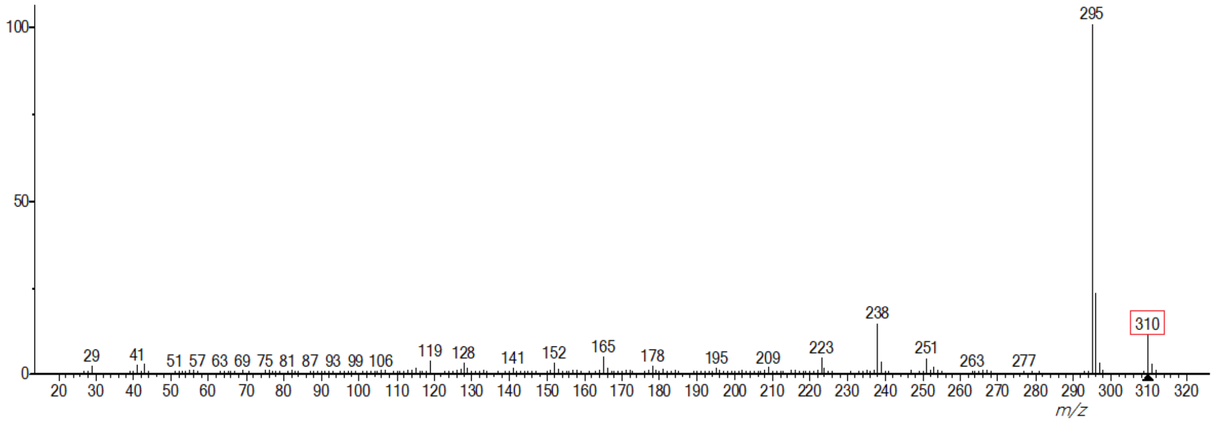

Fig. 4.10. Mass fragmentation of CBN (**13**).

Characteristic of CBN (**13**) is the molecular peak M^+ at 310 m/z due to two more unsaturations than THCs, a consequence of the aromatization of the terpene ring. From the spectrum, the base peak, with the greatest intensity, is 295 m/z corresponding to the loss of a methyl group. Also in this case, as for the CBG (**10**), with the short ramp it was not possible to distinguish it from the bottom.

4.8. DHD (5)

The ten major peaks in the mass fragmentation spectra of this compound are:

m/z	*231*	*246*	*316*	*22*	*193*	*174*	*273*	*260*	*229*	*175*
relative abundance	999	180	163	132	112	71	61	59	52	37

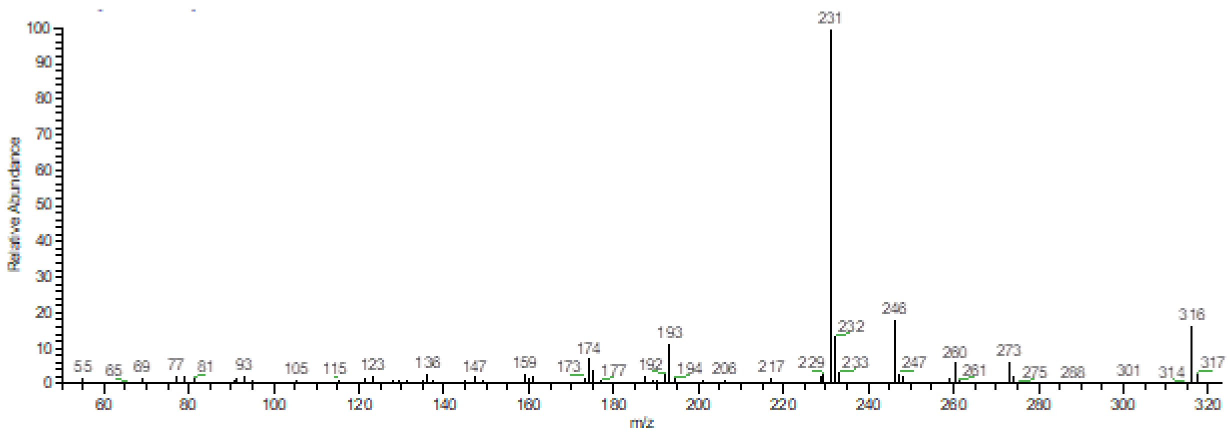

Fig. 4.11. Mass fragmentation of DHD (**5**).

DHD (**5**) has a very similar fragmentation to CBD (**2**), to which the reader is referred; they differ only in molecular weight, which leads to the 316 m/z in the case of DHD (**5**).

4.9. THD (7)

Cis- isomer

The ten major peaks in the mass fragmentation spectra of this compound are:

m/z	*193*	*233*	*318*	*262*	*194*	*136*	*234*	*123*	*150*	*124*
relative abundance	999	663	258	158	122	107	92	88	68	59

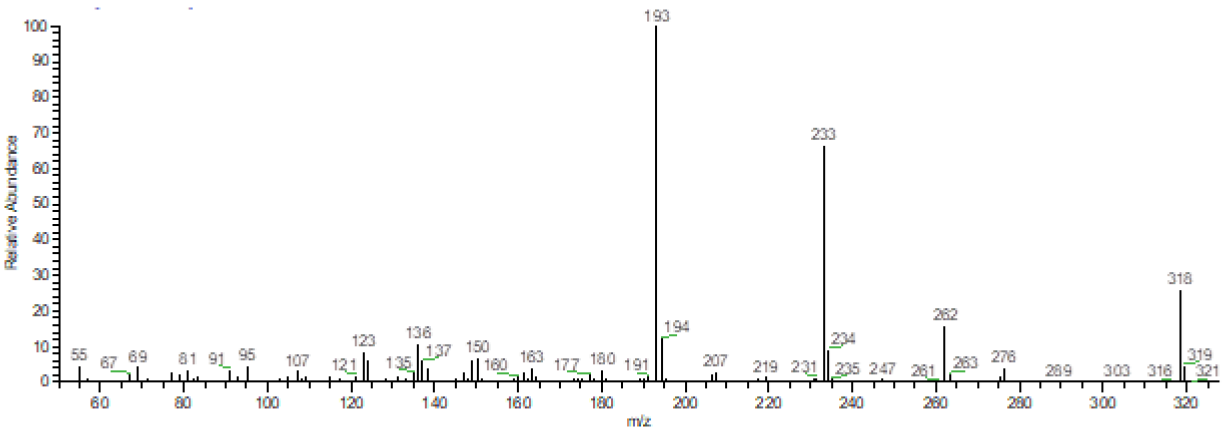

Fig. 4.12. Mass fragmentation of *cis*-THD (*7*).

Trans- isomer

The ten major peaks in the mass fragmentation spectra of this compound are:

m/z	*193*	*233*	*318*	*262*	*194*	*136*	*123*	*234*	*150*	*149*
relative abundance	999	650	232	129	123	113	103	95	79	71

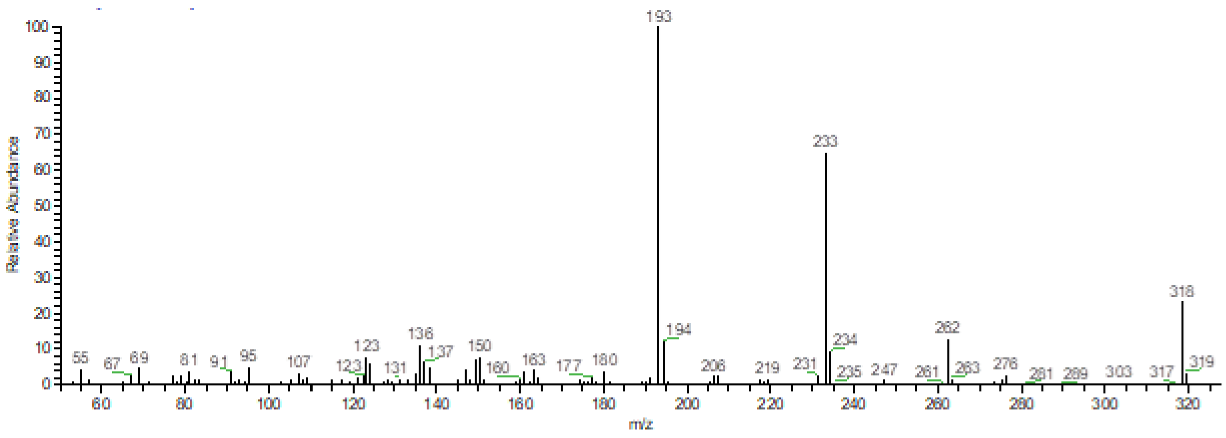

Fig. 4.13. Mass fragmentation of *trans*-THD (**7**).

The *cis* and *trans* THD (**7**) isomers have comparable fragmentation profile but different retention index. The loss of a methyl group is not significant as it cannot generate any system conjugated to the benzene ring. The fragment 262 m/z corresponds to the loss of butylene, while the origin of 193 m/z will be the same described in the case of HHC (**8**). The most abundant fragment is 233 m/z and below (Figure 4.14) a possible origin is shown (l and l ‘).[S12]

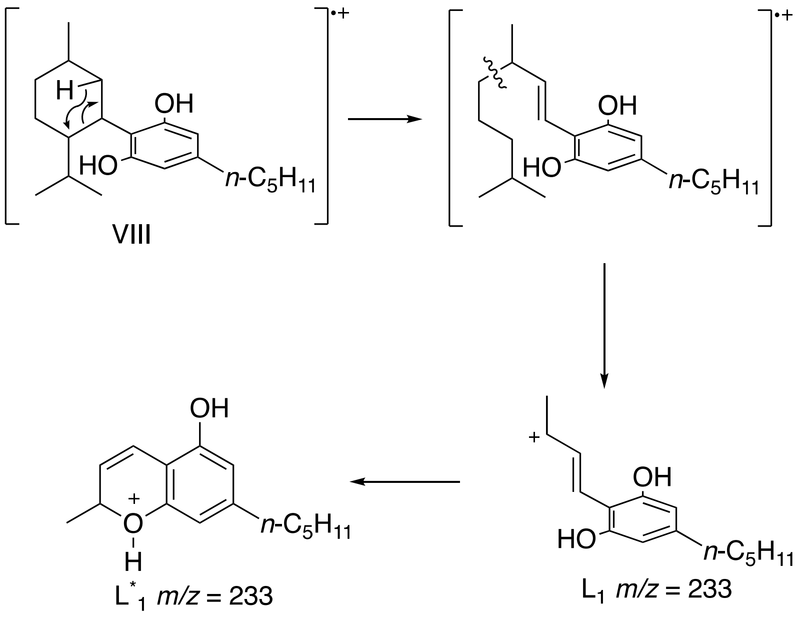

Fig. 4.14. Fragmentation of THD (**7**).

4.10 HHC (8)

***Cis*- isomer**

The ten major peaks in the mass fragmentation spectra of this compound are:

m/z	*273*	*260*	*193*	*316*	*274*	*231*	*233*	*136*	*261*	*317*
relative abundance	999	979	748	609	368	278	184	177	164	146

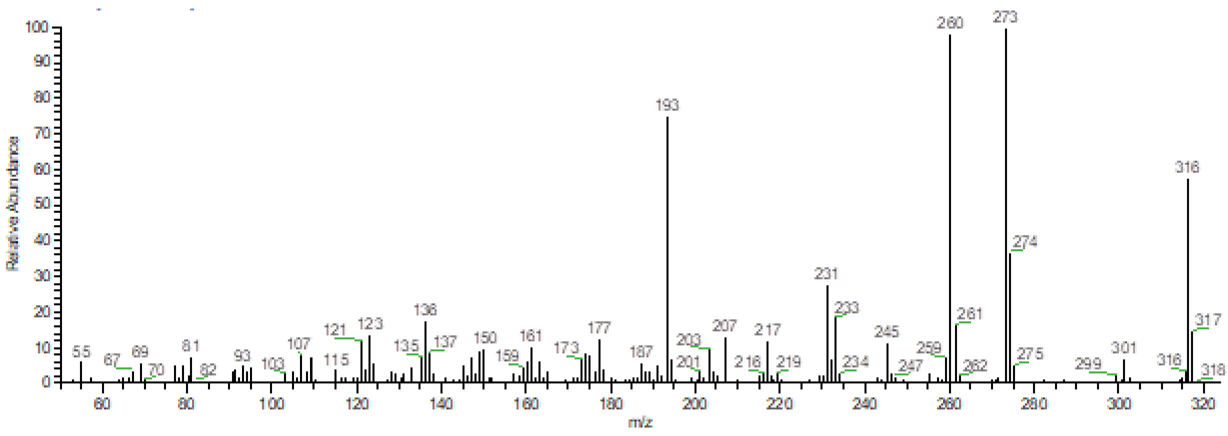

Fig. 4.15. Mass fragmentation of cis-HHC (cis-**8**).

***Trans*- isomer**

The ten major peaks in the mass fragmentation spectra of this compound are:

m/z	*273*	*260*	*193*	*316*	*274*	*231*	*136*	*233*	*177*	*123*
relative abundance	999	791	726	570	327	289	207	165	158	147

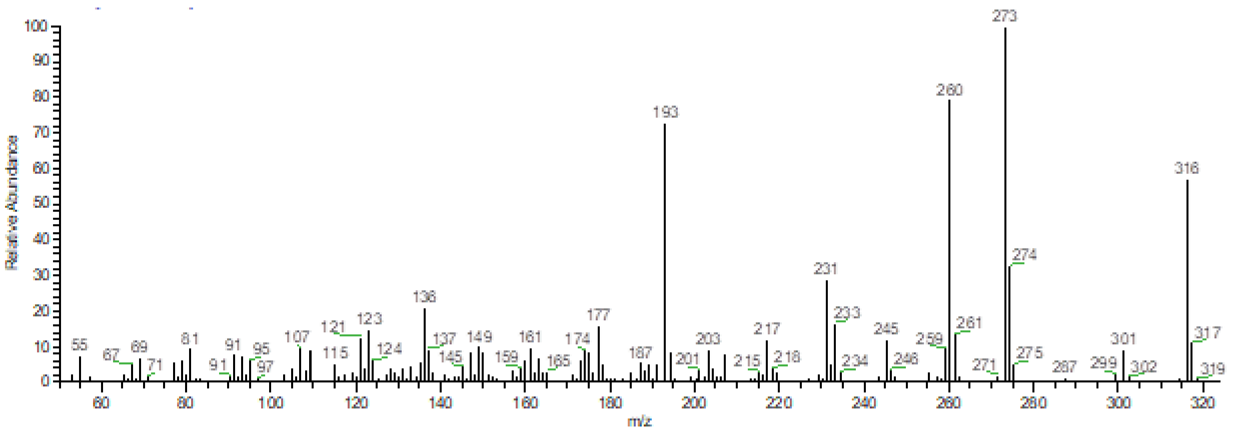

Fig. 4.16. Mass fragmentation of trans-HHC (trans-**8**).

HHC (**8**) is obtained as a mixture of two stereoisomeric forms with an equatorial and an axial methyl group C-1, deriving from the hydrogenation of Δ^8-THC (**4**), but the mass fragmentation spectra of the two compounds are identical. The 301 m/z fragment is obtained from the loss of a methyl group, while the 273 m/z fragment from the loss of a $C_3H_7\cdot$ from the parent molecule. The 260 m/z fragment derives from the loss of a butylene and the 245 m/z fragment from the loss of a methyl and butyl group from HHC (**8**). From the decomposition of the cyclohexane ring, ethylene, propylene and the 246 m/z fragment are formed, which can result from the loss of a methyl group. From 246 m/z due to the loss of a methyl group 231 m/z is obtained (Figure 4.17).

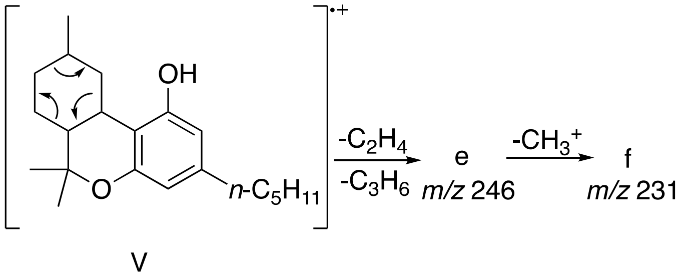

Fig. 4.17. Fragmentation of HHC (**8**).

Among the most abundant fragments 193 m/z (benzyl ion) was find, which is obtained from a series of rearrangement reactions (Figure 4.18).[S12]

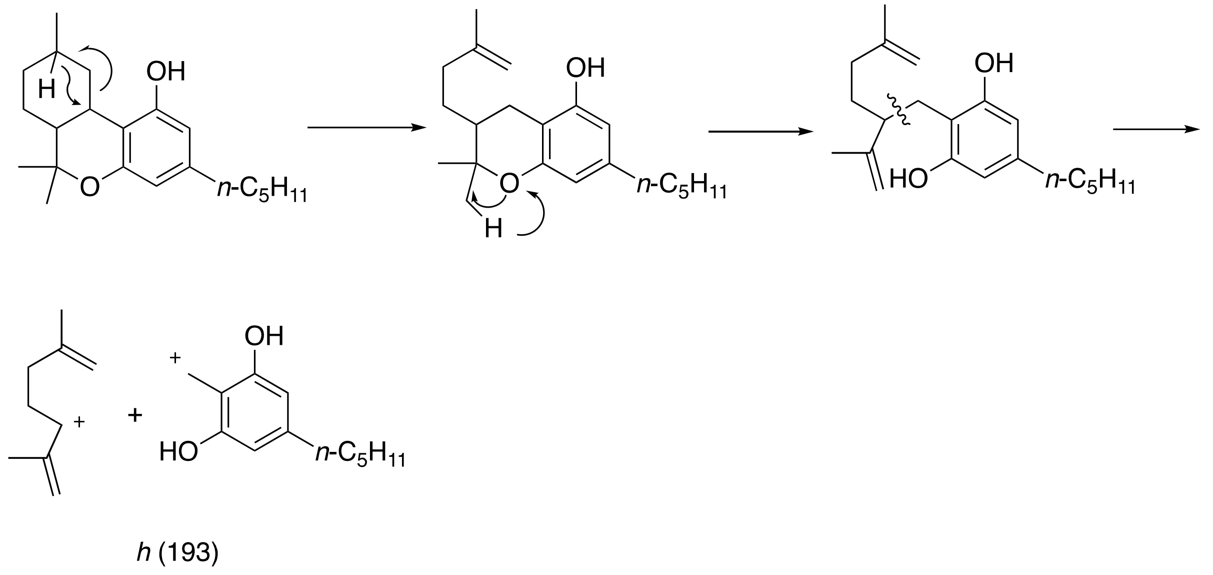

Fig. 4.18. Fragment formation m/z 193 for HHC (**8**).

4.11. Δ[8]-THC (4)

The ten major peaks in the mass fragmentation spectra of this compound are:

m/z	*231*	*314*	*258*	*271*	*174*	*193*	*201*	*232*	*207*	*119*
relative abundance	999	387	326	282	247	203	177	154	116	114

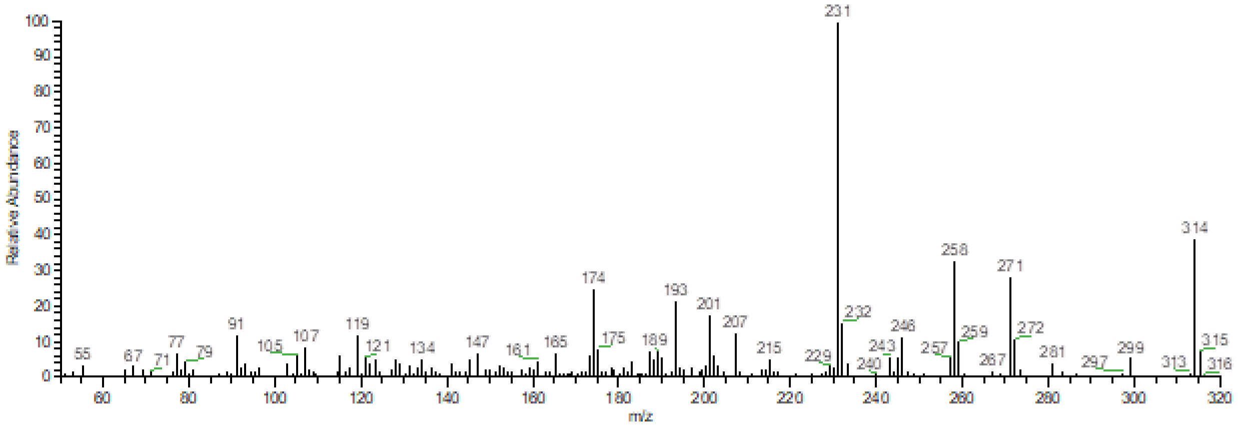

Fig. 4.19. Mass fragmentation of Δ^8-THC (**4**).

From Δ8-THC (**4**), a retro-Diels-Alder decay could occur in which the 246 m/z fragment originates and from this, through the loss of one of the geminal methyl groups, the 231 m/z fragment is obtained (Figure 4.20) . The 207 m/z fragment is due to silanes bleeding from the GC column.

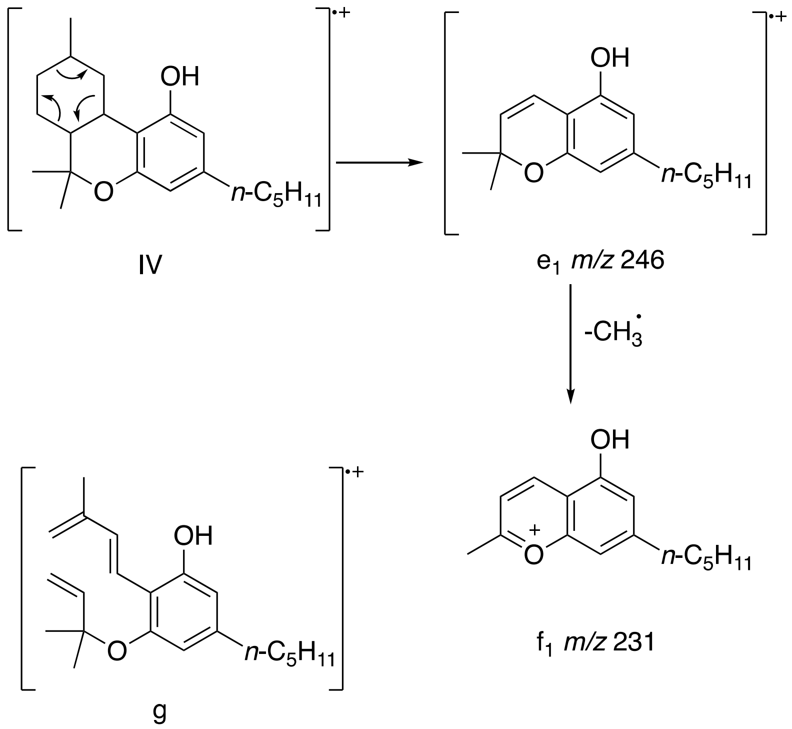

Fig. 4.20. Origin of fragment m/z 246 and m/z 231.

The retro-Diels-Alder decay also generates the fragment g (Figure 4.20), the fragments 68 m/z (from 246 m/z) and 86 m/z (from 231 m/z), via further rearrangement reactions.[S12]

4.12. Δ8-iso-THC (9)

The ten major peaks in the mass fragmentation spectra of this compound are:

m/z	*231*	*232*	*314*	*174*	*233*	*243*	*193*	*299*	*315*	*229*
relative abundance	999	153	126	81	81	43	30	27	22	21

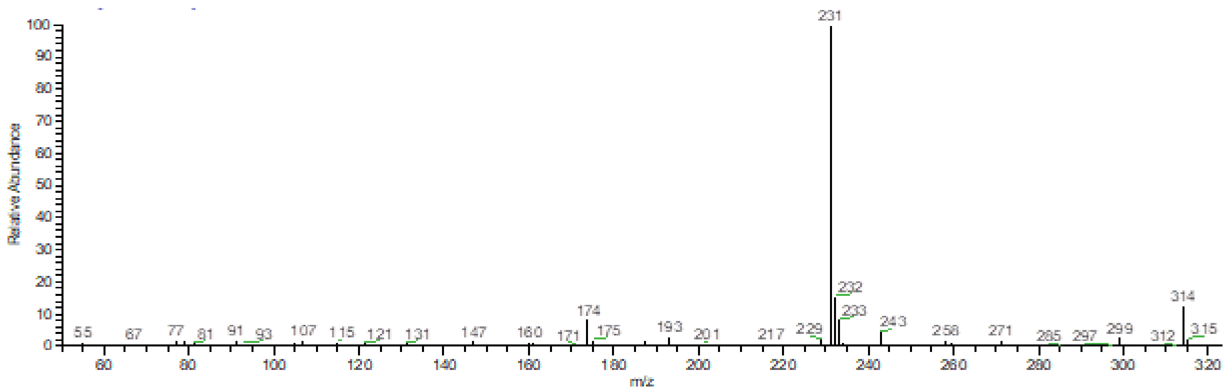

Fig. 4.21. Mass fragmentation of Δ[8]-iso-THC (**9**).

Δ[8]-iso-THC (**9**) has a mass spectrum similar to that of CBD (**2**).

4.13. Δ[7]-CBD (6)

The ten major peaks in the mass fragmentation spectra of this compound are:

m/z	*231*	*193*	*232*	*314*	*243*	*233*	*271*	*299*	*107*	*91*
relative abundance	999	382	134	133	96	92	78	76	57	51

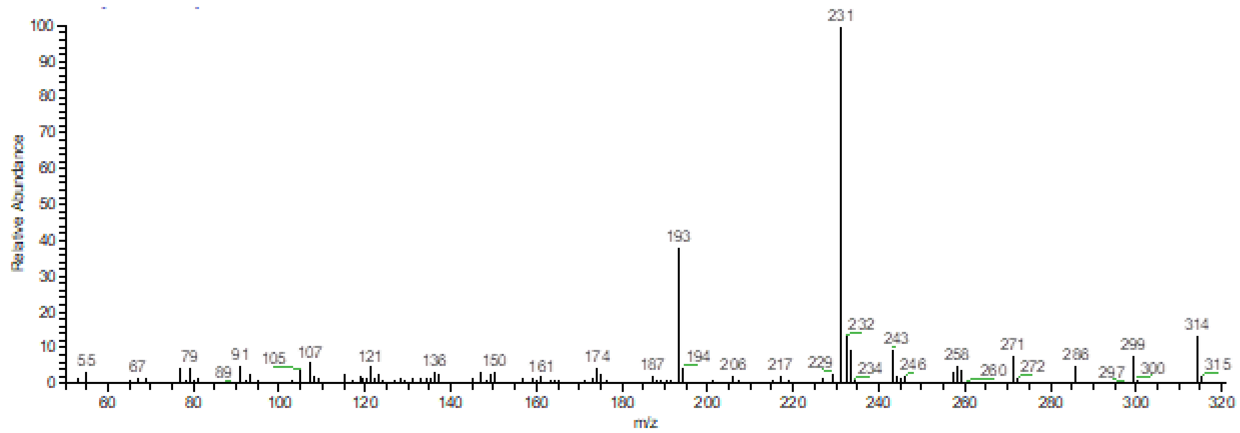

Fig. 4.22. Mass fragmentation of Δ[7]-CBD (**6**).

The higher intensity fragments, 299, 271, 231 and 193 m/z , are the same as observed in the case of CBD (**2**). As can be expected, the relationship between the different molecular ions present in the spectrum is however different than in the case of CBD (**2**).

5. Mass spectra of reported compounds - derivatized compounds

A characteristic of derivatized compounds is that they often have fragmentations similar to those of underivatized cannabinoids, and the entire spectrum is simply translated by 72 m/z or by 144 m/z, based on the number of TMS binding to the molecules. The retention times of TMS-derivatized compounds are reduced with respect to the parent cannabinoids due to their lower affinity for the stationary phase of the gas chromatography column. Derivatization makes it possible to clearly identify the number of free -OH groups of the test substance, based on the number of TMS residues inserted in the structure itself. The tabulated values of the retention indices are not always available in the literature, therefore they have not been reported above, in Table 2.1.

5.1. CBD-2TMS

The ten major peaks in the mass fragmentation spectra of this compound are:

m/z	*390*	*73*	*337*	*391*	*301*	*351*	*319*	*338*	*324*	*392*
relative abundance	999	650	499	344	299	205	162	152	143	125

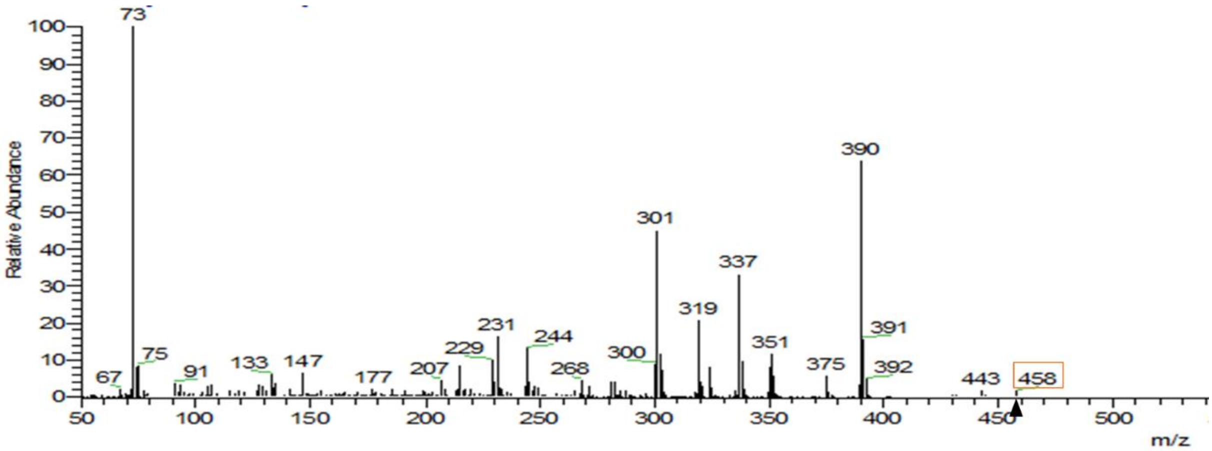

Fig. 5.1. Mass fragmentation of CBD-2TMS.

When CBD is derivatized, the molecular peak intensity (M^{+}= 458 m/z) decreases and some fragments' intensity increase, as they are probably partially stabilized by the two linked TMS groups.

The fragmentation, due to the two TMS groups, is influenced by the same, both with a shift of +144 m/z, and to a variation in the intensity of the peaks. The fundamental peaks, however, can be compared to those found in the non-derivatized spectrum: the base peak at 390 m/z corresponds to the non-derivatized fragment at 246 m/z (+144 m/z), whose intensity has greatly increased due to of the different stability. This fragment can be explained by the loss of the isoprene fragment from the non-aromatic ring. The opposite effect was instead suffered by the peak related to the characteristic fragmentation of cannabinoids at 231 m/z, now corresponding to the derivatized fragment at 375 m/z, which has undergone a drastic decrease in intensity. The 319 m/z fragment could be attributed to the resorcinol moiety obtained after the breaking of the bond between the two rings of the molecule. The peak at 337 m/z is the bis-derivatized counterpart of the peak at 193 m/z, also found in other cannabinoids such as CBC and CBL.

5.2. Δ^9-THC-TMS

The ten major peaks in the mass fragmentation spectra of this compound are:

m/z	*315*	*73*	*371*	*386*	*303*	*343*	*95*	*316*	*330*	*372*
relative abundance	999	985	938	756	693	416	237	219	219	198

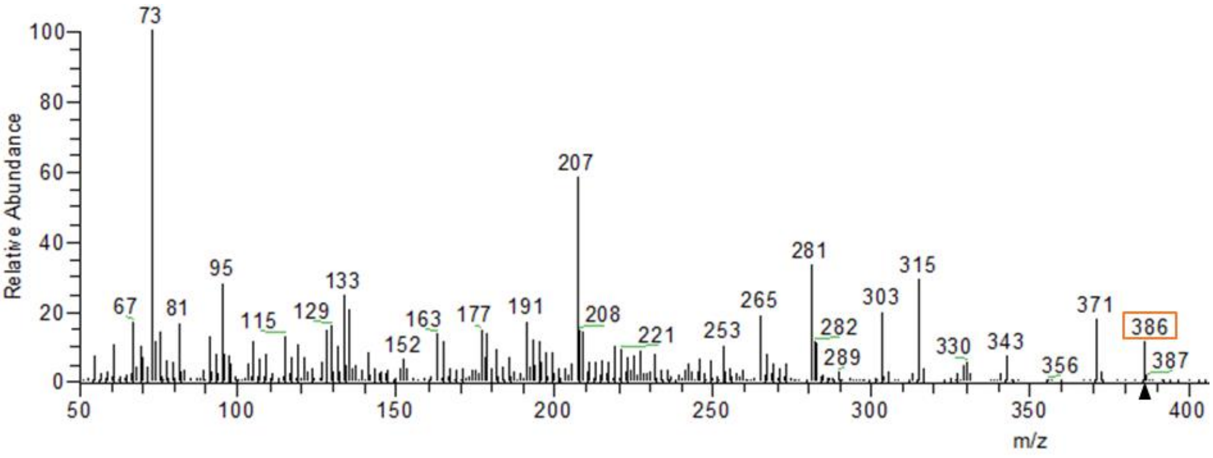

Fig. 5.2. Mass fragmentation of Δ^9-THC-TMS (**1**).

As can be seen from the molecular peak M^+ at 386 m/z, only one TMS group binds to Δ^9-THC, as an oxygen atom is part of the pyrane ring. Its fragmentation shows the easy loss of a methyl, which leads to the formation of the fragment with 371 m/z, as happened for the non-derivatized counterpart. The fragment at 303 m/z corresponds to the structure shown in Figure 4.6 with a TMS linked. Even when derivatized, Δ^9-THC has a spectrum that is easily distinguishable from other cannabinoids.

The peaks at 207 and 281 m/z are attributable to silanes bleeding from the GC column.

5.3. CBC-2TMS

The ten major peaks in the mass fragmentation spectra of this compound are:

m/z	*303*	*304*	*73*	*305*	*246*	*386*	*231*	*174*	*315*	*247*
relative abundance	999	239	113	105	65	54	38	36	34	21

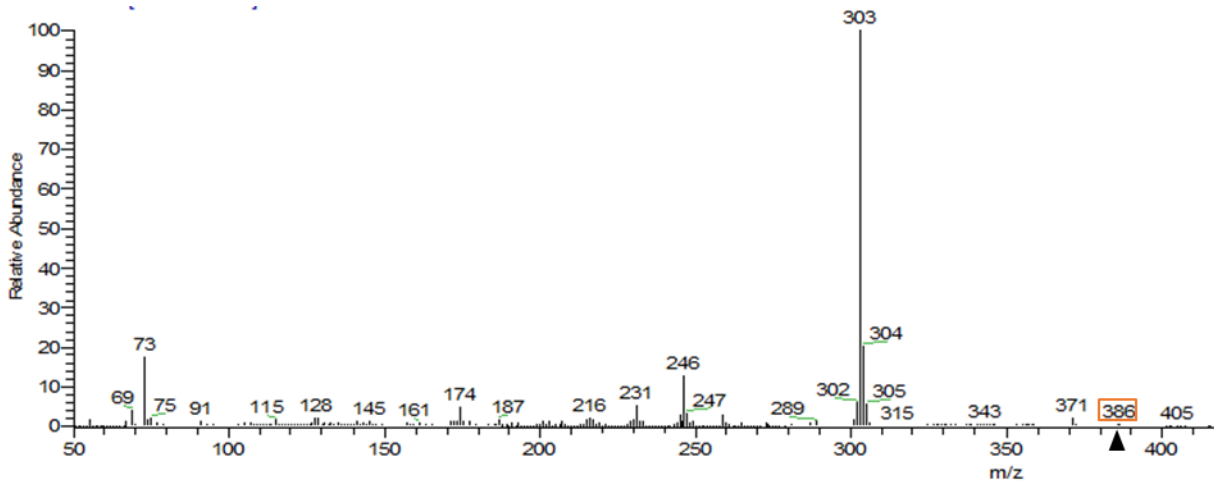

Fig. 5.3. Mass fragmentation of CBC-TMS.

The derivatized CBC was difficult to identify at first, due to the lack of spectra in the library, and also due to the extremely low intensity of the M^+ molecular peak at 386 m/z. The fragmentation follows

that of the non-derivatized compound, with a base peak at 303 m/z corresponding to the peak at 231 m/z, shown in Figure 4.3 (underivatized CBD). The spectrum provides little information on the characteristic structure of the molecule due to the limited number of peaks present.

5.4. CBL-TMS

The ten major peaks in the mass fragmentation spectra of this compound are:

m/z	*303*	*304*	*246*	*73*	*305*	*174*	*302*	*371*	*231*	*386*
relative abundance	999	580	122	119	82	45	44	44	43	35

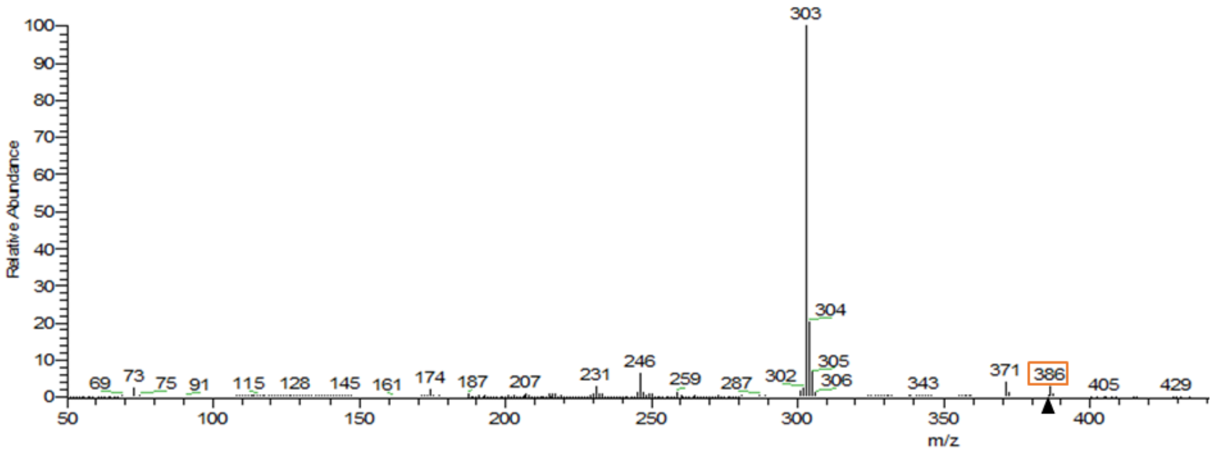

Fig. 5.4. Mass fragmentation of CBL-TMS.

Even derivatized, CBL can easily be confused with CBC (**3**), despite having a more intense molecular peak M^+ and the peak at 246 m/z with a lower relative abundance, exactly as seen without derivatization. Such small variations in the intensity of the peaks cannot be considered decisive to differentiate the two isomers with certainty, unless the different retention index is taken into account.

5.5. CBG-2TMS

The ten major peaks in the mass fragmentation spectra of this compound are:

m/z	*337*	*73*	*391*	*338*	*377*	*321*	*392*	*460*	*339*	*268*
relative abundance	999	476	394	301	177	147	130	126	112	87

a)

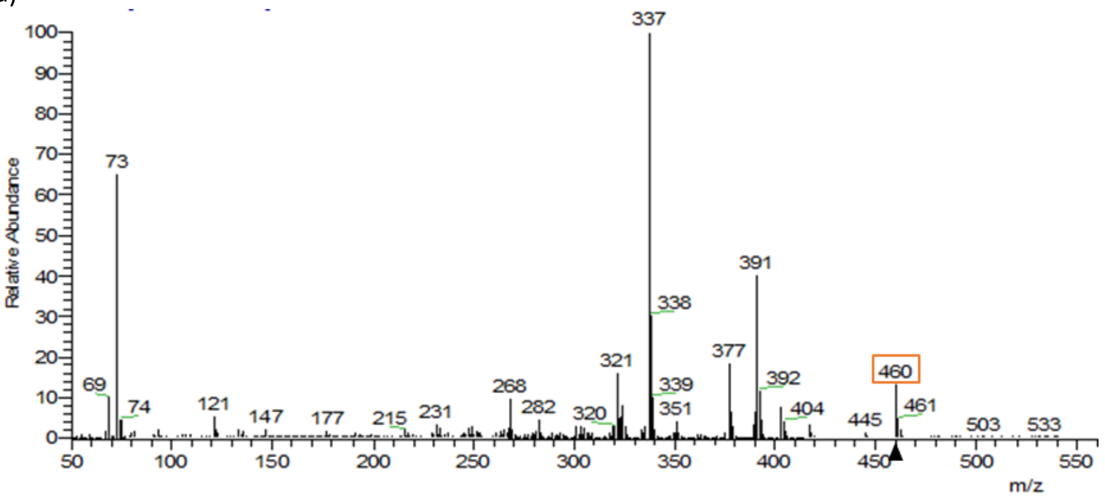

b)

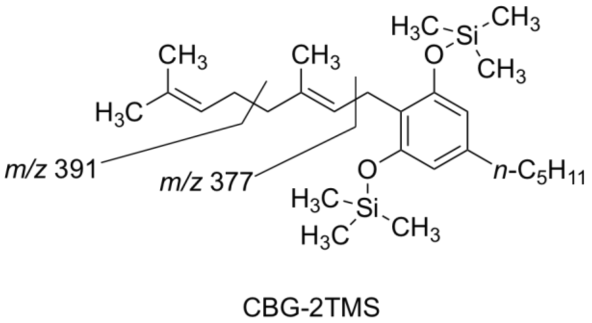

Fig. 5.5. a) Mass fragmentation of CBG-2TMS b) Principal mass fragments found in CBG-2TMS.

Cannabigerol has two free -OHs available for silylation, therefore it shows a mass increase of +144 m/z once derivatized; also in this case we can distinguish the characteristic molecular peak having +2

mass unit with respect to that of CBD. The main fragments of CBG-2TMS can be attributed to the cleavage shown in Figure 5b.

5.6. CBN-TMS

The ten major peaks in the mass fragmentation spectra of this compound are:

e m/z	*367*	*368*	*73*	*382*	*369*	*310*	*238*	*295*	*383*	*323*
relative abundance	999	309	123	101	89	63	41	37	32	21

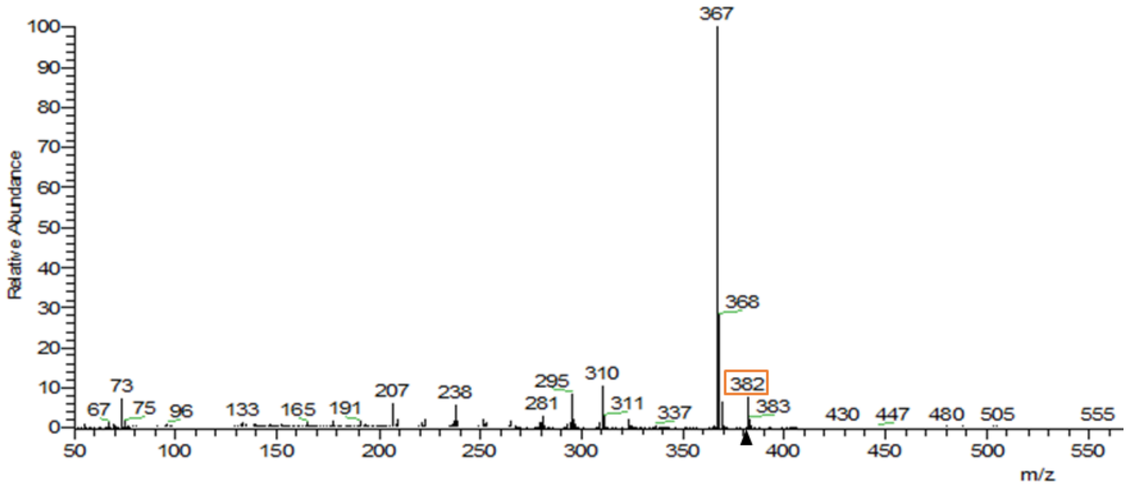

Fig. 5.6. Mass fragmentation of CBN-TMS.

CBN has a spectrum easily recognizable from other cannabinoids and has the molecular peak M^+ at 382 m/z, which is 4 units less than the Δ^9-THC from which it derives. The base peak at 367 m/z is present only in the fragmentation of this compound and is due to the loss of a methyl from the molecular peak.

5.7. DHD-2TMS

The ten major peaks in the mass fragmentation spectra of this compound are:

m/z	*390*	*73*	*391*	*301*	*319*	*460*	*392*	*324*	*321*	*302*

relative abundance	999	286	260	244	133	110	104	71	70	61

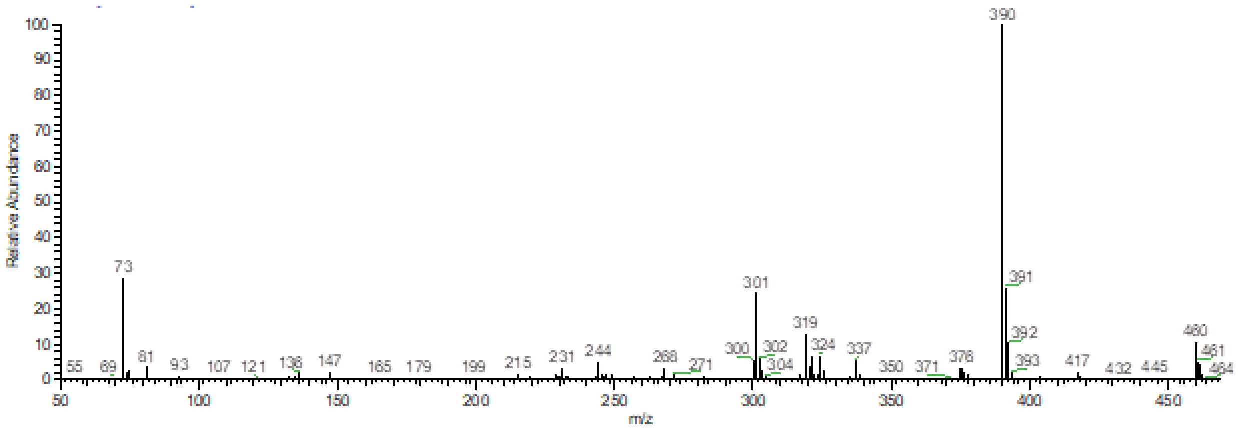

Fig. 5.7. Mass fragmentation of DHD-2TMS.

The major fragments of DHD-2TMS are: 460 m/z corresponding to the molecular ion, 390 m/z corresponding to the non-derivatized 246 m/z fragment, 319 m/z corresponding to the resorcinol fraction after breaking the bond between the two rings of the molecule, 301 m/z corresponding to -159 m/z with respect to DHD and 73 m/z due to the derivatizer.

5.8. THD-2TMS

Cis- isomer

The ten major peaks in the mass fragmentation spectra of this compound are:

m/z	*377*	*337*	*462*	*379*	*463*	*73*	*464*	*324*	*380*	*338*
relative abundance	999	347	324	277	223	216	142	125	100	85

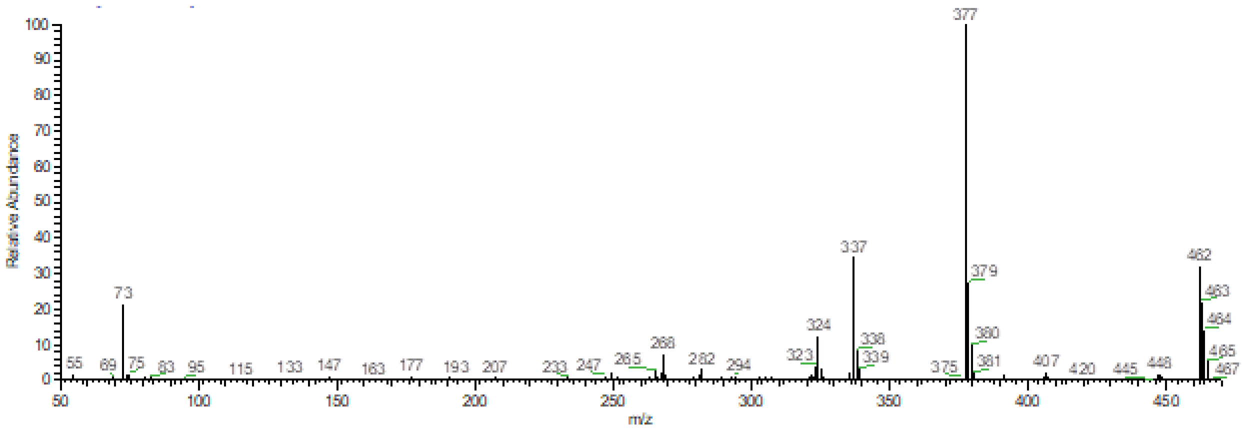

Fig. 5.8. Mass fragmentation of cis-THD-2TMS.

***Trans*- isomer**

The ten major peaks in the mass fragmentation spectra of this compound are:

m/z	*377*	*379*	*463*	*338*	*73*	*462*	*337*	*464*	*324*	*380*
relative abundance	999	309	306	255	204	182	174	145	143	105

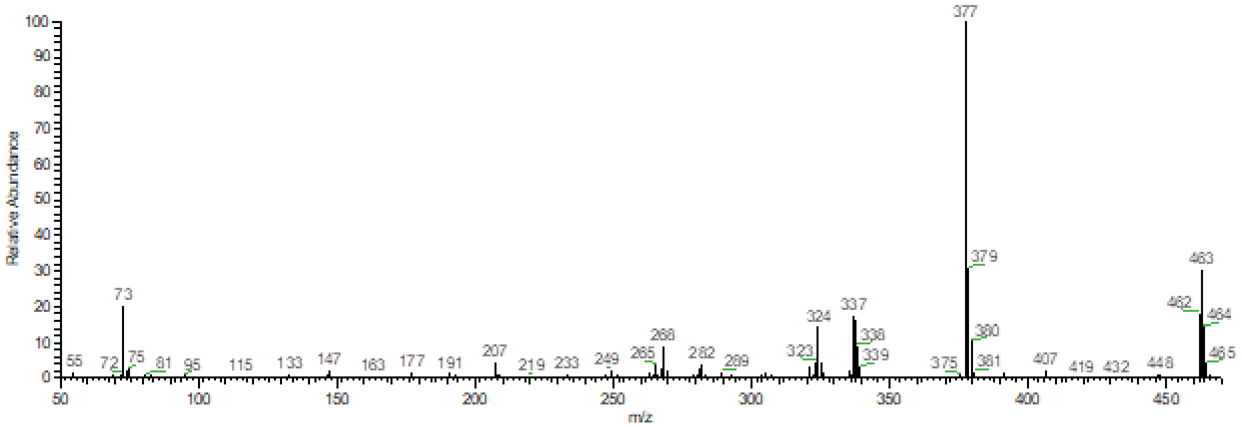

Fig. 5.9. Mass fragmentation of trans-THD-2TMS.

THD presents as principal fragments 377 m/z and 337 m/z, also present in the derivatized CBG, and 462 m/z, corresponding to the molecular ion. The spectra of *cis* and *trans* isomers are comparable, in terms of mass fragments and their relative abundance.

5.9. HHC-TMS

***Cis*- isomer**

The ten major peaks in the mass fragmentation spectra of this compound are:

m/z	*332*	*388*	*265*	*345*	*73*	*246*	*303*	*305*	*333*	*390*
relative abundance	999	780	677	658	493	407	345	256	236	216

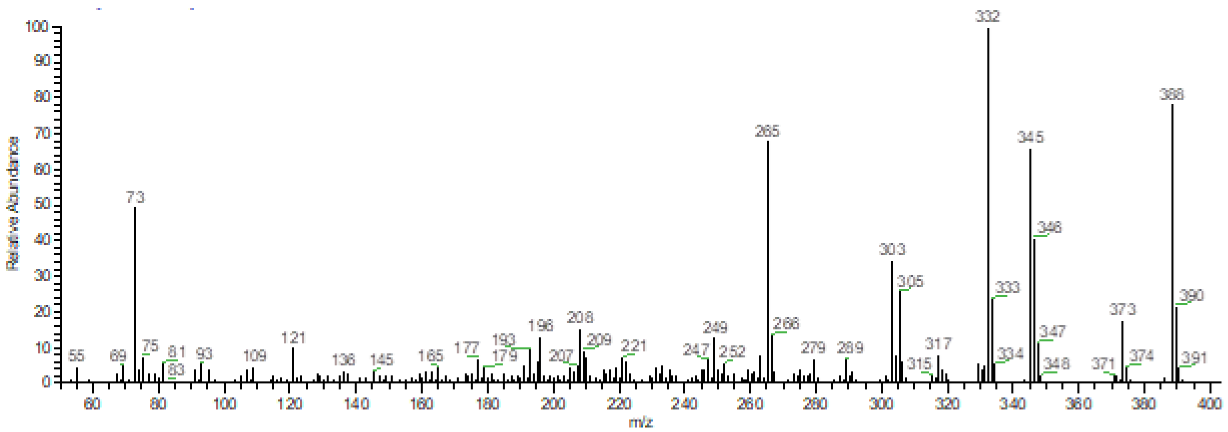

Fig. 5.10: Mass fragmentation of cis-HHC-TMS.

***Trans-* isomer**

The ten major peaks in the mass fragmentation spectra of this compound are:

m/z	*332*	*388*	*345*	*265*	*73*	*346*	*304*	*373*	*305*	*390*
relative abundance	999	849	795	781	631	408	292	288	282	269

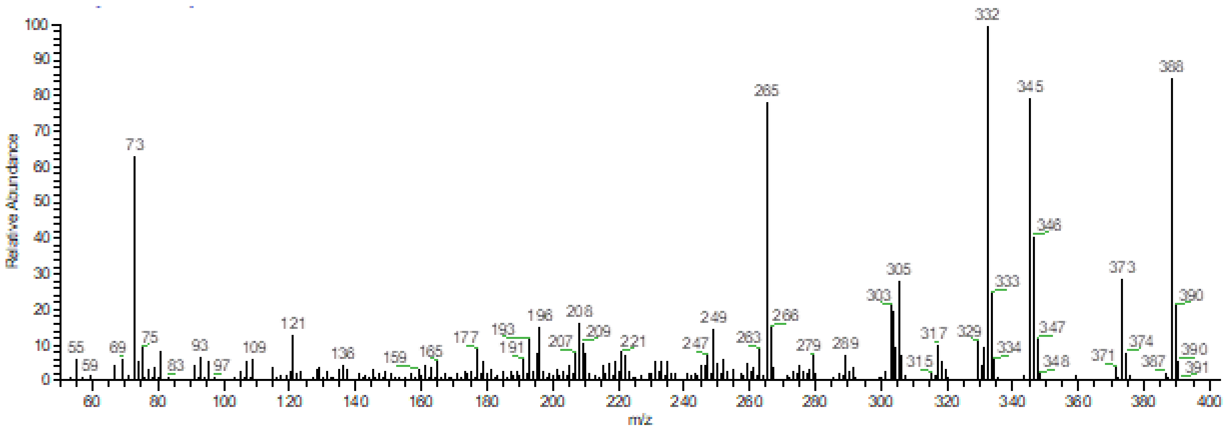

Fig. 5.11. Mass fragmentation of trans-HHC-TMS.

The molecular peak is M^+ = 388 m/z. The principal fragments are: 373 m/z corresponding to the 299 m/z peak in the non-derivatized HHC, 345 m/z corresponding to the 271 m/z peak in the non-derivatized HHC, 332 m/z corresponding to the 258 m/z peak in the non-derivatized HHC, 305 m/z corresponding to the 231 m/z peak in the non-derivatized HHC, 265 m/z corresponding to the 193 m/z peak in the non-derivatized Δ^8-THC.

5.10. Δ⁸-THC-TMS

The ten major peaks in the mass fragmentation spectra of this compound are:

m/z	*303*	*386*	*73*	*330*	*304*	*343*	*265*	*331*	*344*	*387*
relative abundance	999	440	416	368	218	190	182	147	137	120

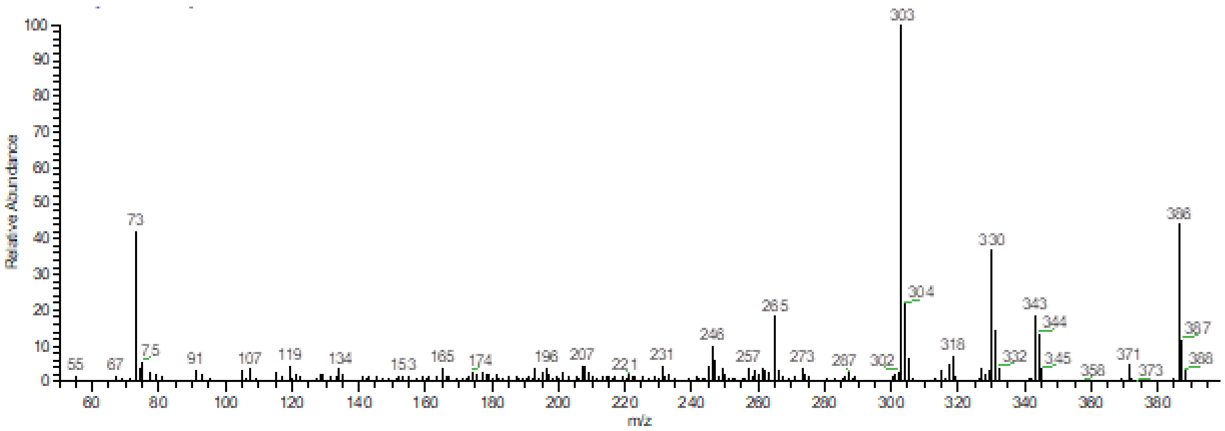

Fig. 5.12. Mass fragmentation of Δ⁸-THC-TMS.

Among the principal peaks were found: 386 m/z corresponding to the molecular peak, 343 m/z corresponding to the peak 271 m/z in the non-derivatized Δ⁸-THC (**4**), 330 m/z corresponding to the 258 m/z peak in the non-derivatized Δ⁸-THC (**4**), 303 m/z corresponding to the 231 m/z peak in the non-derivatized Δ⁸-THC (**4**), 265 m/z corresponding to the 193 m/z peak in the non-derivatized Δ⁸-THC (**4**).

6. ^{1}H and ^{13}C spectra of representative compounds.

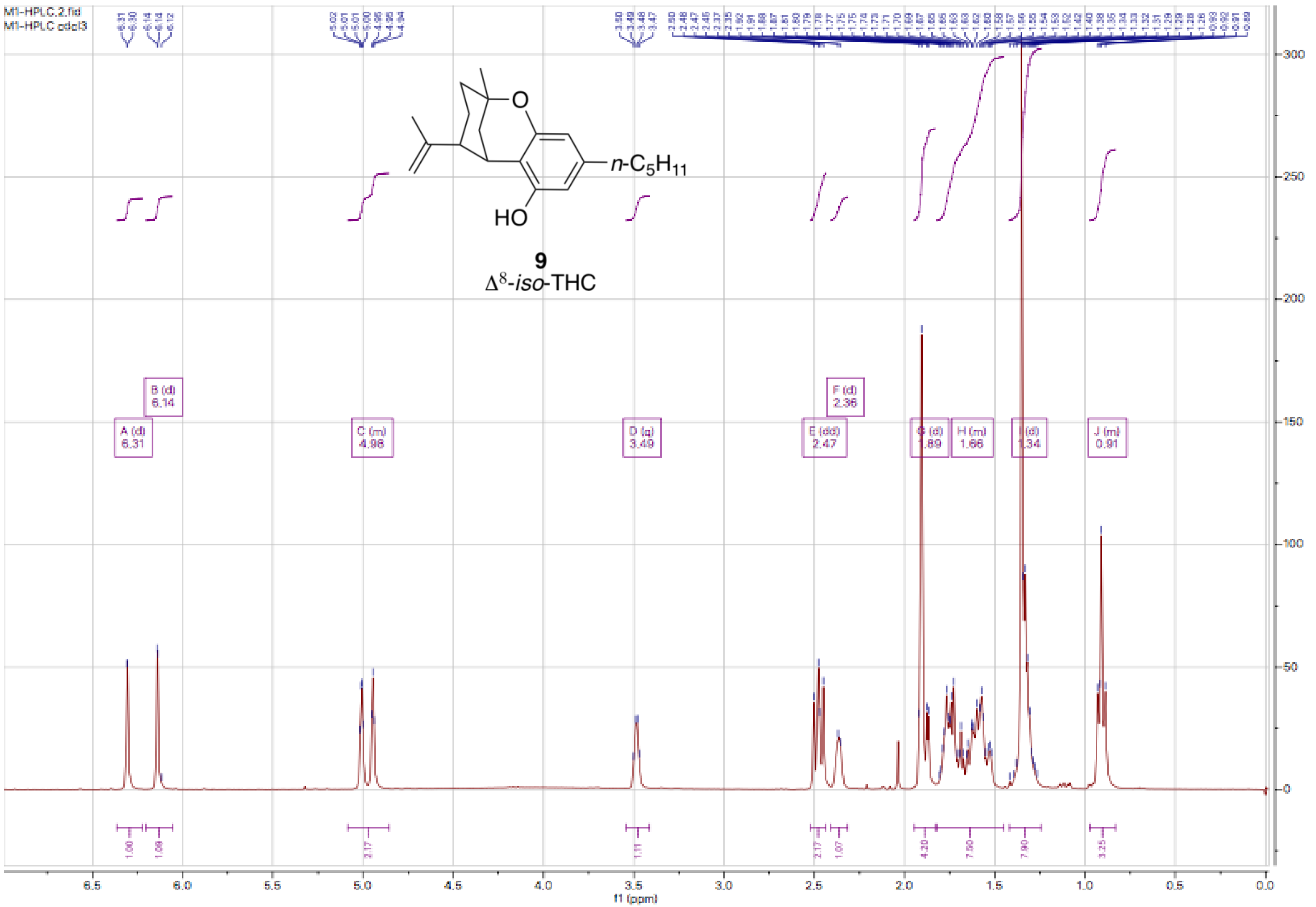

Figure 6.1. ^{1}H-NMR spectrum (300 MHz, $CDCl_3$) of Δ^8-*iso*-THC (**9**).

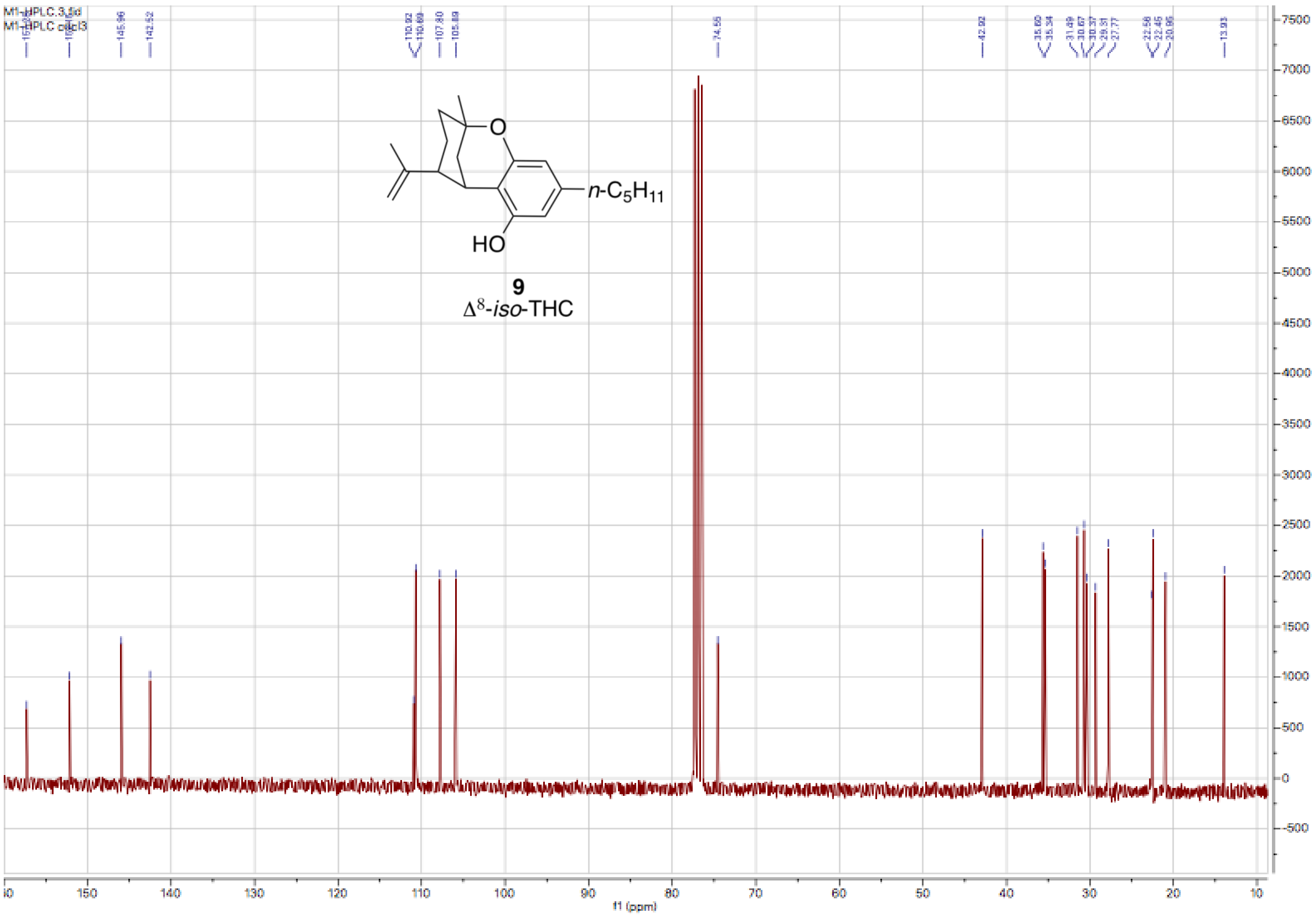

Figure 6.2. ^{13}C-NMR spectrum (75 MHz, $CDCl_3$) of Δ^8-*iso*-THC (**9**).

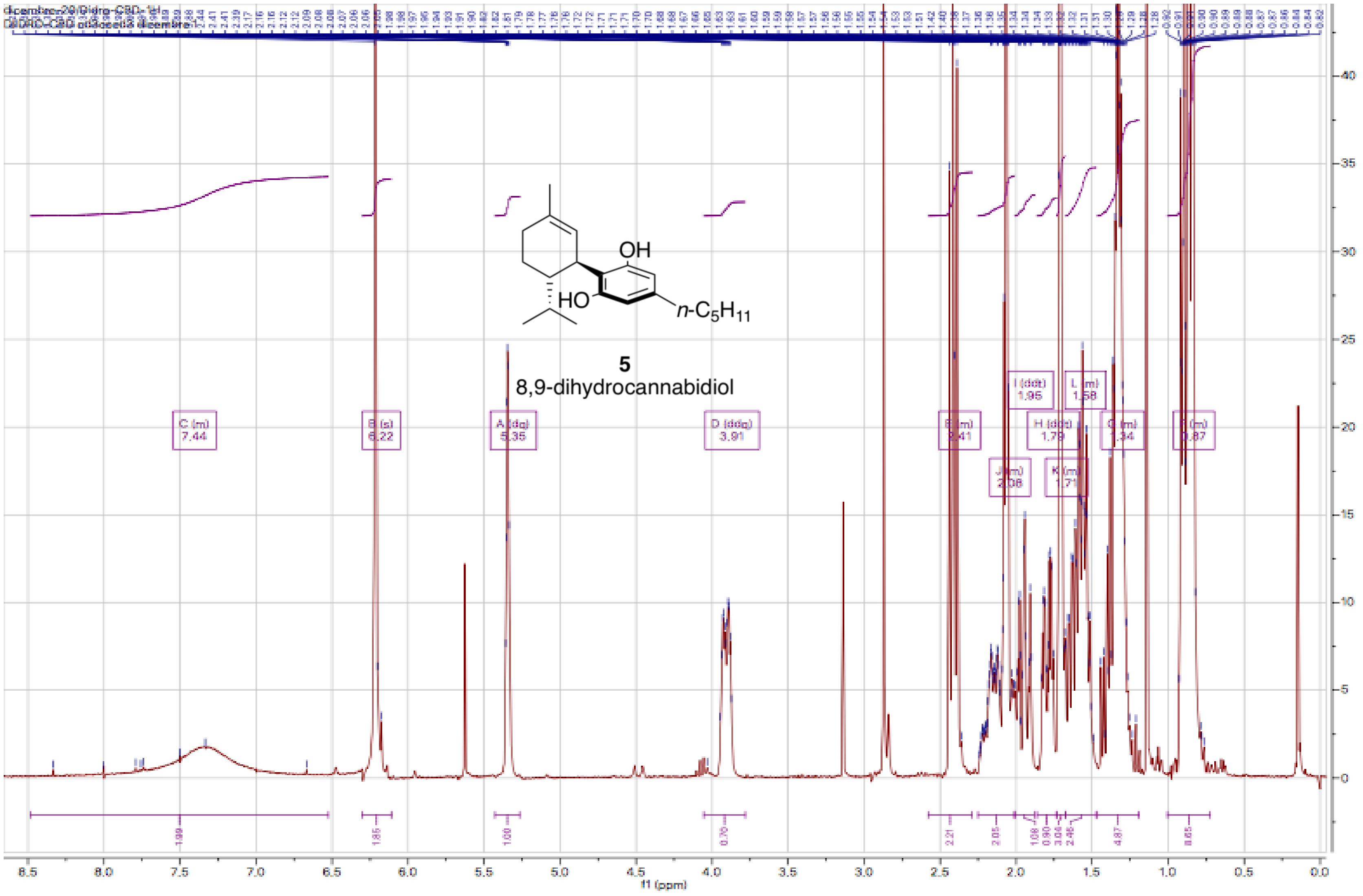

Figure 6.3. ^{1}H-NMR spectrum (300 MHz, Acetone-d_6) of DHD (**5**).

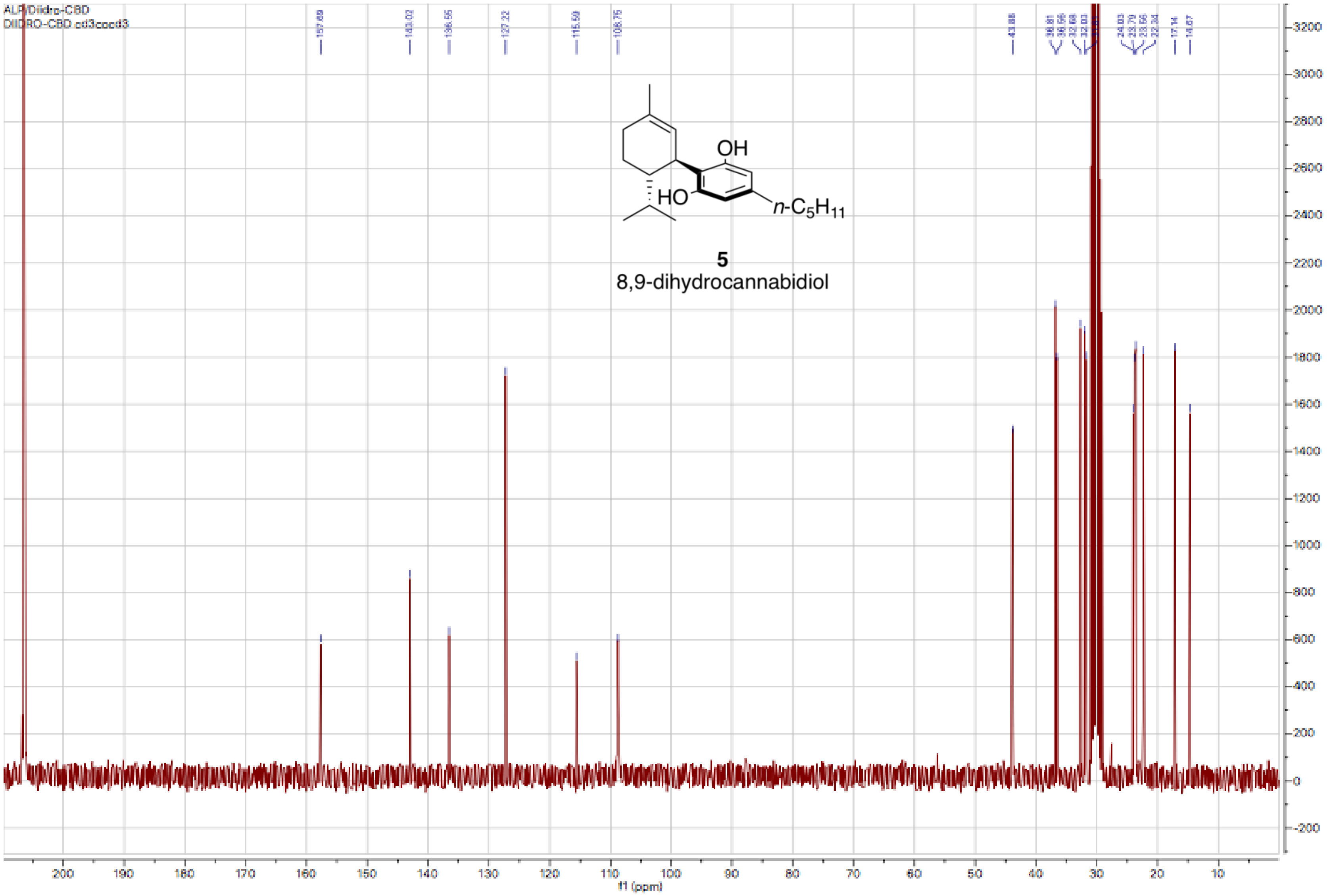

Figure 6.4. ^{13}C-NMR spectrum (75 MHz, Acetone-d_6) of DHD (**5**).

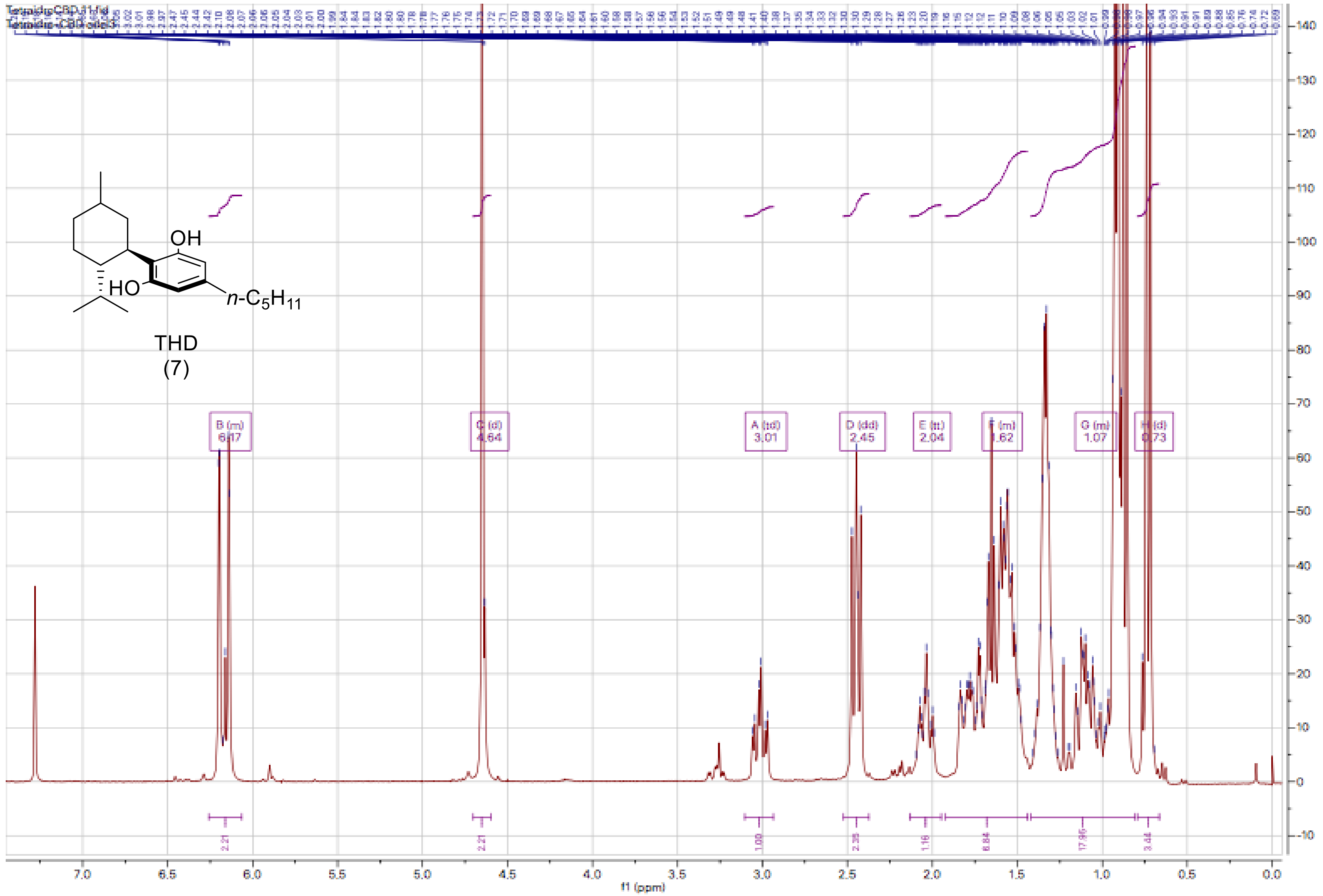

Figure 6.5. ^{1}H-NMR spectrum (300 MHz, $CDCl_3$) of THD (**7**).

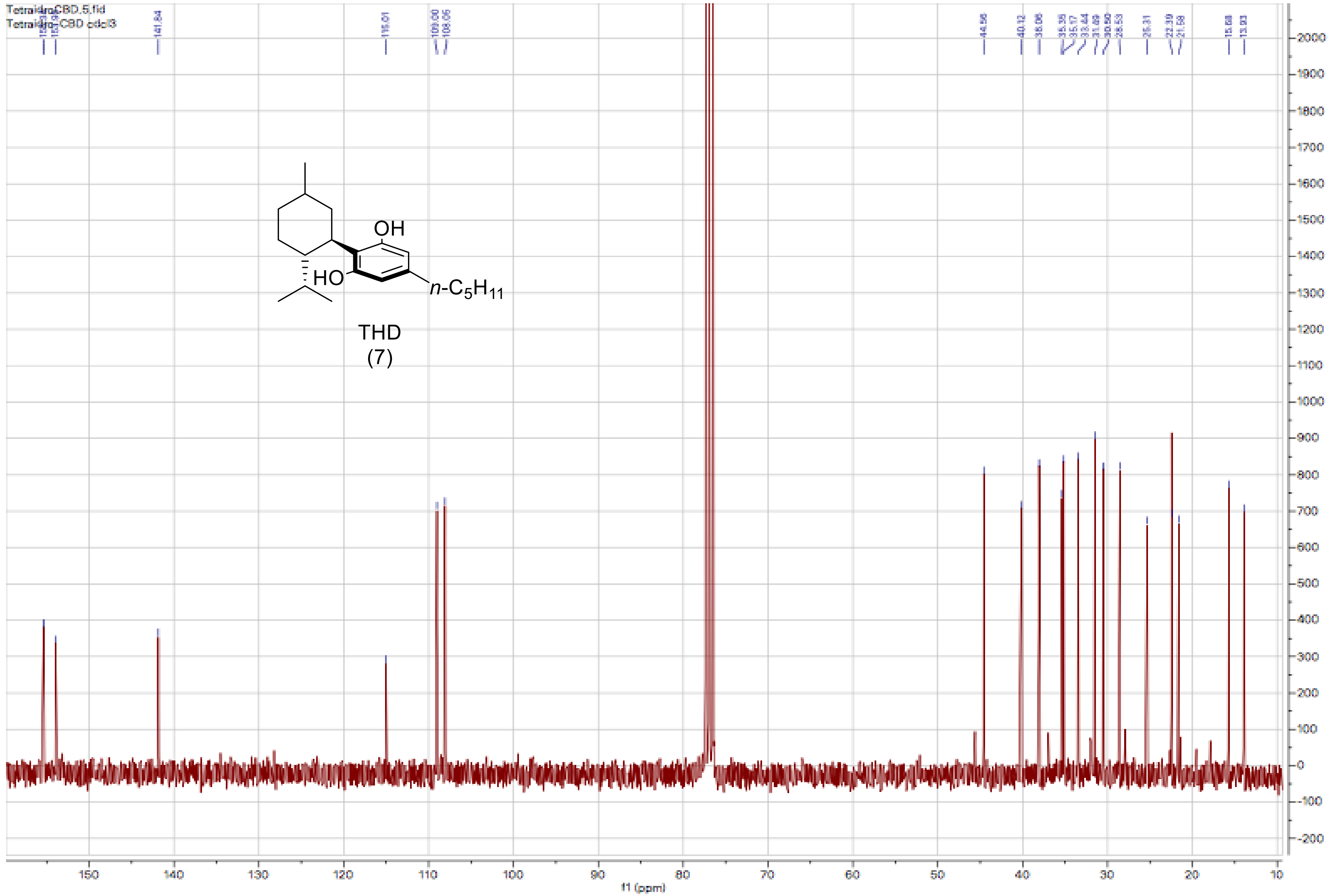

Figure 6.6. ^{13}C-NMR spectrum (75 MHz, $CDCl_3$) of THD (**7**).

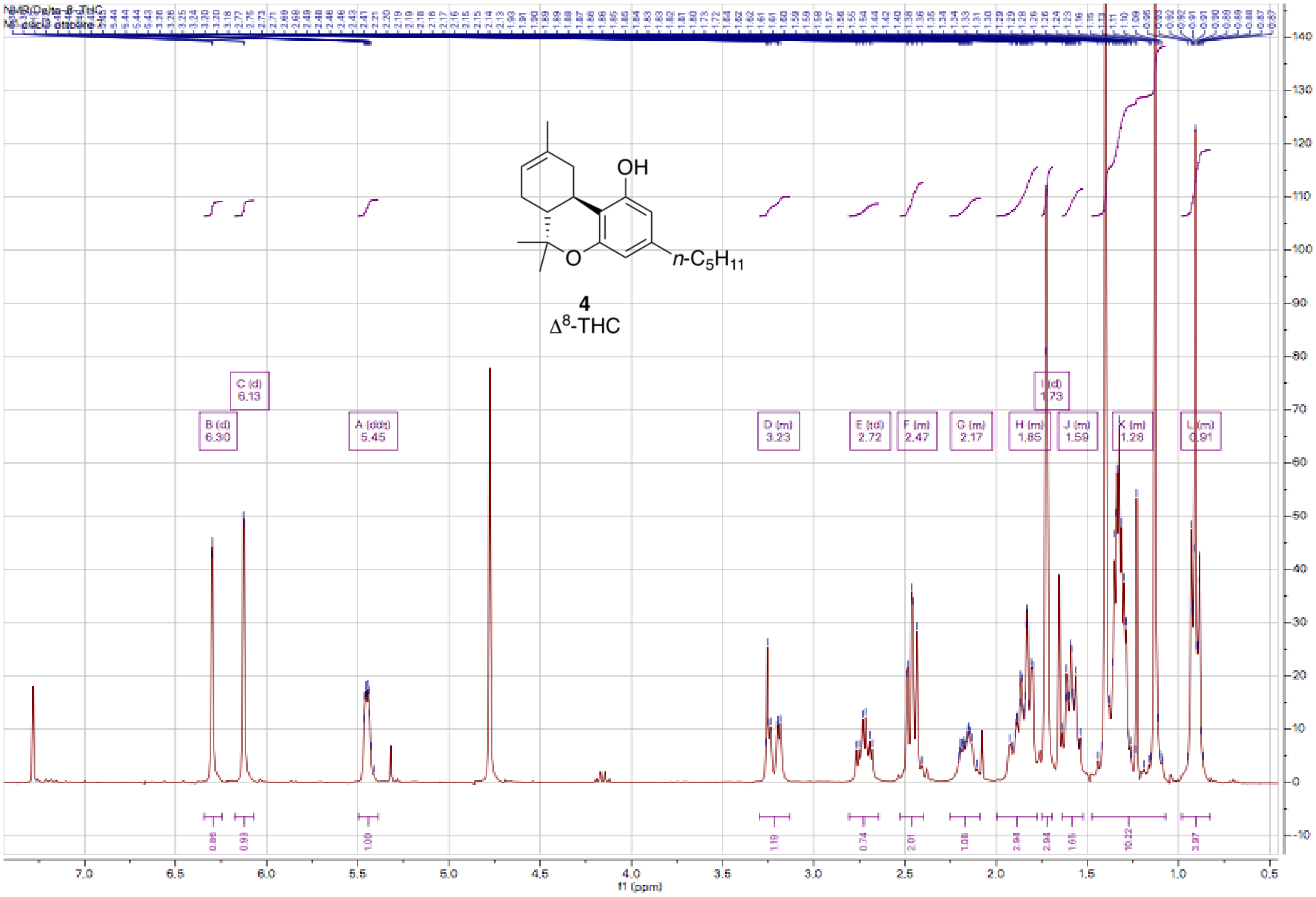

Figure 6.7. ^{1}H-NMR spectrum (300 MHz, $CDCl_3$) of Δ^8-THC (**4**).

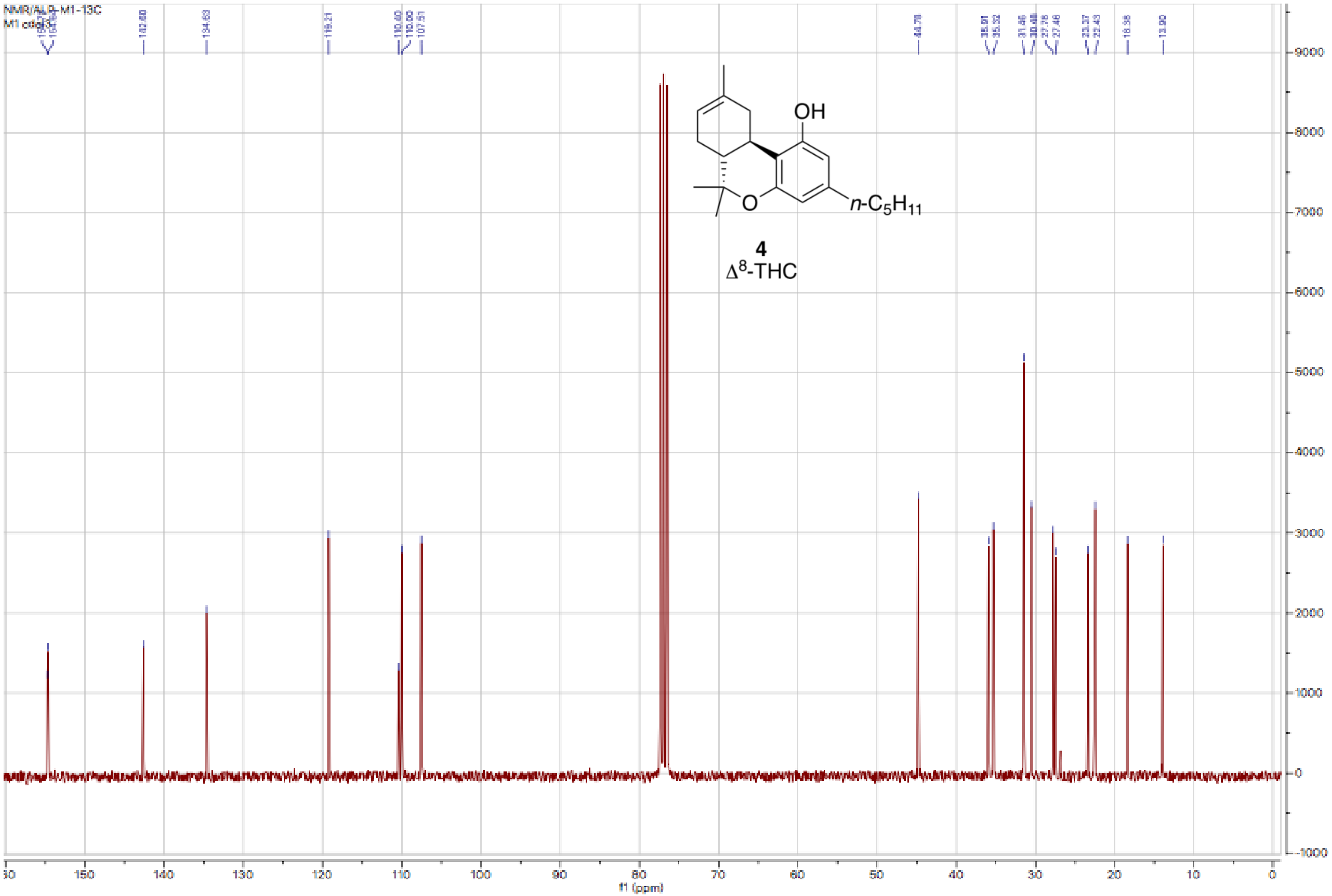

Figure 6.8. ^{13}C-NMR spectrum (75 MHz, $CDCl_3$) of Δ^8-THC (**4**).

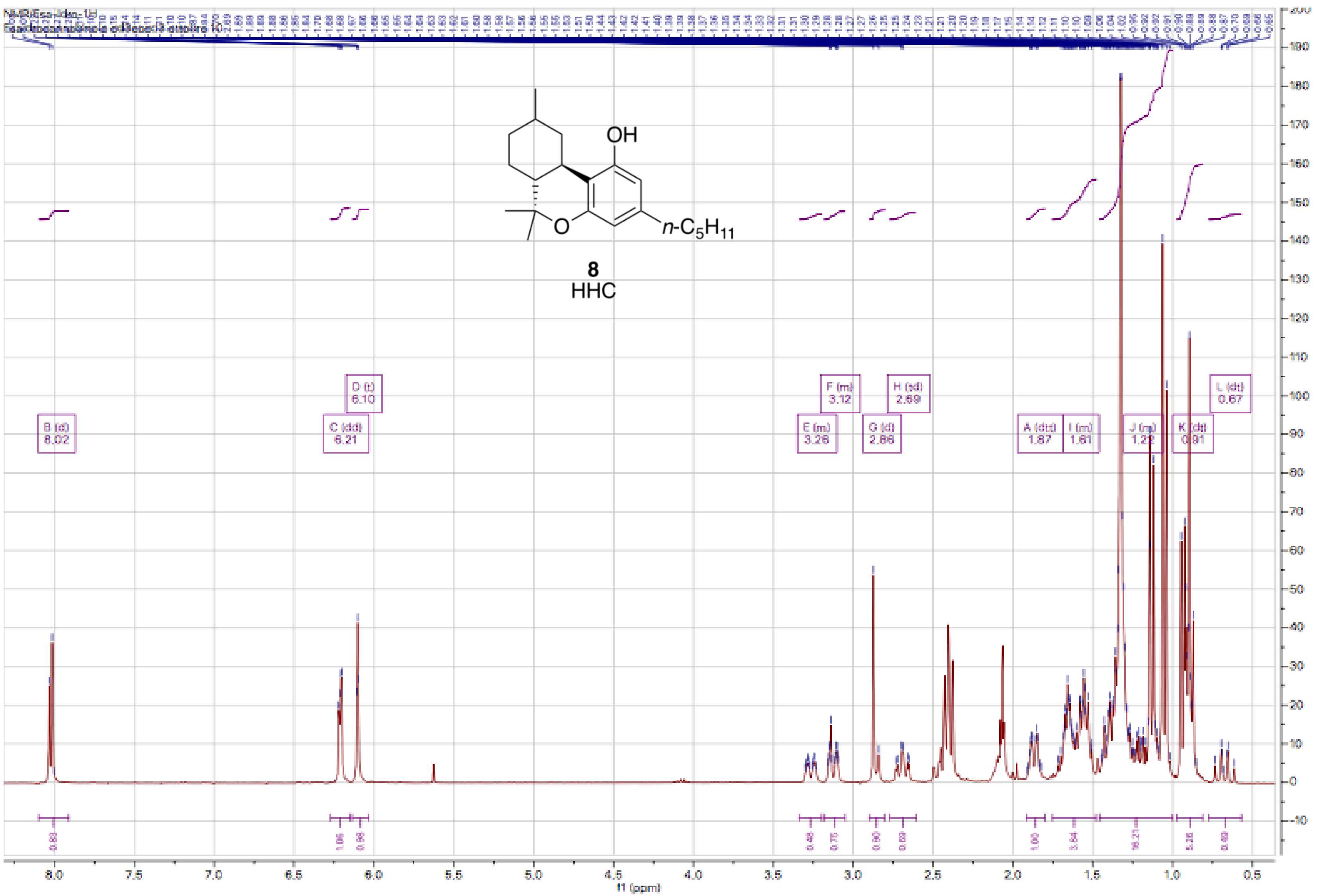

Figure 6.9. ^{1}H-NMR spectrum (300 MHz, Acetone-d_6) of HHC (**8**).

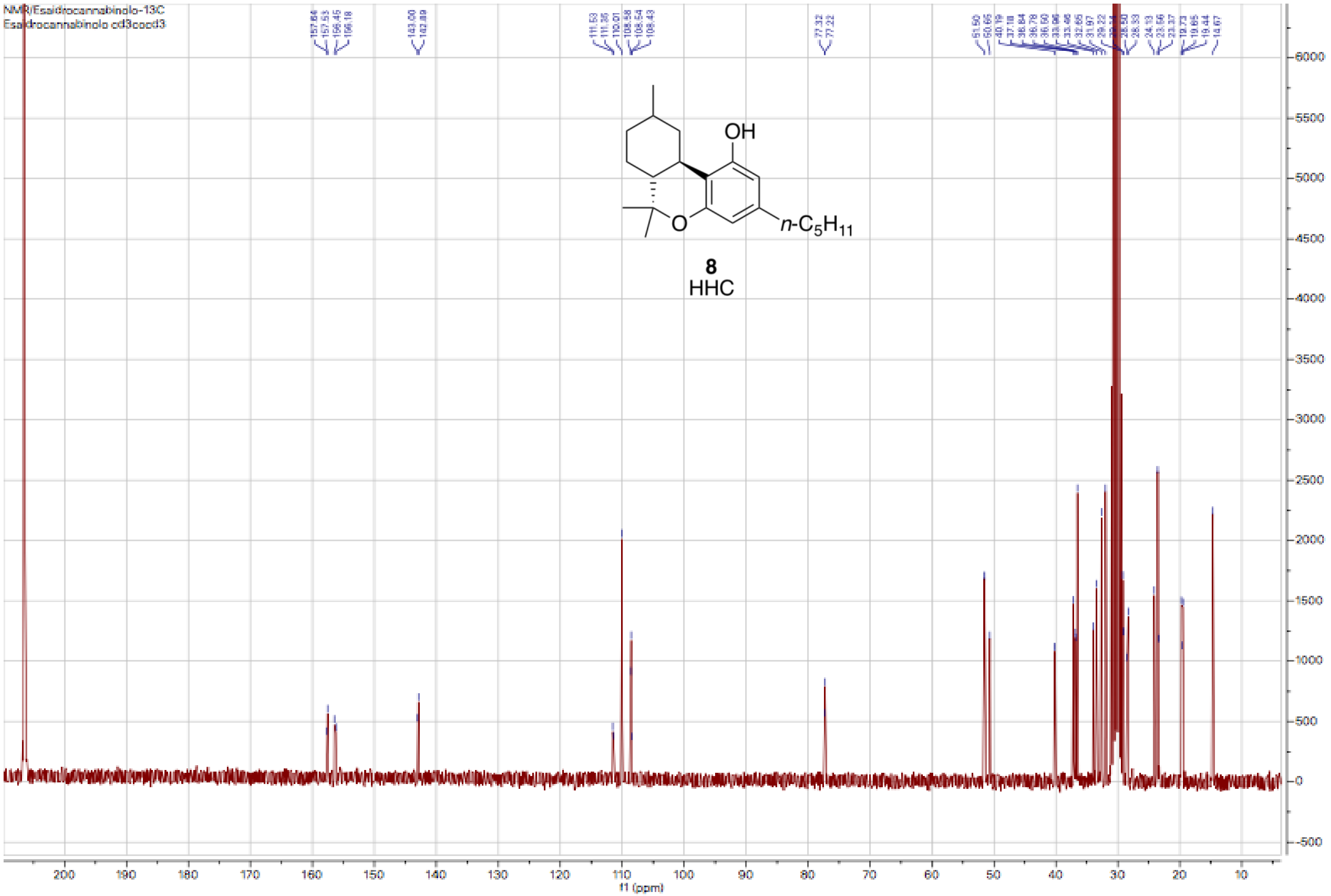

Figure 6.10. ^{13}C-NMR spectrum (75 MHz, Acetone-d_6) of HHC (**8**).

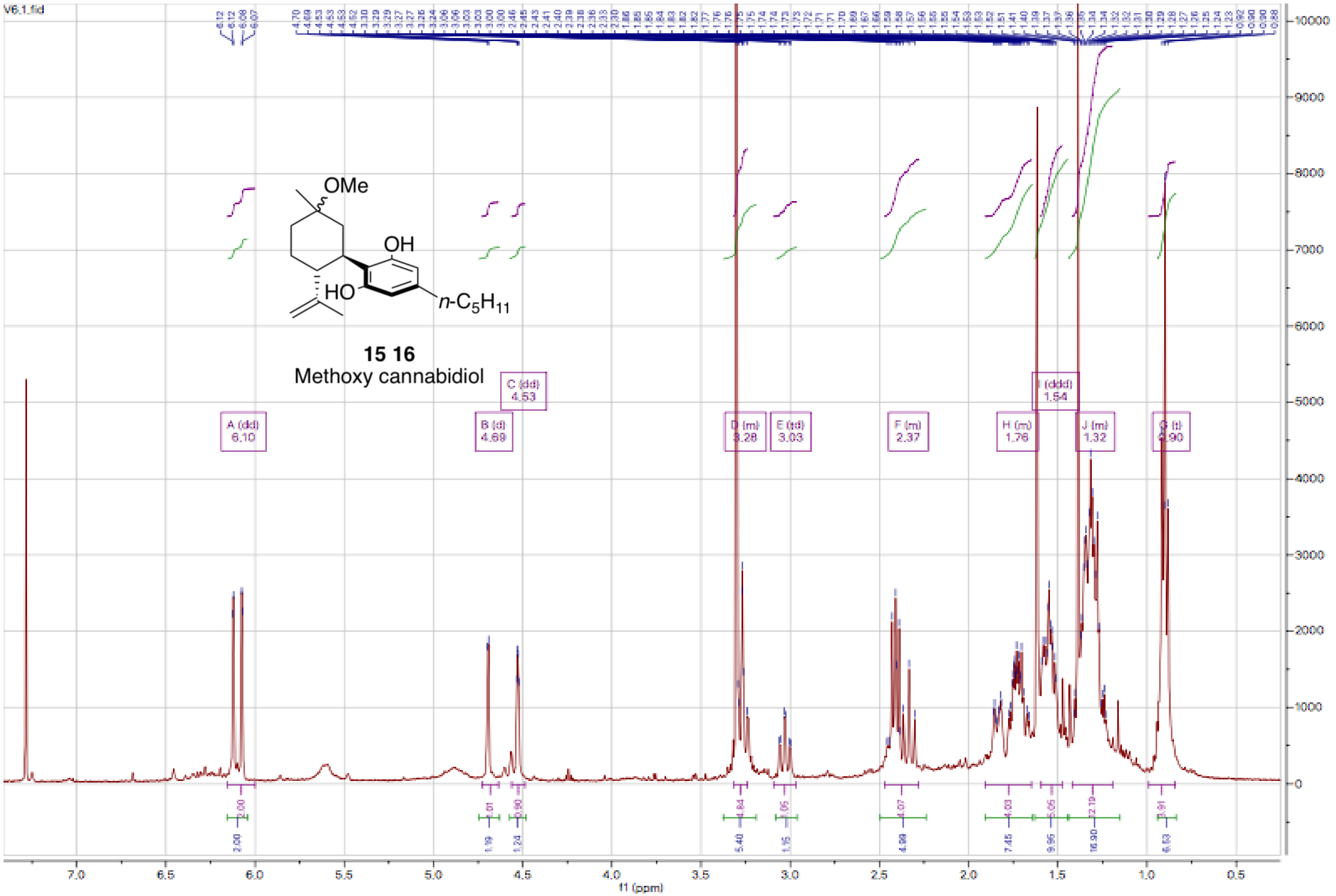

Figure 6.11. ^{1}H-NMR spectrum (400 MHz, $CDCl_3$) of Methoxy-CBD (**15-16**).

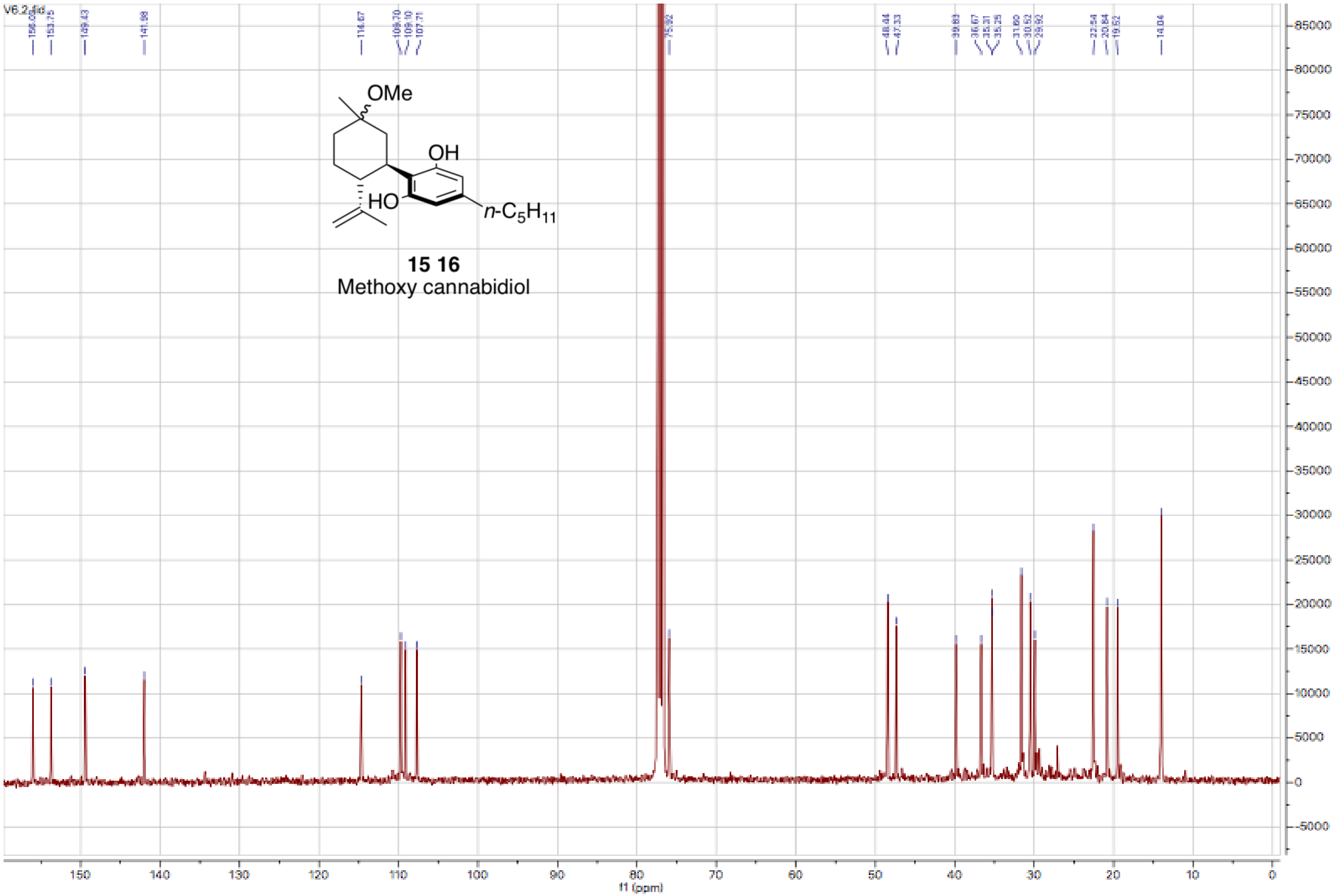

Figure 6.12. ^{13}C-NMR spectrum (100 MHz, $CDCl_3$) of α,β Methoxy-CBD (**15-16**).

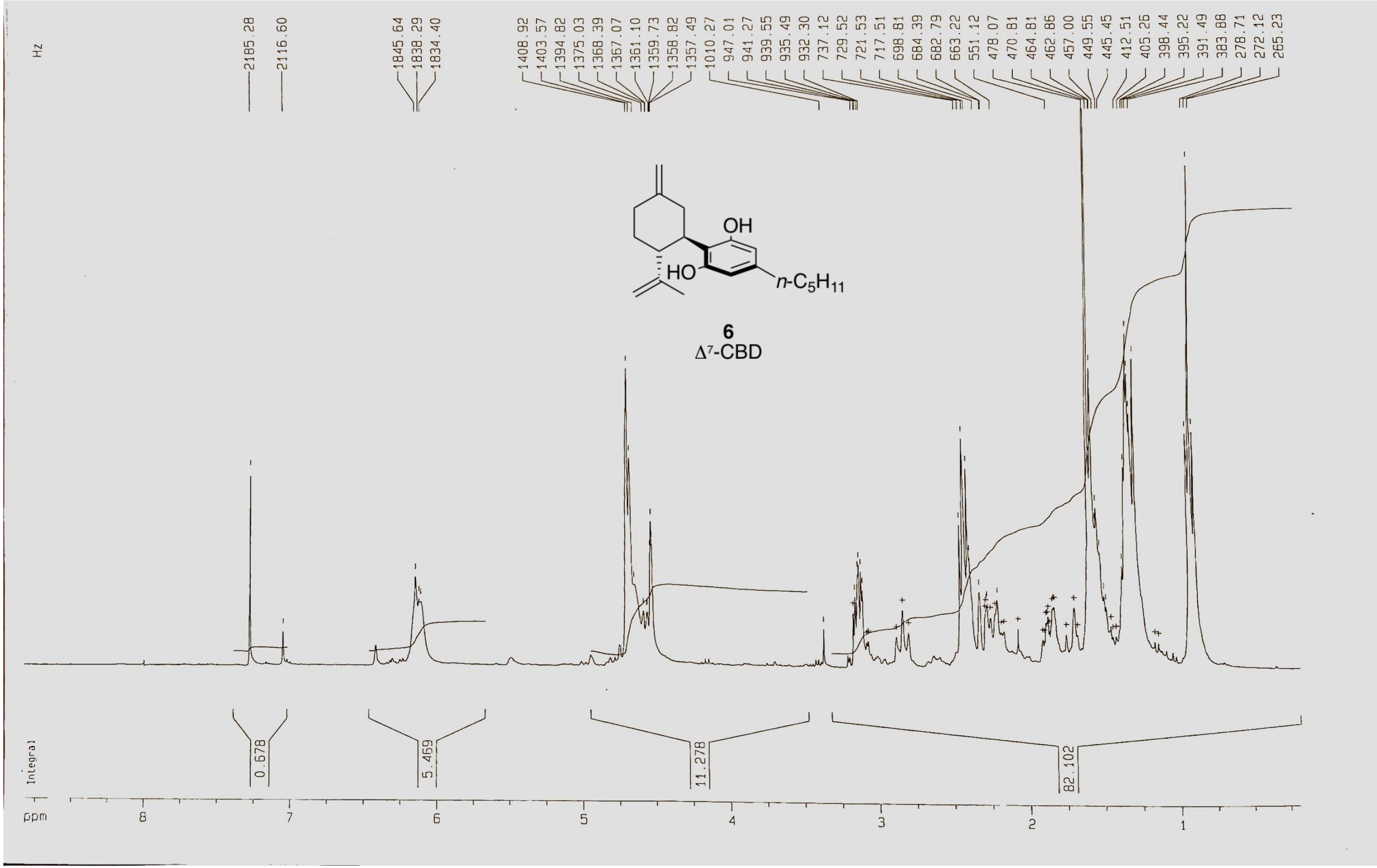

Figure 6.13. ^{1}H-NMR spectrum (300 MHz, $CDCl_3$) of Δ^{7} – CBD (**6**).

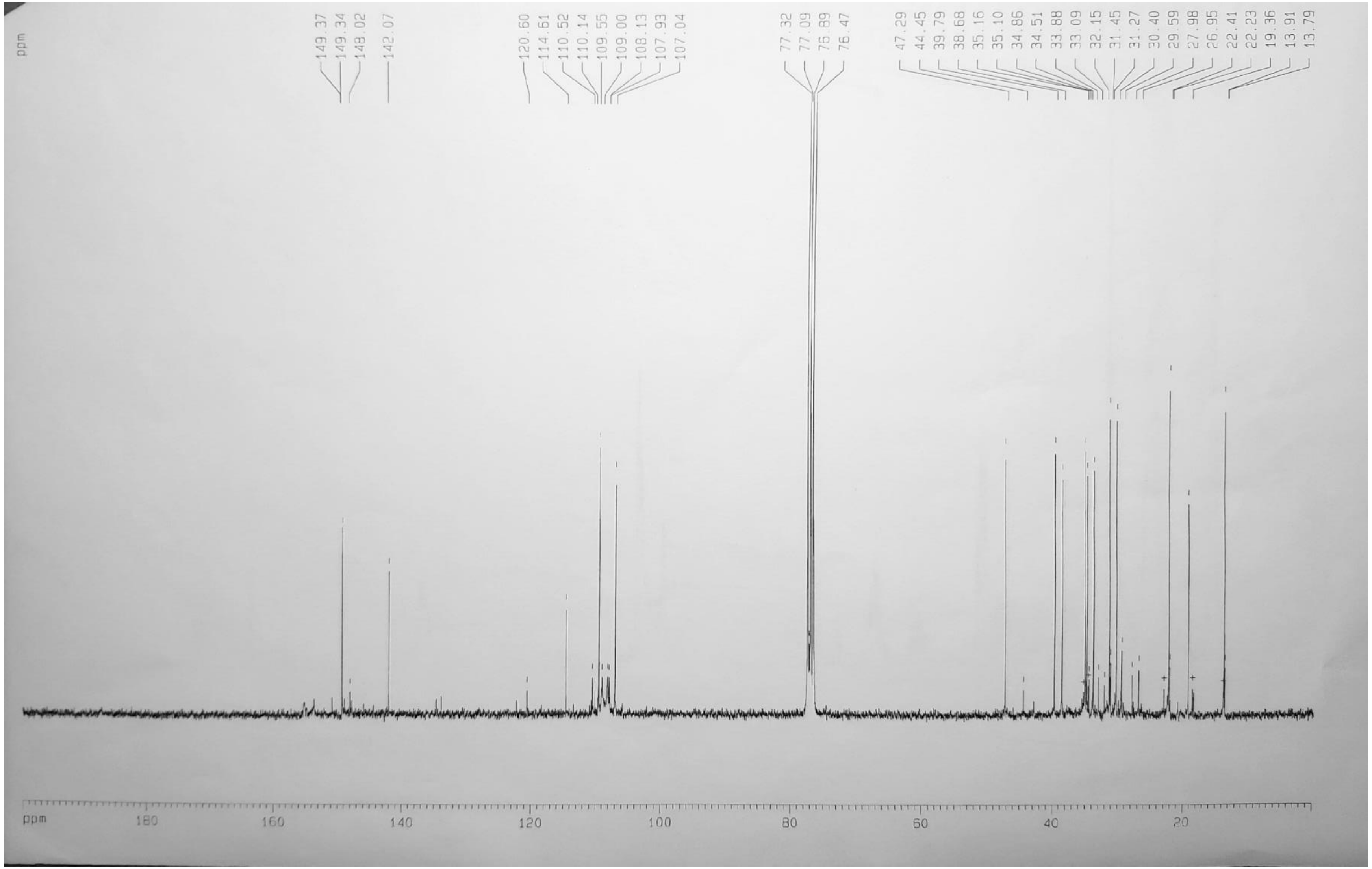

Figure 6.14. ^{13}C-NMR spectrum (75 MHz, $CDCl_3$) of Δ^7 – CBD (**6**).

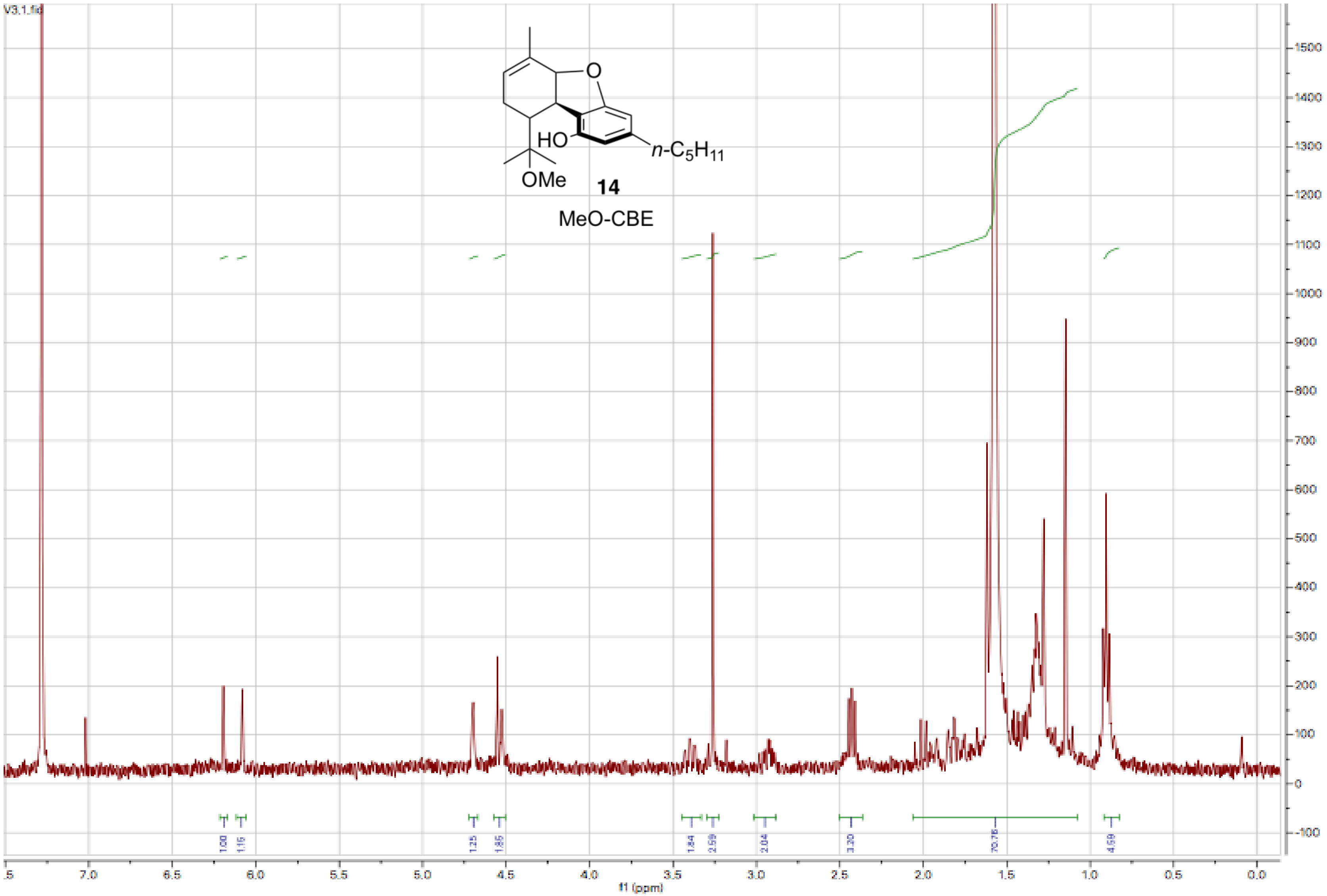

Figure 6.15. ¹H-NMR spectrum (400 MHz, $CDCl_3$) of MeO-CBE (**14**).

7. Irradiation of CBD (2) at 310 nm.

In all cases, the conversion of CBD in follows the same distribution products as observed irradiating at 254 nm. The conversion is <45%, comparing the same irradiation time. Below, the results obtained for each solvent considered are reported.

7.1 Irradiation of CBD (2) in MeCN at 310 nm

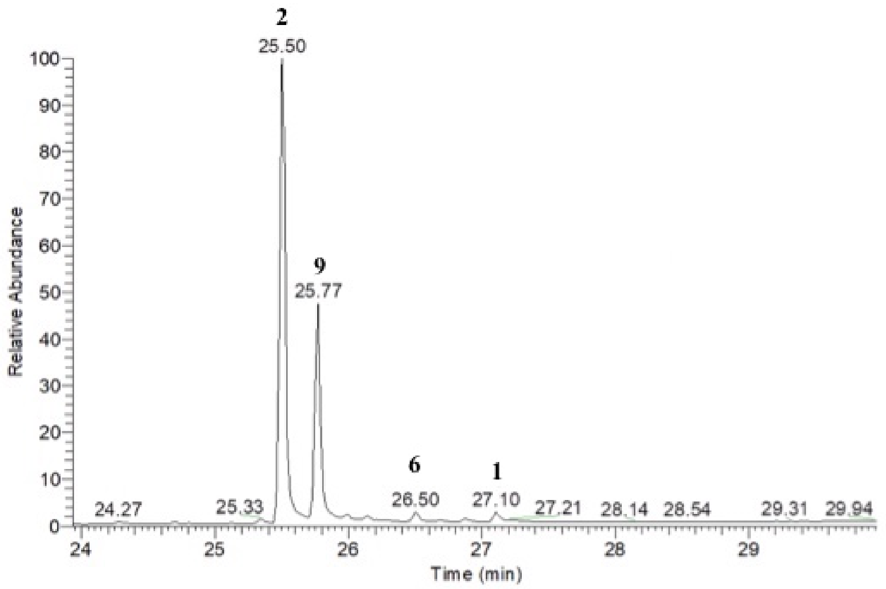

Fig 7.1. Effect of irradiation of CBD (**2**) in MeCN at 310 nm for 50 min.

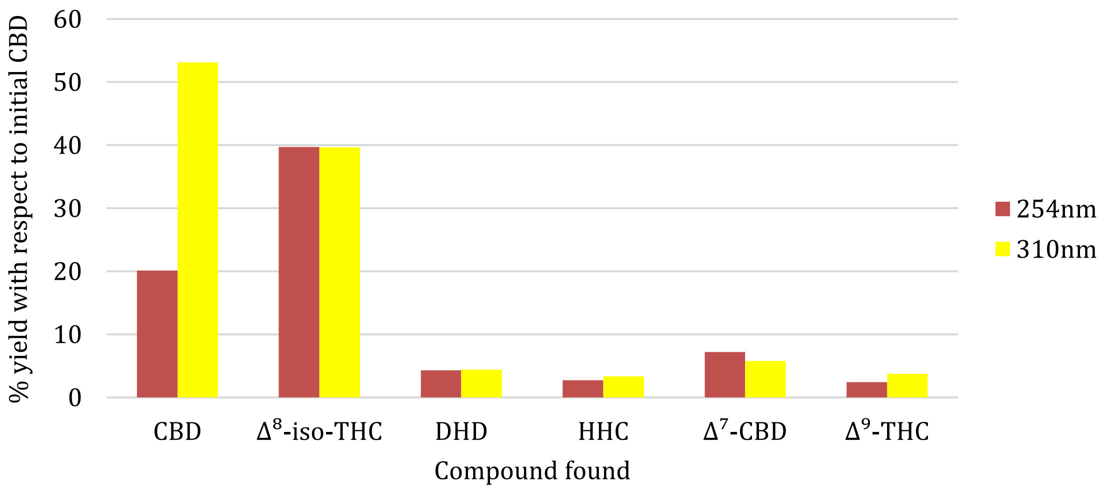

Fig. 7.2 Comparison of product distribution at 250 nm and 310 nm (MeCN, 50 min irradiation time). CBD data refers to the amount of compound left unchanged in the solution after the irradiation. When CBD was irradiated in degassed 4:1 MeCN:acetone, we obtained the following outcome: CBD (**2**) consumption 50%, Δ^8-*iso*-THC (**9**) 37% yield, Δ^9- THC (**1**) 3% yield.

7.2 Irradiation of CBD (2) in MeOH at 310 nm.

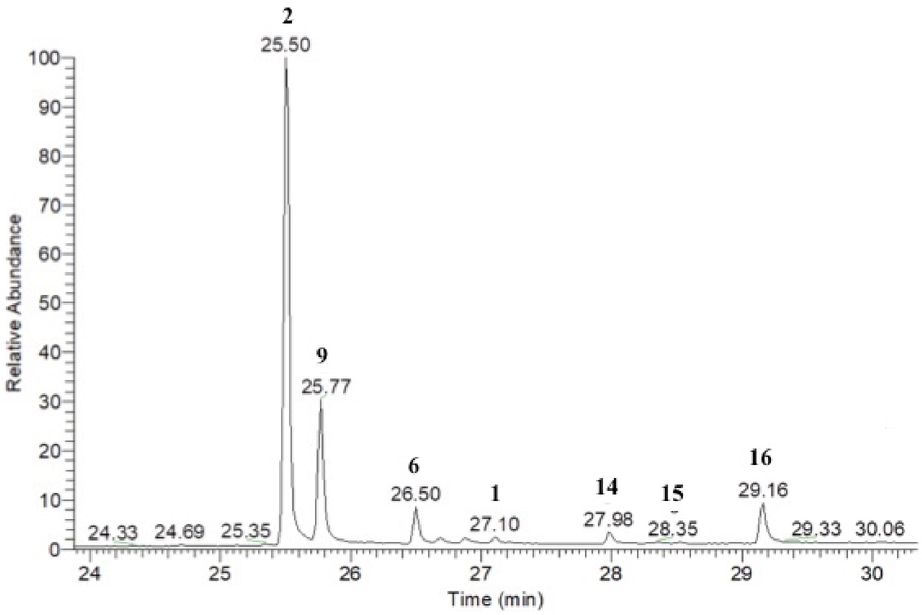

Fig 7.3. Effect of irradiation of CBD (**2**) in MeOH at 310 nm for 50 min.

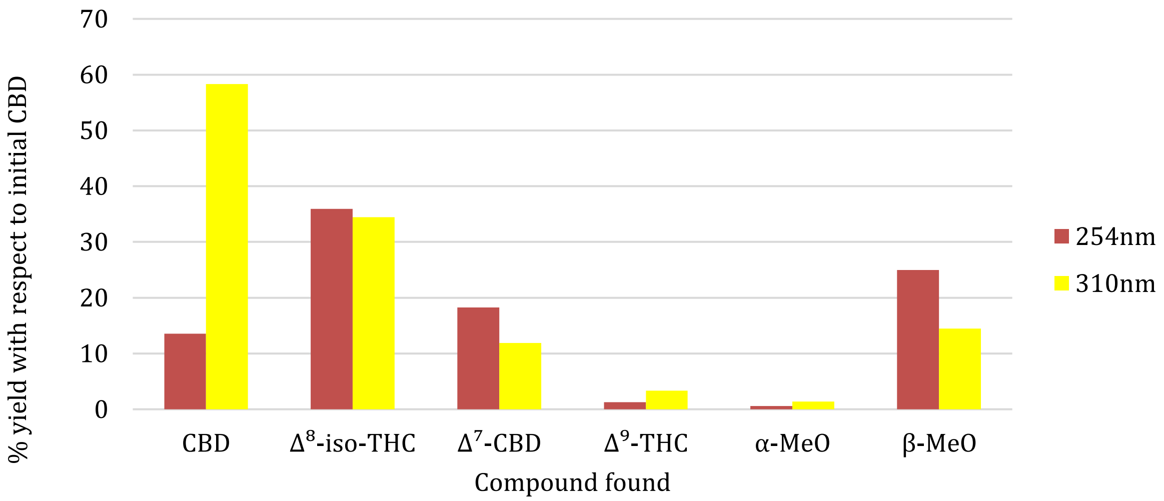

Fig. 7.4 Comparison of product distribution at 250 nm and 310 nm (MeOH, 50 min irradiation time). CBD data refers to the amount of compound left unchanged in the solution after the irradiation.

7.3 Irradiation of CBD (2) in *n*-hexane at 310 nm.

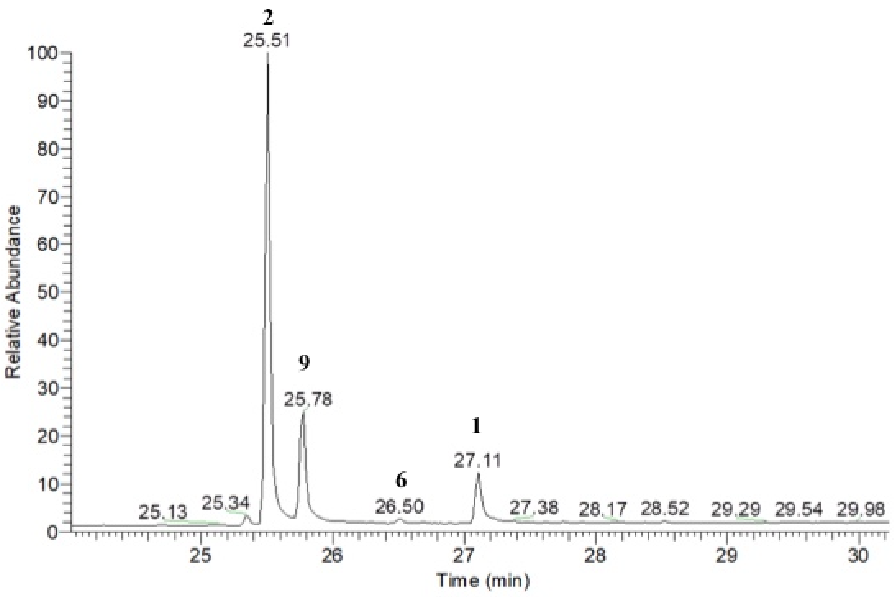

Fig 7.5. Effect of irradiation of CBD (**2**) in *n*-hexane at 310 nm for 50 min.

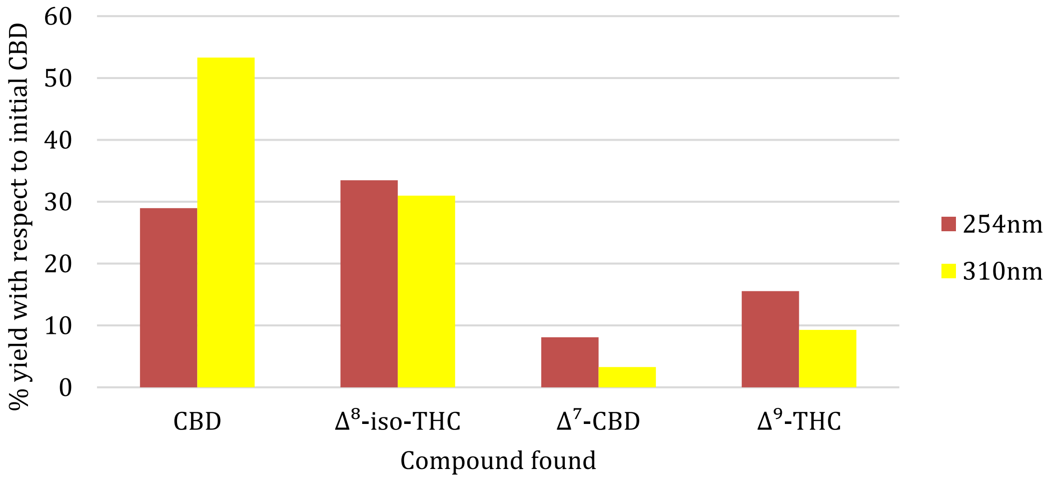

Fig. 7.6. Comparison of product distribution at 250 nm and 310 nm (*n*-hexane, 50 min irradiation time). CBD data refers to the amount of compound left unchanged in the solution after the irradiation.

8. Formation of dimeric products: UHPLC-HESI-MS analysis.

8.1 UHPLC conditions.

Analysis were performed with a Thermo Scientific LTQ-XL spectrometer (LTQ-XL, linear ionic trap mass spectrometer) coupled with a Jasco XLC UHPLC system. Restek Raptor LC-column was used (10 cm x 2.1 mm, 1.8 µm). Gradient elution was used start at 90:10 (H_2O-MeCN, 0.2% HCOOH for both), final conditions 100% MeCN (with 0.2% HCOOH) in 10 min. followed by 4 min at the same conditions for column cleanup. Column flow was 300 µL/min. Analysis were performed at 40°C.

Results obtained with positive mode ions are reported below. The results are collected using hexanes as a solvent, similar results were obtained with MeCN and MeOH as a solvent for thermal and photochemical both.

8.2 HESI-MS Settings.

Heater temperature = 70°C, I-Spray voltage = 3.5 kV, capillary temp. = 275°C, capillary lens = 24 V, Tube Lens = 60 V.

8.3 Irradiated solutions.

The samples were analyzed after irradiation at 245 nm for 50’. In the case of hexane, a waxy residue formed at the bottom of test tube. *n*-hexane or MeOH were removed with a gentle flow of nitrogen and the residue was dissolved in MeCN. The sample was then analyzed, and no previous filtration of the sample was needed. Data for the case of irradiation in hexane are reported in the figures below: figure 8.1 (a) represents the TIC, while Figure 8.1 (b) the UHPLC-MS profile of m/z range 631-627. Figures 8.1 (c) and (d) represent the MS profile of two compounds with m/z 627 at RT 11.72 min and at RT 12.01 min.

Figures 8.2 (a) and (b), corresponding respectively to the MS-MS fragmentation of peaks at RT 11.72 and 12.01 min, confirms the presence of two related covalent dimeric structures $[2M-2+H]^+$ = m/z 627. For both isomers the first fragmentation involves a dehydration (m/z = 609.5, 4-5% abundance for both isomers). Conversely, the peaks for both isomers at m/z = 571.5 correspond to the cyclic ring breaking. Base peaks for both isomers at m/z = 547.5 confirm that the two compounds are structural related.

Dimeric product account for up to the 20% of the product formed in *n*-hexane. Quantification was performed using CBD as a standard. The same compounds were found also in acetonitrile and methanol irradiation, although with a less abundance.

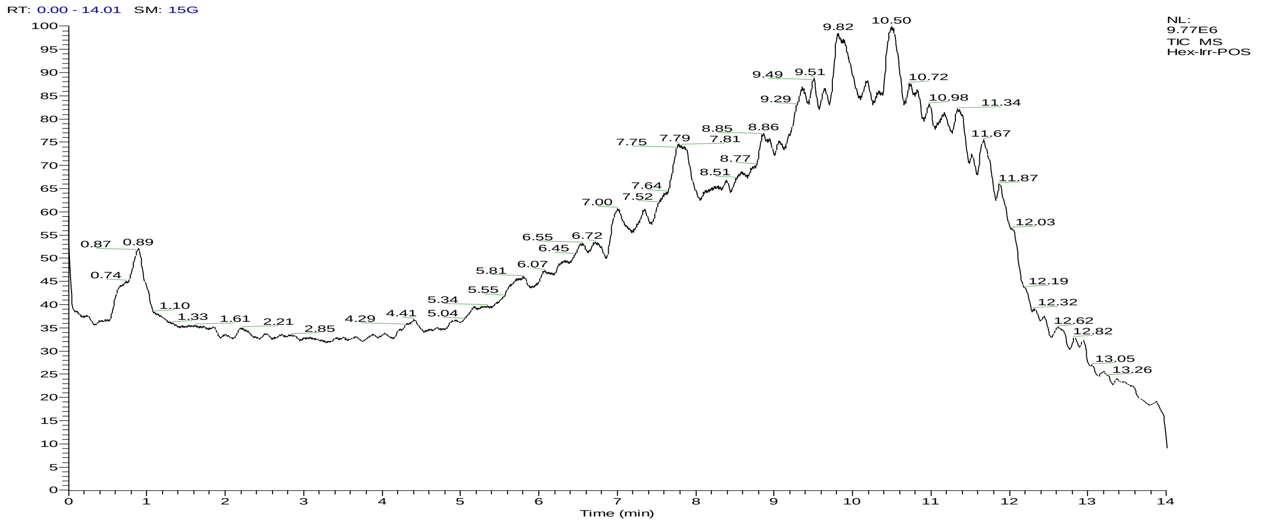
RT: 0.00 - 14.01 SM: 15G
NL: 9.77E6 TIC MS Hex-Irr-POS
Time (min)

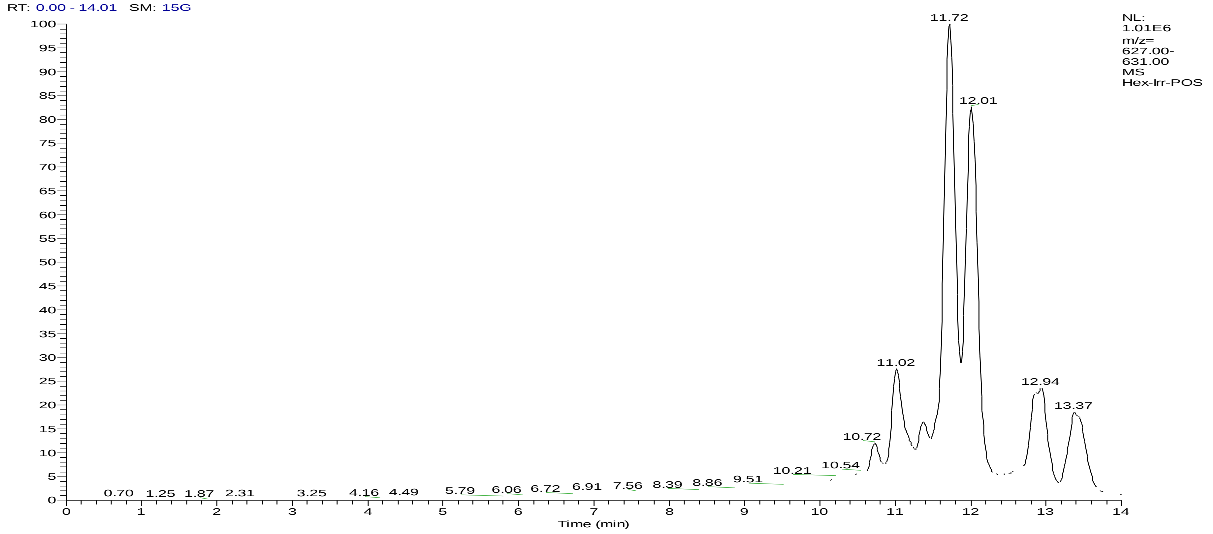
RT: 0.00 - 14.01 SM: 15G
NL: 1.01E6 m/z= 627.00-631.00 MS Hex-Irr-POS
Time (min)

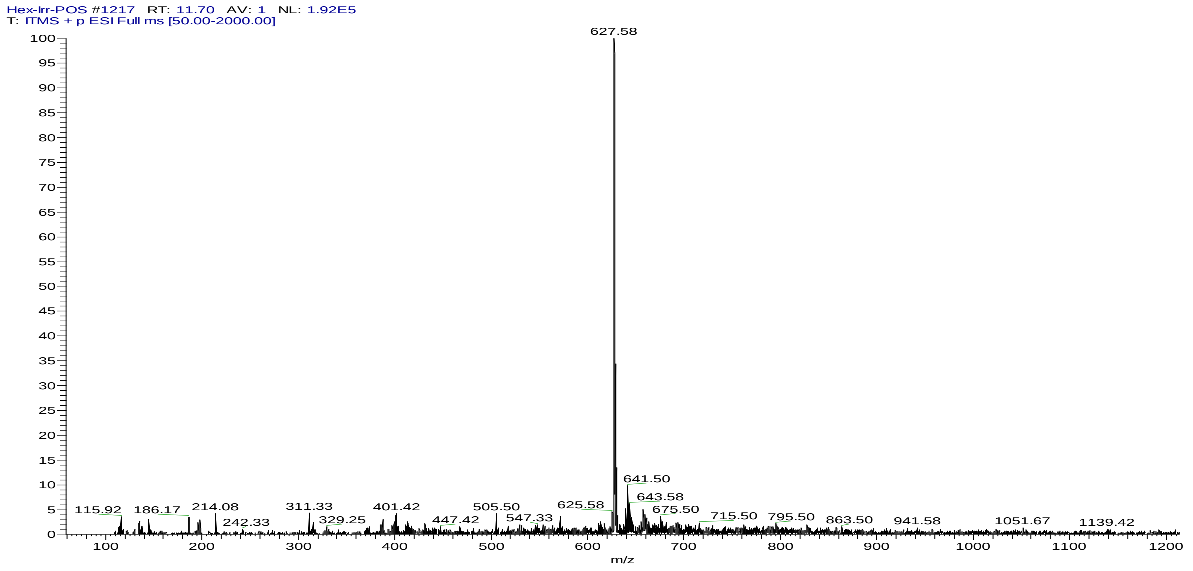
Hex-Irr-POS #1217 RT: 11.70 AV: 1 NL: 1.92E5
T: ITMS + p ESI Full ms [50.00-2000.00]
m/z

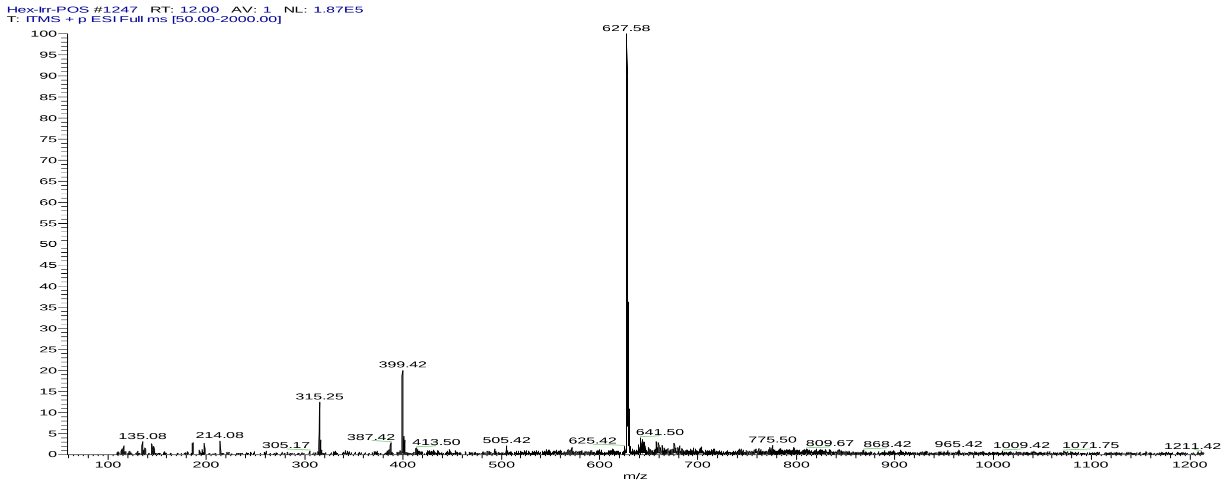

Figure 8.1. (a) UHPLC-MS profile of m/z range 50-2000 (b) UHPLC-MS profile of m/z range 631-627 (c) MS profile of m/z 627 at RT 11.72 min, corresponding to one of putative dimeric adducts. (d) MS profile of m/z 627 at RT 12.01 min, corresponding to a second putative dimeric adducts.

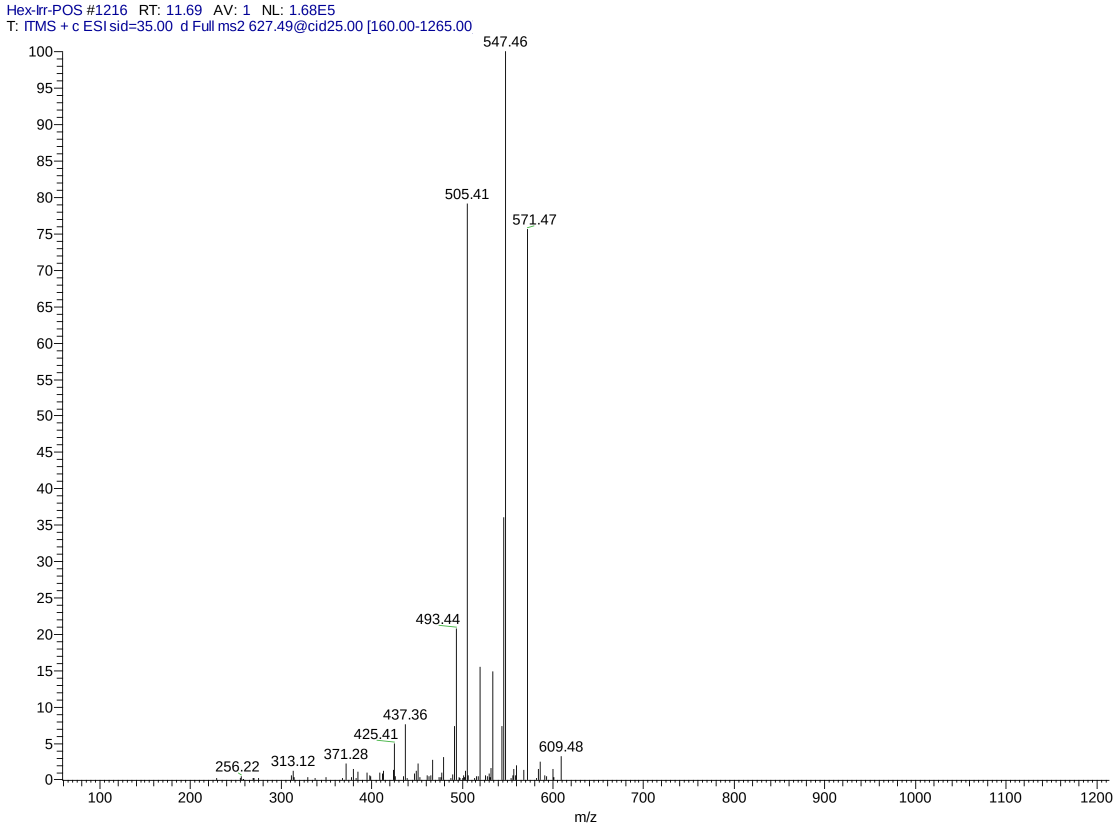

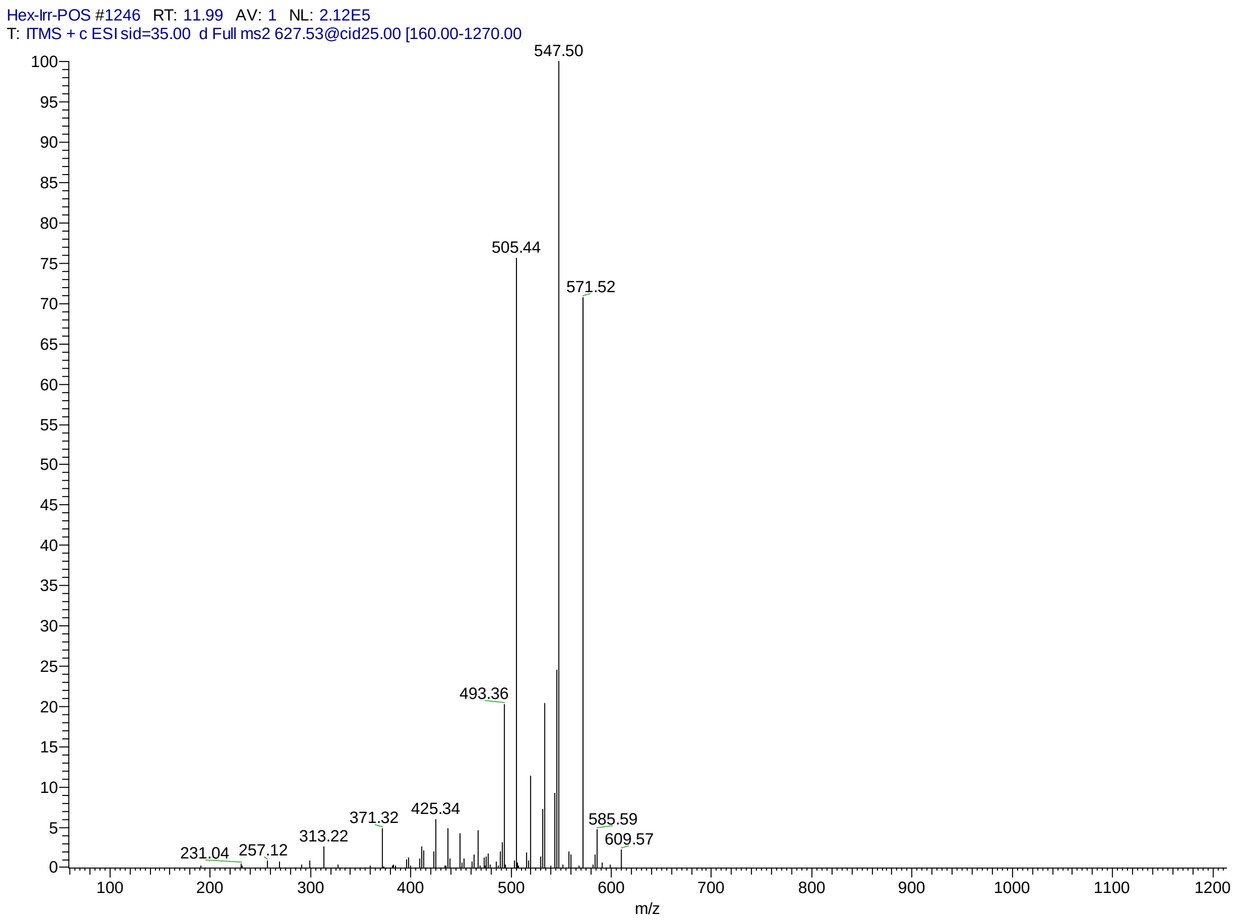

Figure 8.2 (a) MS-MS (CID = 25) of peak at 11.7 min with m/z = 627.5 (b) MS-MS (CID = 25) of peak at 12.01 min with m/z = 627.5.

8.3 Thermal degradation of CBD (2)

The sample representing the results obtained by storing a solution of CBD in *n*-hexane for 16 month at room temperature (similar results were obtained with MeCN or MeOH as a solvent). The solutions were analyzed by UHPLC-MS. In the case of MeOH and *n*-hexane, after removing solvent, the residue obtained was solubilized in MeCN and analyzed as previously described. Dimeric compounds with retention times different (11.13 min) from what observed in the case of photochemical degradation were observed.

Figure 8.3 (a) represents the TIC, while Figure 8.2 (b) is MS profile of m/z 315.2 at 8.33 min, corresponding to unreacted CBD.

Figures 8.3 (c) illustrate the MS profile for m/z 627 at RT 11.13 min for the most abundant dimeric adduct and (d) the MS profile of m/z 628 at 11.13 min, corresponding to a second dimeric adduct. Peaks at 8.3-8.40 and 9.01-9.10 are relative to some impurities not related with CBD structure. Figure 8.4 represent MS-MS (CID = 25) of peak at 11.13 min with m/z = 628.

The retention time and the fragmentation of dimeric peak confirm the difference of this compounds in comparison to dimeric structures obtained with irradiation of the samples. Fragmentation of this

dimer gives immediately monomeric CBD: this statement was confirmed with a comparison of this fragmentation with pure CBD (**2**) sample MS-MS (CID = 25) fragmentation. With these evidences we can confirm the structural difference between this dimer and the dimer obtained with irradiation.

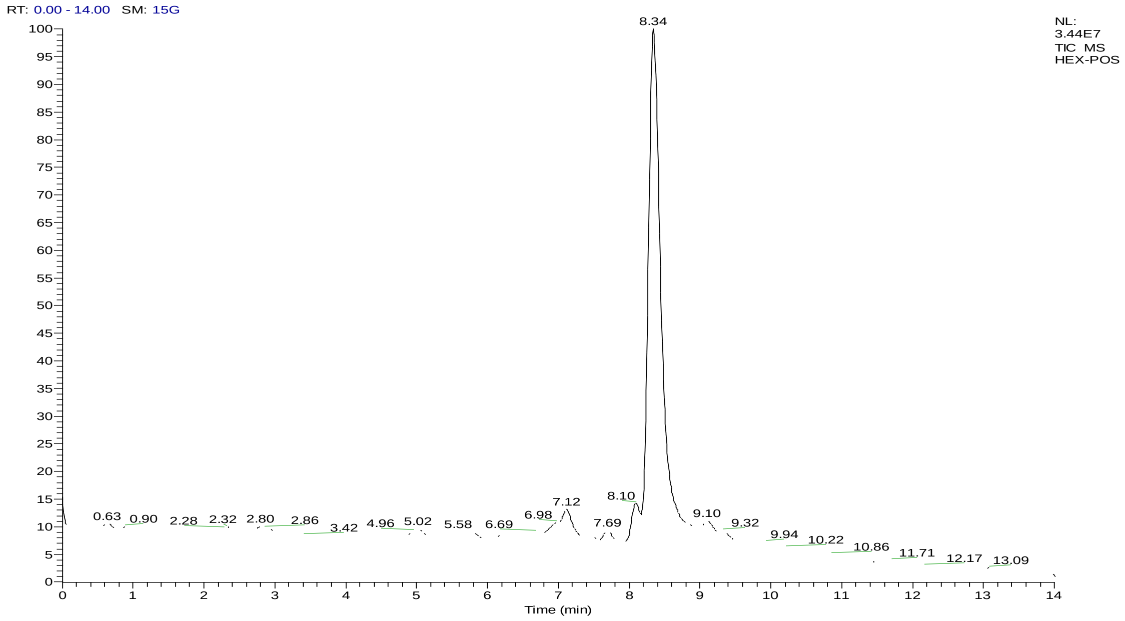

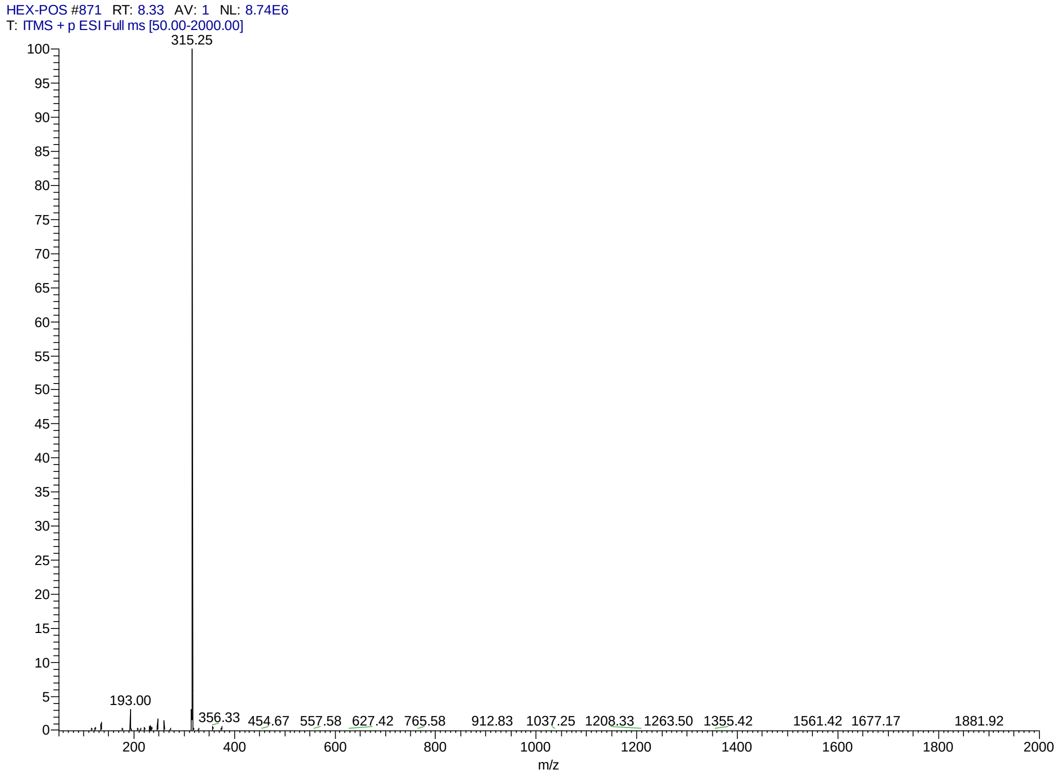

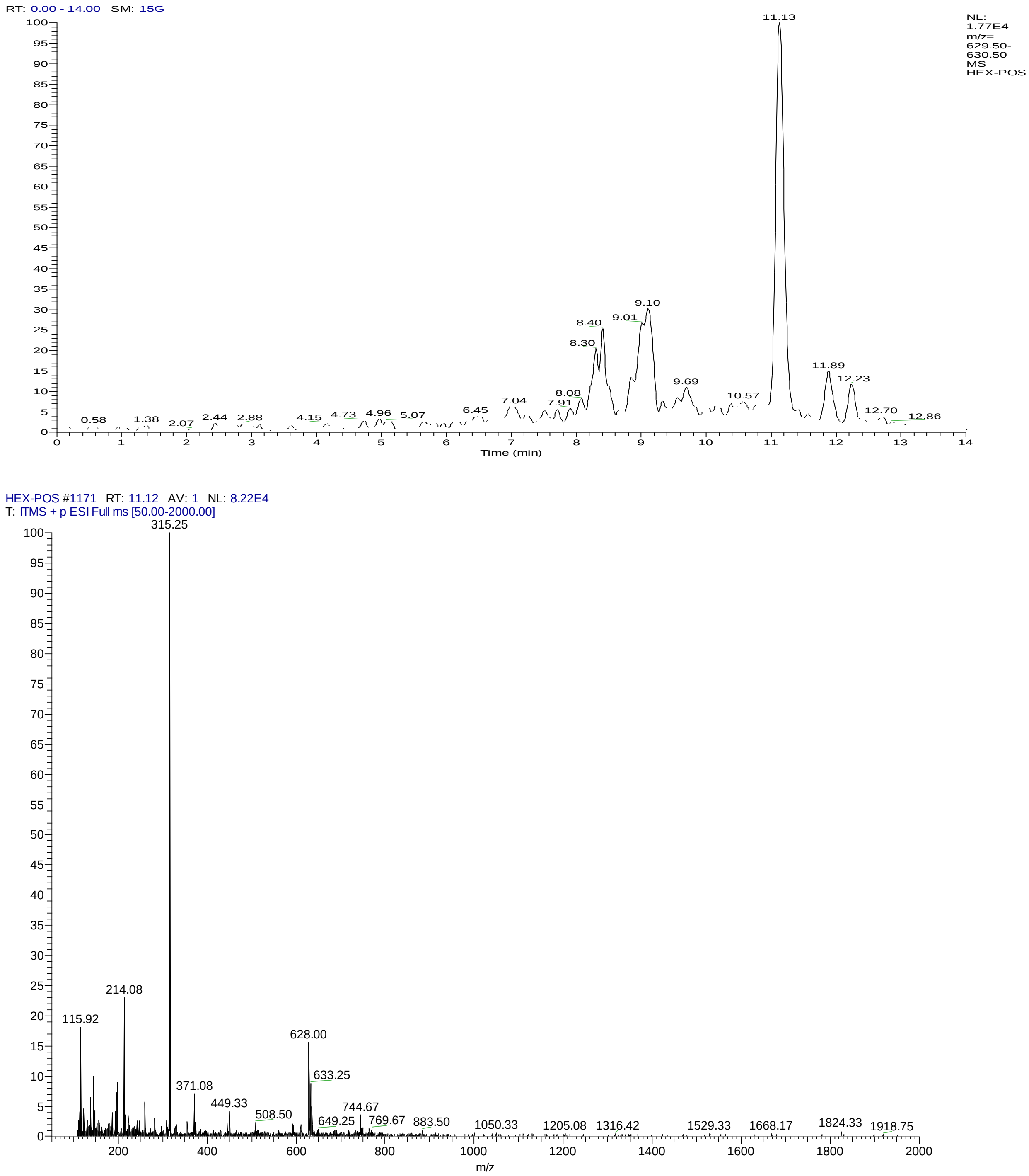

Figure 8.3. (a) UHPLC-MS profile of m/z range 50-2000; (b) MS profile of m/z 315.2 at 8.33 min, corresponding to unreacted CBD; (c) MS profile for m/z 627 at 11.13 min, corresponding to most abundant dimeric adduct; (d) MS profile of m/z 628 at 11.13 min, corresponding to most abundant dimeric adduct. Peaks at 8.3/8.40 and 9.01/9.10 are relative to some impurities not related with CBD structure.

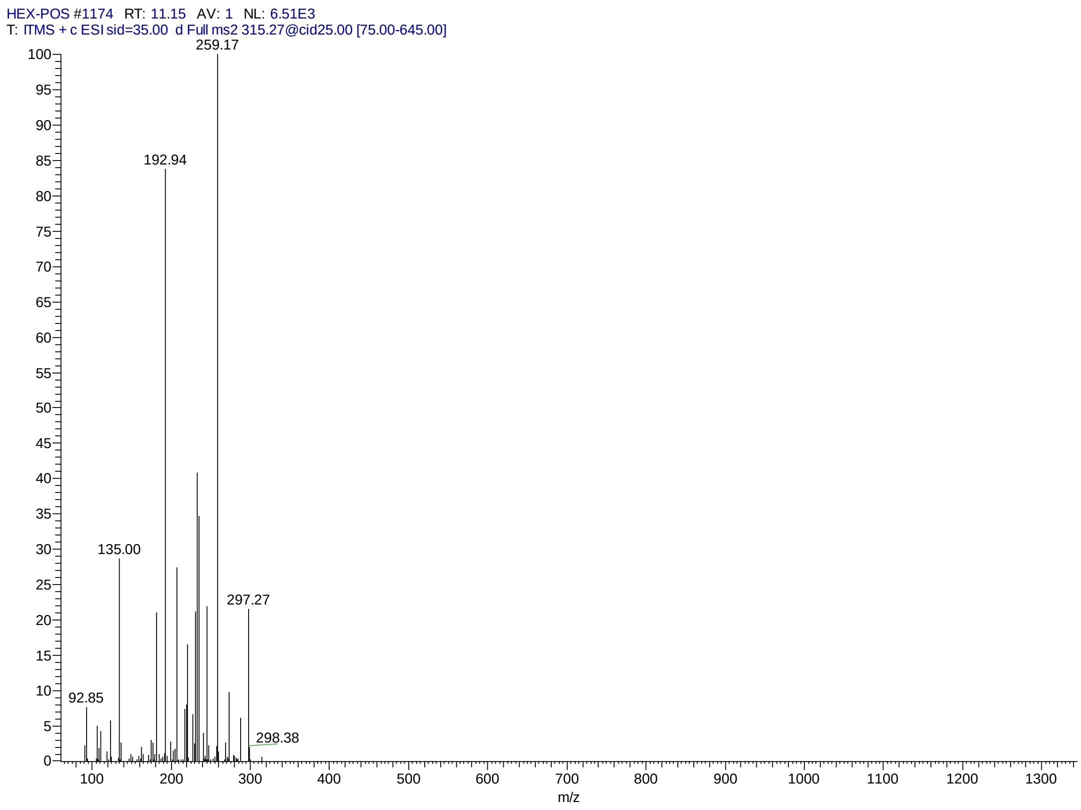

Figure 8.4 MS-MS (CID = 25) of peak at 11.13 min with m/z = 628.

9. Kinetic data for the degradation of CBD (2) in the examined solvents.

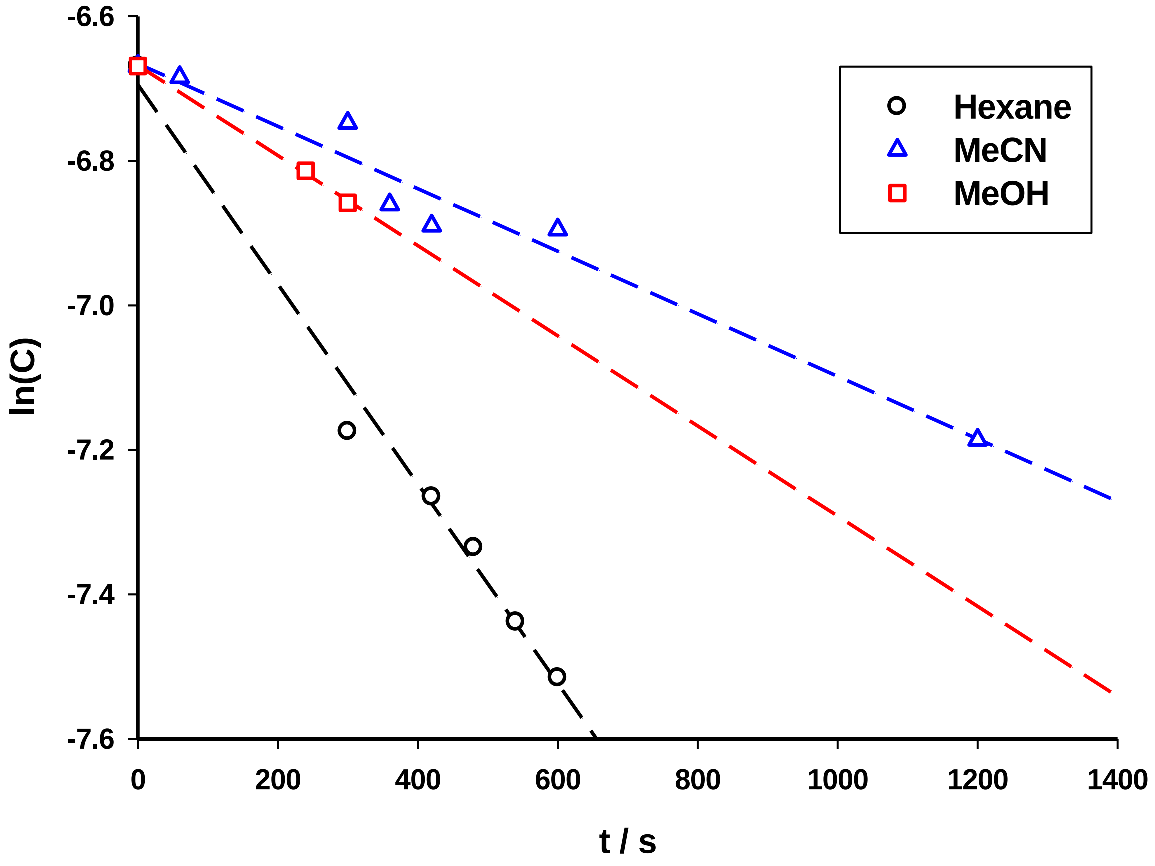

**Solvent**	k_{app} / s^{-1}	R^2
n-hexane	1.4×10^{-3}	0.9876
MeCN	4.3×10^{-4}	0.9647
MeOH	6.2×10^{-4}	0.9987

10. Emission spectra of CBD (2) in the examined solvents.

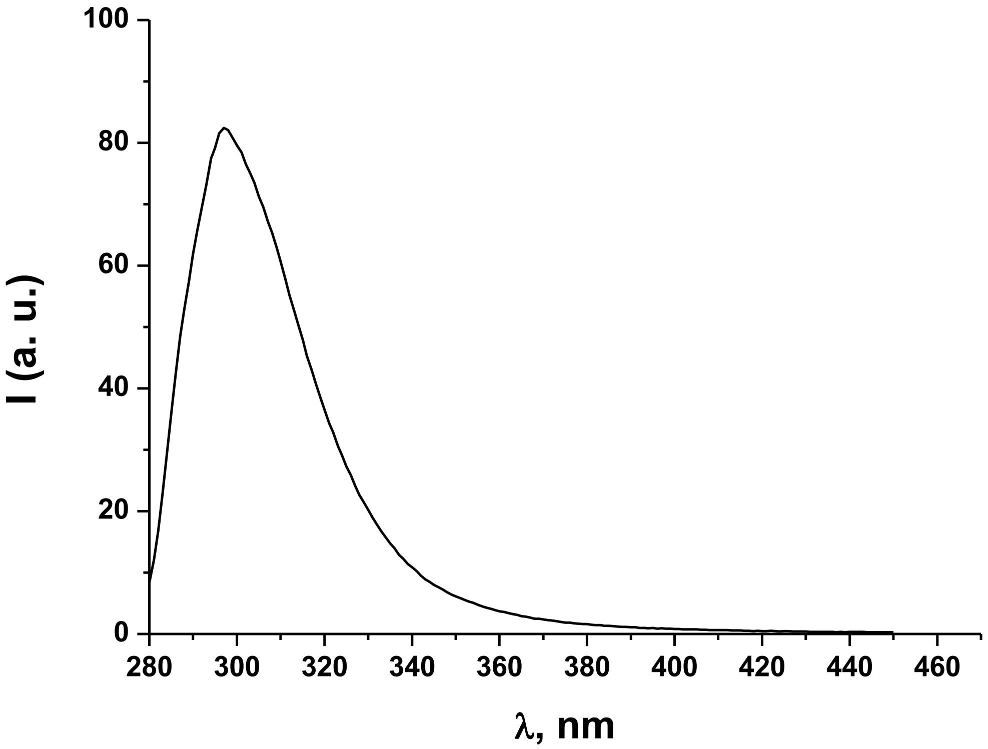

Figure 10.1. Emission spectrum of a solution of CBD (**2**) in MeCN.

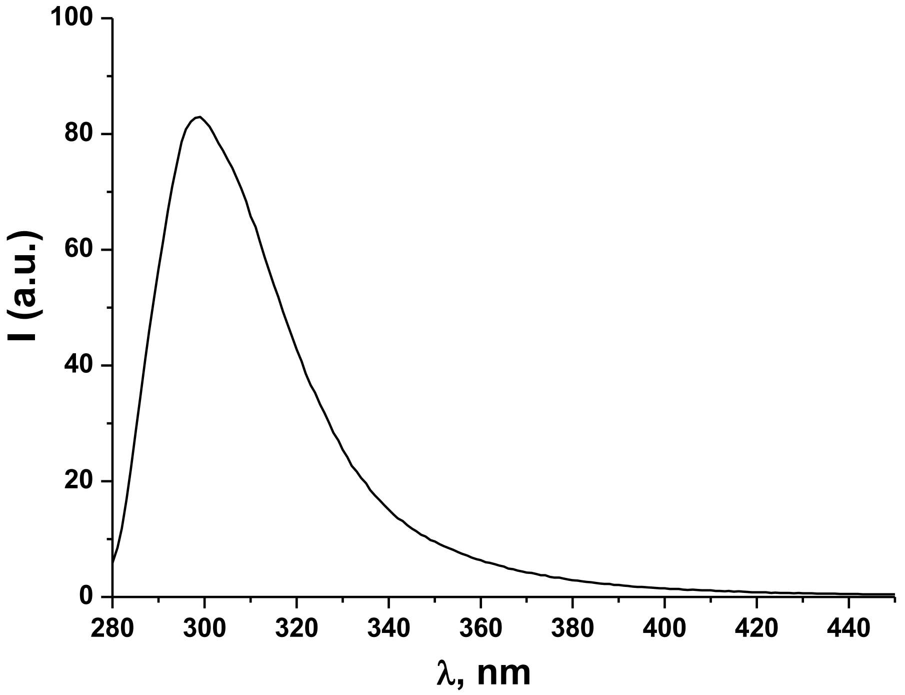

Figure 10.2. Emission spectrum of a solution of CBD (**2**) in MeOH.

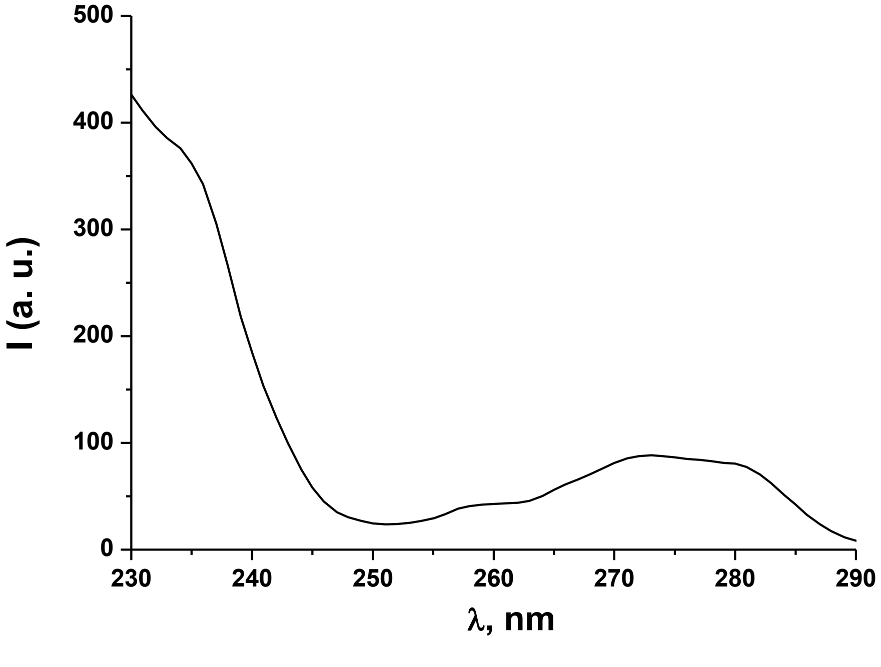

Figure 10.3. Excitation spectrum of a solution of CBD (**2**) in MeOH (λ_{ex} = 299 nm).

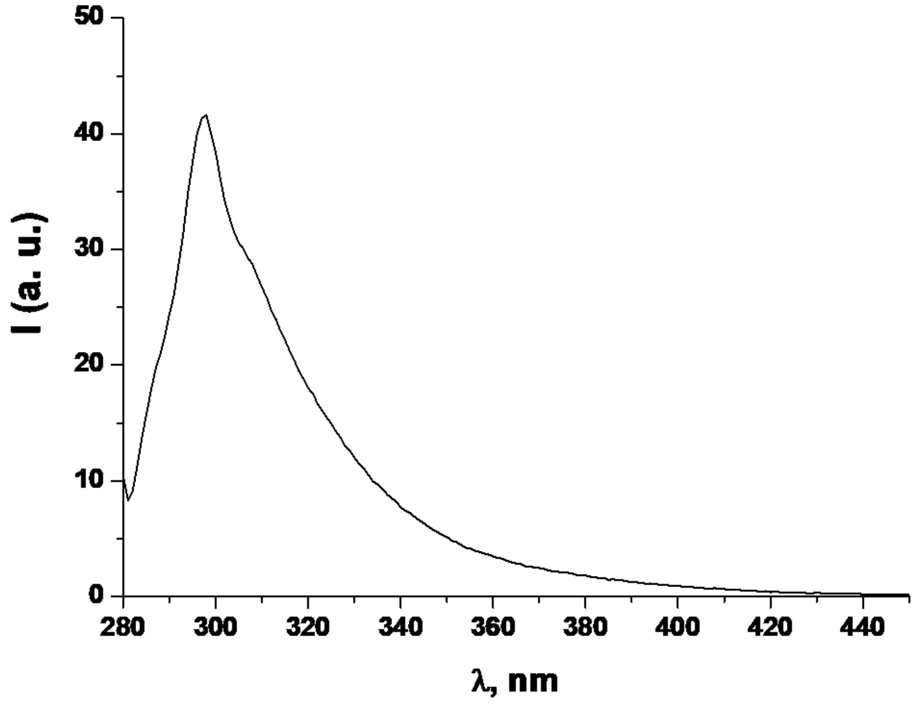

Figure 10.4. Emission spectrum of a solution of CBD (**2**) in *n*-Hexane.

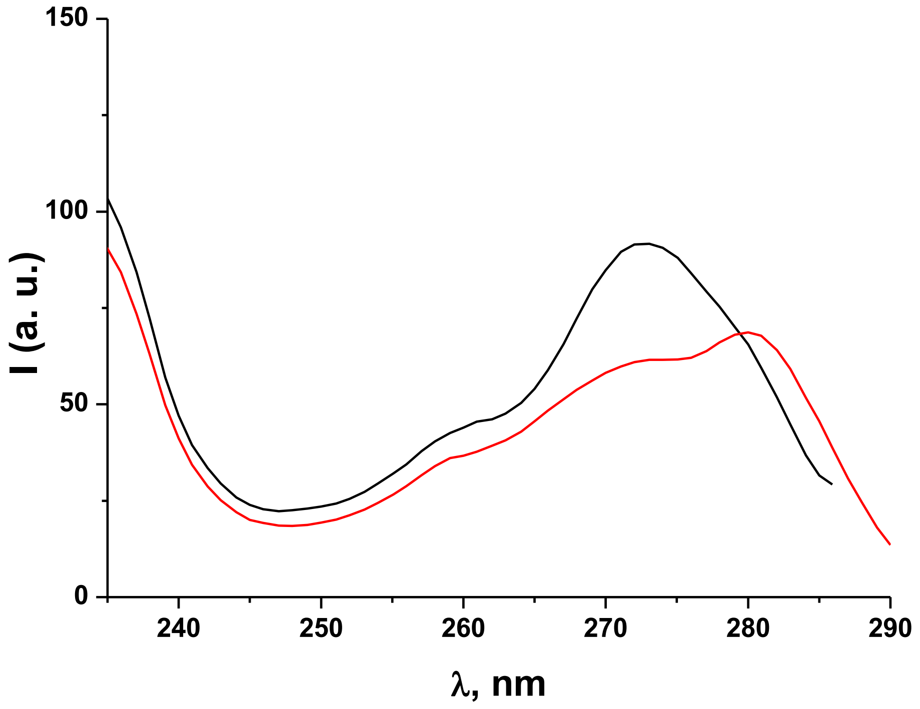

Figure 10.5. Excitation spectrum of a solution of CBD (**2**) in MeOH (λ_{ex} = 299 nm, black; (λ_{ex} = 310 nm, red).

11. References.

[S1] Villamor, J. L.; Bermejo, A. M.; Tabernero, M.J.; Fernandez, P. Determination of Cannabinoids in Human Hair by GC/MS. *Anal. Lett*. **2004**, *37,* 517-528.

[S2] De Brabanter, N.; Van Gansbeke, W.; Hooge, F.; Van Eenoo, P.; Fast quantification of 11-nor-D9-tetrahydrocannabinol-9-carboxylic acid (THCA) using microwave-accelerated derivatisation and gas chromatography–triple quadrupole mass spectrometry. *Forensic Science International*. **2013**, *224*, 90-95.

[S3] Foltz, R. L. Mass Spectrometric Methods for Determination of Cannabinoids in Physiological Specimens in ElSohly M.A. (eds) *Marijuana and the Cannabinoids. Forensic Science And Medicine.* Humana Press. https://doi.org/10.1007/978-1-59259-947-9_8
2007.

[S4] Nadulski T.; Sporkert, F.; Schnelle, M.; Stadelman, M.A.; Roser, P.; Schefter, T.; Pragst, F. Simultaneous and Sensitive Analysis of THC, 11-OH-THC, THC-COOH, CBD, and CBN by GC-MS in Plasma after Oral Application of Small Doses of THC and Cannabis Extract. *J. Anal. Toxicol.* **2005**, *29*, 782-789.

[S5] Lodewyk, M. W.; Lui, V. G.; Tantillo, D. J. Synthesis of (sulfonyl)methylphosphonate analogs of prenyl diphosphates, *Tetrahedron Lett.* **2010**, *51*, 170–173.

[S6] Yeom, H.-M.; Li, H.; Tang, Y.; Hsung, R. P.; Total syntheses of cannabicyclol, clusiacyclol A and B, iso-eriobrucinol A and B, and eriobrucinol. *Org. Lett.* **2013**, *12*, 3130-3133.

[S7] Crombie, L.; Ponsford, R.; Shani, A.; Yanitinky, B.; Mechulam R. Hashish components. Photochemical production of cannabicyclol from cannabichromene, *Tetrahedron Lett.* **1968**, *9*, 5771-5772.

[S8] Caprioglio, D.; Mattoteia, D.; Minassi, A.; Pollastro, F.; Lopatriello, A.; Munoz, E.; Tagliatela-Scafati, O.; Appendino, G. One-pot total synthesis of Cannabinol via Iodine-mediated Deconstructive annulation. *Org. Lett*. **2019**, *21*, 6122-6125.

[S9] Baek, S.-H.; Han, D. S.; Yook, C. N.; Kim, Y. C.; Kwak J. S. Synthesis and antitumor activity of Cannabigerol. *Arch. Pharm. Res.* **1996**, *19*, 228-230.

[S10] Choi, Y.H.; Hazekamp, A.; Peltenburg-Looman, A. M. G.; Frederich, M.; Erkelens, C.; Lefeber, A.W.M.; Verpoorte, R. NMR. Assignments of the major cannabinoid and cannabiflavonoids isolated from cannabis sativa. *Phytochem Anal*. **2004**, *15*, 345-354.

[S11] Crombie, L.; Ponsford, R.; Shani, A.; Yanitinky, B.; Mechulam, R. Hashish components. Photochemical production of cannabicyclol from cannabichromene. *Tetrahedron Lett*. **1968**, *9*, 5771-5772.

[S12] Budzikiewicz, H.; Alpin, R.T.; Lightner, D. A.; Djerassi, C.; Mechoulam, R.; Gaoni, Y. Massenspektroskopie und IHRE anwendung auf strukturelle und stereochemische probleme—LXVIII: Massenspektroskopische untersuchung der inhaltstoffe von haschisch *Tetrahedron* **1965**, *21*, 1881-1888.
